# Probing machine learning models based on high throughput experimentation data for the discovery of asymmetric hydrogenation catalysts†

**DOI:** 10.1039/d4sc03647f

**Published:** 2024-07-16

**Authors:** Adarsh V. Kalikadien, Cecile Valsecchi, Robbert van Putten, Tor Maes, Mikko Muuronen, Natalia Dyubankova, Laurent Lefort, Evgeny A. Pidko

**Affiliations:** a Inorganic Systems Engineering, Department of Chemical Engineering, Faculty of Applied Sciences, Delft University of Technology Van der Maasweg 9, 2629 HZ Delft The Netherlands e.a.pidko@tudelft.nl; b Discovery, Product Development and Supply, Janssen Cilag S.p.A. Viale Fulvio Testi, 280/6 20126 Milano Italy; c Discovery, Product Development and Supply, Janssen Pharmaceutica N.V. Turnhoutseweg 30 2340 Beerse Belgium llefort@its.jnj.com

## Abstract

Enantioselective hydrogenation of olefins by Rh-based chiral catalysts has been extensively studied for more than 50 years. Naively, one would expect that everything about this transformation is known and that selecting a catalyst that induces the desired reactivity or selectivity is a trivial task. Nonetheless, ligand engineering or selection for any new prochiral olefin remains an empirical trial-error exercise. In this study, we investigated whether machine learning techniques could be used to accelerate the identification of the most efficient chiral ligand. For this purpose, we used high throughput experimentation to build a large dataset consisting of results for Rh-catalyzed asymmetric olefin hydrogenation, specially designed for applications in machine learning. We showcased its alignment with existing literature while addressing observed discrepancies. Additionally, a computational framework for the automated and reproducible quantum-chemistry based featurization of catalyst structures was created. Together with less computationally demanding representations, these descriptors were fed into our machine learning pipeline for both out-of-domain and in-domain prediction tasks of selectivity and reactivity. For out-of-domain purposes, our models provided limited efficacy. It was found that even the most expensive descriptors do not impart significant meaning to the model predictions. The in-domain application, while partly successful for predictions of conversion, emphasizes the need for evaluating the cost–benefit ratio of computationally intensive descriptors and for tailored descriptor design. Challenges persist in predicting enantioselectivity, calling for caution in interpreting results from small datasets. Our insights underscore the importance of dataset diversity with broad substrate inclusion and suggest that mechanistic considerations could improve the accuracy of statistical models.

## Abstract 2

High-throughput experimentation and computational chemistry were used to build machine learning models for Rh-catalyzed asymmetric olefin hydrogenation, identifying numerous factors affecting the accuracy of selectivity and reactivity predictions.

## Graphical abstract

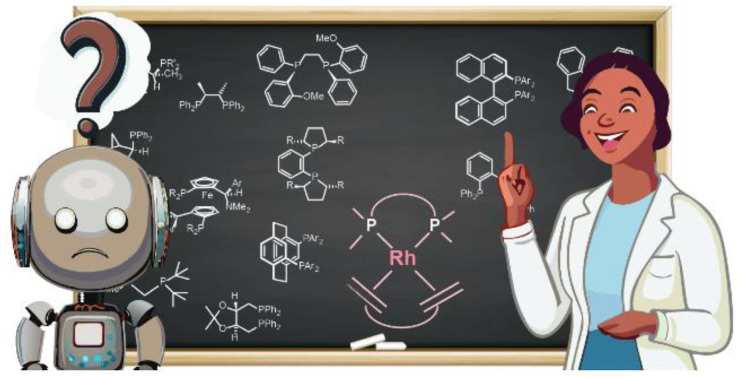

## Introduction

More than half a century ago, Knowles and Horner reported the first example of an enantioselective olefin hydrogenation catalyzed by Rh in combination with a chiral phosphine ligand.^1–3^ Although the obtained enantiomeric excesses were modest, their seminal work started the field. Asymmetric hydrogenation immediately appeared as an attractive method to produce enantiopure compounds.^4–9^ Compared to the competing classical resolution technology, it exhibits 100% theoretical yield, high atom economy, and good to excellent enantiomeric excesses. Over the last 50 years, the work from numerous industrial and academic groups resulted in the development of many efficient chiral ligands and in the implementation of this technology for large scale production.^10–17^ In addition to ligand development, the mechanism of this reaction was extensively studied *via* experimental^18–27^ and computational studies based on density functional theory (DFT)^28–31^ with the realization that the key elementary steps (*i.e.* the transition states governing selectivity and reactivity) vary with the ligands.

Despite the extensive knowledge built over the years, finding the right asymmetric hydrogenation catalyst for a new prochiral olefin remains a very empirical exercise and requires the screening of a large set of ligands and reaction conditions. High throughput experimentation (HTE) methodologies have successfully been implemented to rapidly explore the numerous parameters affecting the outcome of an asymmetric hydrogenation reaction.^32–39^ Nevertheless, integrating *in silico* assessments of catalyst candidates into HTE campaigns would be highly beneficial.^40^ It could further accelerate the time-sensitive process development of active pharmaceutical ingredients and lower the consumption of substrates needed to perform the HTE screening, often available in low quantity at the start of a drug development program. Unfortunately, the *in silico* design and development of homogeneous catalysts remains a challenging task.^41–43^ Predictive strategies for catalyst design are generally categorized into two groups depending on whether or not they require knowledge of the underlying mechanism of the catalytic cycle.^44–49^ The mechanism-based approaches rely on quantum chemical calculations of the key transition state intermediates and are very specific to the catalytic system under study. In addition, they are computationally expensive due to the complex energetic landscape of the transition metal-based catalysts. A few reports utilized this approach for the prediction of enantioselectivity of Rh based hydrogenation.^50–53^ To make mechanism-based approaches practical at a larger scale, potential energy functions of the reactants and products such as force-fields are used to approximate the connecting transition state.^45^ Recent implementations either mix the reactant and product potential energy surface with different weights/corrections to get an approximation of the stereo-determining transition state^50^ or utilize transition-state force fields to approximately describe the transition state directly.^45,51,53^

The alternative approach that does not require any knowledge of the mechanism is the use of quantitative structure–property relationships (QSPR).^54–60^ It consists in establishing a correlation between the structure of the catalyst and its performance *e.g.*, with regards to its activity or selectivity. Originating from the traditional linear free energy relationships (LFERs), such as Hammett plots,^61–63^ these methods have experienced a revival in the last decades with the advent of machine learning (ML) and its adoption by chemists.^46,55,64,65^ Refined catalyst representations based on quantum chemical calculations combined with more sophisticated statistical approaches are challenging the *status quo* of homogeneous catalyst design.^55,58,59^

Recent studies utilized this approach for the design of selective Rh-based catalysts.^66,67^ Xu *et al.* created a standardized database including over 12 000 data points on asymmetric hydrogenation of olefins from literature.^66^ This database was utilized in a hierarchical learning approach to connect a large amount of related data from literature to the small amount of data from ongoing experimentation campaigns. It was shown that this hierarchical approach performs well for predicting the selectivity of reactions with closely related substrates. The tested catalyst and substrate representations were limited to 2D and 3D cheminformatics-based descriptors. Recently, Singh *et al.* showcased an approach rooted in quantum chemistry,^67^ integrating quantum chemically derived molecular descriptors from five different asymmetric binaphthyl-derived catalyst families to predict the enantioselectivity of asymmetric olefin and imine hydrogenation. A random forest (RF) model trained on a set of 368 substrate–catalyst combinations demonstrated impressive predictive power compared to other linear and non-linear statistical methods, with a root-mean-square error in the predicted percent of enantiomeric excess (%*ee*) of about 8.4 ± 1.8 compared to experimental values.

Inspired by a recent publication of Sigman and coworkers together with Genentech,^68^ we decided to evaluate whether we could build a predictive model to support our HTE workflow for the screening of asymmetric hydrogenation catalyst. Our standard library of 192 chiral Rh catalysts was used to generate high quality data (up 3552 data points) as sole input for our model since it has been recognized that literature data could induce biases.^69,70^ In parallel, we developed a workflow to automatically generate a set of consistent DFT-based descriptors of our 192 ligands. Herein, we present our findings on the performance of our ML models in seven different cases including both out-of- and in-domain prediction tasks.

## HTE data generation and data reproducibility

Within our HTE group supporting chemical process development, we have developed several workflows to expedite the screening of catalysts. For asymmetric hydrogenation, we routinely screen two plates, each containing 96 chiral ligands, in combination with Rh. These ligands were selected based on their documented activity in asymmetric hydrogenation and their commercial availability. To complete the plates, we included several ligands not typically used in such reactions. In our ligand library, bisphosphines (denoted as ‘PP’ ligands) were the most prevalent, comprising 142 entries (74%), followed by aminophosphines (‘PN’) with 25 entries (13%), phosphoramidites with 11 entries (6%), and monophosphines (‘P’) with 10 entries (5%). Each ligand contained at least one phosphorus donor atom.

**Fig. 1:**
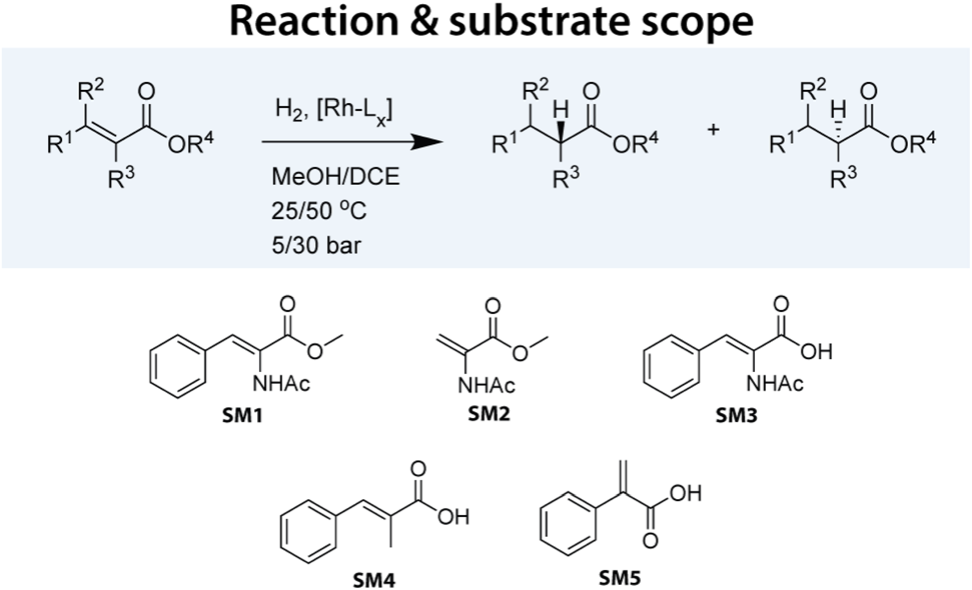
Asymmetric hydrogenation reaction performed in this study. A set of varying substrates was selected to be tested with a wide range of Rh-based catalysts under varying conditions.

In this study, we tested our 192 Rh precatalysts against five model substrates: SM1–SM3, representing some of the most significant substrates in the development of asymmetric hydrogenation, and SM4–SM5, which are structurally related and pose slightly greater challenges (see Fig. 1). Various substrates were tested under different reaction conditions. Although not a full factorial design, a total of 3552 data points were collected, representing to our knowledge the largest and most homogeneous dataset published for this crucial catalytic reaction. Table 1 summarizes the collected data points.

**Table 1:** Details of the 37 96-wells plates hydrogenation; data points selected for machine learning modeling in bold

Starting material	Solvent	*T* (°C)	*H* (bar)	Time (h)	#Data points
SM1	DCE	25	5	1	192
				16	192
			30	16	192
	Methanol	25	5	1	**192**
				16	576
			30	16	192
SM2	DCE	25	5	1	192
				16	192
	Methanol	25	5	1	**192**
				16	192
SM3	DCE	25	5	1	192
				16	192
	Methanol	25	5	1	**192**
				16	192
SM4	Methanol	50	5	16	**192**
SM5	Methanol	25	5	16	96
		50	5	16	**192**
Total					**3552 (*960*)**

To assess the stability of our Rh precatalysts, we evaluated the entire set with substrate SM1 immediately after their preparation, and again after six and twelve months of storage. Good reproducibility was observed for the enantiomeric excesses (ee), with coefficients of determination ranging between 0.87 and 0.94 across the experiments (see ESI, Fig. S3†). Out of 576 data points, only 38 showed a discrepancy where the absolute change in *ee* (|Δ*ee*|) measured in different runs exceeded 0.2.

As anticipated for a straightforward substrate like SM1, the conversion exhibited a strongly bimodal distribution, predominantly clustering around a value of 1 (indicating full conversion). To ensure a balanced classification, we categorized the data points into high conversion (conversion ≥0.8) and low conversion (conversion <0.8). Out of 192 ligands, only 20 of them exhibited a variation in classification across different runs, resulting in an average Pearson correlation coefficient of 0.86 across the three runs. The accuracy levels for pairwise comparisons ranged from 0.92 to 0.96. For detailed information on the experimental procedure we refer the reader to Section S1 of the ESI.†

## Data analysis

### Internal data analysis of experimental results

A total of 3552 data points were generated during the data production exercise across 37 96-well plates (Table 1). SM1, SM2, and SM3 were tested in 2 solvents (methanol (MeOH) and 1,2-dichloroethane (DCE)) and 2 reaction times (1 and 16 h). For SM1, higher pressure (30 bar instead of 5 bar) was also explored. Additionally, the more challenging SM5 was tested at 2 temperatures (25 °C and 50 °C).

**Fig. 2:**
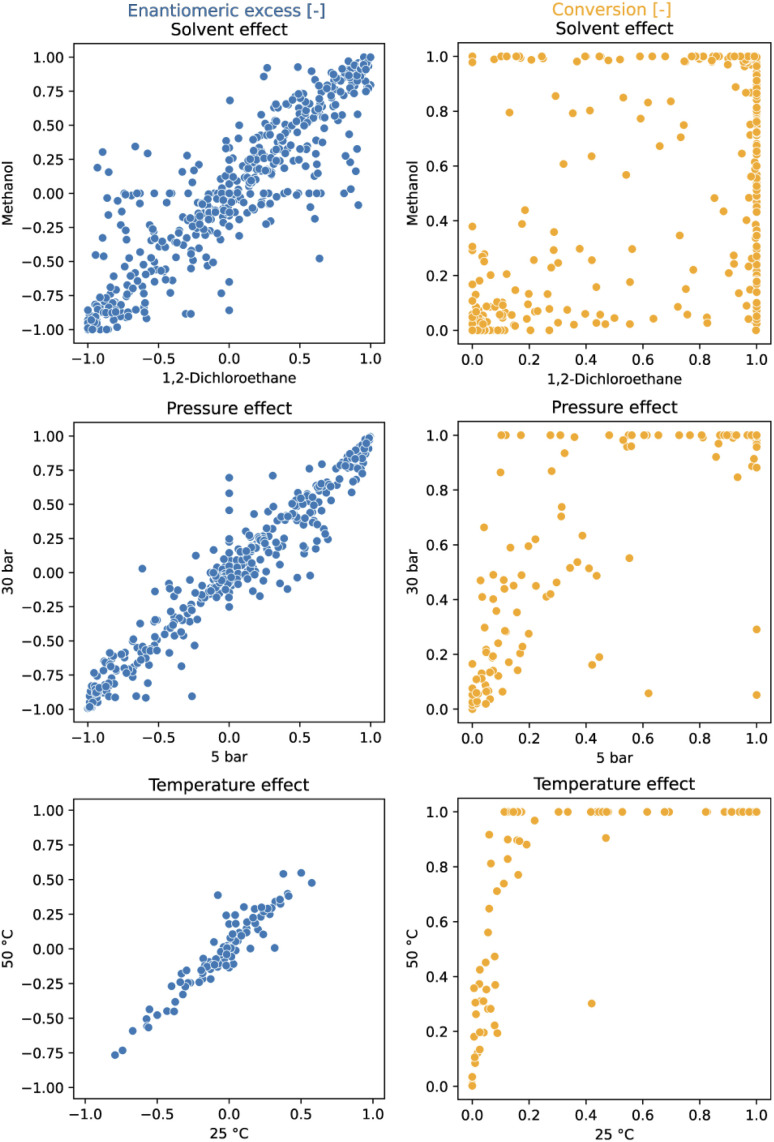
The influence of various conditions on reactivity (conversion) and enantioselectivity (*ee*) in Rh-catalyzed asymmetric olefin hydrogenation. Solvent effect was evaluated on SM1–SM3 after 1 h reaction time. Pressure effect was evaluated on SM1 after 16 h. Temperature effect was evaluated on SM5 after 16 h and on one plate. An interactive version of this figure displaying the ligand structures corresponding to the data points can be found in the ESI (see interactive figure ‘Fig. 2.html’ in the ESI†).

In addition to serving as high-quality input for machine learning models, this comprehensive dataset enables a systematic investigation of the effects of temperature, pressure and solvent across the entire set of ligands (see Fig. 2). At first glance, it appears that a variation of the reaction conditions has less influence on the ee than on the conversion. As expected, increased temperatures lead to higher conversion while exerting minimal impact on enantioselectivity. Elevated hydrogen pressures were found to generally improve conversion but adversely affected enantioselectivity for a few ligands.^18,27,71^ The solvent choice had the most significant effect on the performance; primarily on catalyst activity and, to a lesser extent, on enantioselectivity. These findings underscore the importance of solvent screening in HTE campaigns, advocating for its execution across a broad ligand set.

### Consistency analysis of experimental results and literature data

**Fig. 3:**
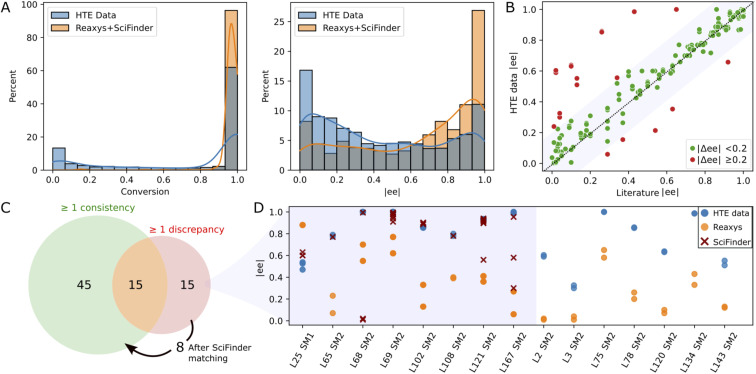
Consistency analysis: (A) raw distributions of conversion and |*ee*| in the current study (HTE data) – all data points in Table 1 – and in literature (reaxys + scifinder). (B) Scatter plot comparing the closest enantiomeric excess (|ee|) from literature with our experimental results under identical conditions (same catalyst, starting material, and solvent). (C) Venn diagram of ligand/substrate/solvent triplets divided into triplets with at least one consistent or discrepant reaxys record (green and red set, respectively). The arrow shows 8 triplets for which a consistency with scifinder was found. (D) Comparative analysis of |*ee*| discrepancies (|Δ*ee*| > 0.2) across our data (blue), reaxys (orange), and scifinder (red) for the 15 triplets for which no consistency with reaxys was found. An interactive version of this figure displaying the ligand structures corresponding to the data points can be found in the ESI (see interactive figure ‘Fig. 3.html’ in the ESI†).

Since the substrates of our study have been extensively studied in Rh-catalyzed asymmetric hydrogenation, we conducted a comparison between our experimental results and those documented in the literature. This endeavor necessitated the aggregation and refinement of published data, a process that proved to be challenging. Utilizing the reaxys database^72^ (accessed in March 2023), we performed a reaction search for the conversion of SM1–SM5 into their corresponding hydrogenated products, omitting stereochemistry. This search yielded 2098 references, each requiring diligent formatting and cleaning to standardize reaction component labels and conditions. After discarding entries with missing information and focusing exclusively on Rh-catalyzed reactions, we obtained a dataset comprising 752 entries. Notably, 566 of these entries matched ligands from our library in conjunction with either MeOH or DCE as solvents. In line with observations made by other research groups,^70,73^ our HTE campaign contained more negative results (low conversion and/or enantioselectivity) as compared to those reported in literature (see Fig. 3A), a disparity that augments the value of our dataset for machine learning model development.

The 566 literature entries comprised 75 unique ligand/substrate/solvent combinations, or “triplets”, involving 39 distinct ligands. We compared the *ee* values reported in literature with our experimental values for each triplet (see Fig. 3B). A “good match” was defined by an absolute *ee* difference of less than 0.2 (*i.e.*, |Δ*ee*| < 0.2). Among the 75 triplets, 45 exhibited complete concordance with no discrepancies observed between literature data and our experimental findings (see Fig. 3C). Furthermore, 15 triplets showed partial agreement, with at least one literature result aligning with our experimental data. Overall, 80% of the literature data sourced from reaxys aligned with our experimental results, reflected by a Pearson coefficient of 0.78. An additional search in scifinder (accessed in March 2023) targeting the 15 triplets with discrepancies unearthed 152 new literature entries, enabling the reconciliation for an additional 8 triplets (Fig. 3C and D), thereby elevating the agreement with published studies to over 90%. For the remaining 7 discrepancies, no scifinder records were found. As depicted in Fig. 3D, our *ee* values were consistently higher than those documented in literature. A closer examination revealed that all discrepant literature data emanated from a singular source,^74^ suggesting the possibility of a systematic experimental discrepancy within that study.

### Data selection for predictive models

After the analysis of the consistency of our HTE dataset, 960 data points out of 3552 were selected as inputs for our models (Table 1). Despite the common advocacy for larger datasets in enhancing statistical model performance, this selection was driven by the need for data uniformity and the avoidance of redundancies that might compromise model effectiveness. Moreover, we opted to exclude data from the literature, such as those from Xu *et al.*^66^ to prevent the introduction of inconsistencies, adhering to findings discussed elsewhere regarding data bias.^69,75–78^ In addition, this avoided the overrepresentation of a specific substrate. For simpler substrates SM1, SM2, and SM3, results from a 1 hour reaction time were chosen to ensure a balanced reactivity distribution. The empirically determined optimal temperature for each substrate was selected, and MeOH was consistently used as the solvent across all substrates to minimize variability from solvent effects, thereby allowing the model to more accurately focus on catalyst-specific features.

Considering the logarithmic nature of the enantiomeric excess, we chose to compute and model the ΔΔ*G*^‡^ values,^79^ which followed a distribution that approximates normality.^48^

**Fig. 4:**
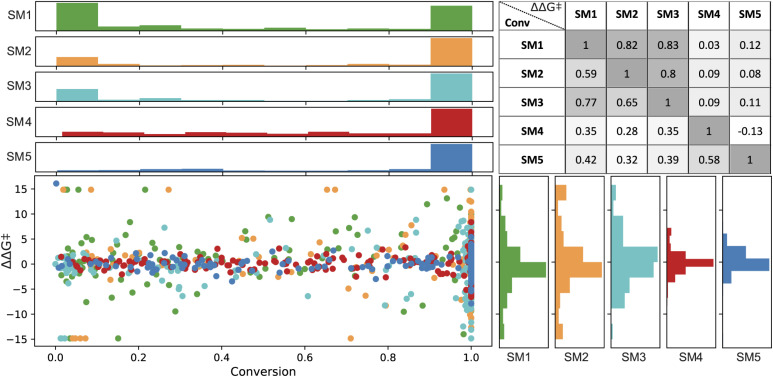
Distribution for conversion (%, on the top) and enantioselectivity (ΔΔ*G*^‡^ in kJ mol^−1^, on the right) in green, yellow, magenta, red and blue representing SM1, SM2, SM3, SM4, and SM5, respectively. The figure includes a Spearman correlation matrix of experimental values for substrate pairs, with the upper triangle showing ΔΔ*G*^‡^ and the lower triangle indicating conversion.

In the final set of 960 data points, enantioselectivity (ΔΔ*G*^‡^) demonstrated a distribution approximating a normal curve, with values ranging from −15 to 15 kJ mol^−1^ for SM1, SM2, and SM3, and −5 to 7 kJ mol^−1^ for SM4 and SM5.^80^ The conversion results exhibited a bimodal distribution (see Fig. 4), predominantly skewed towards higher values. Therefore, a classification model was built on a threshold of 0.8.

Spearman's rank correlation coefficient (see Fig. 4) was employed to evaluate and compare the catalyst rankings across different substrates. SM1 and SM3 showed the highest correlation in their experimental values, with correlation coefficients of 0.77 for conversion and 0.83 for ΔΔ*G*^‡^. This was followed by SM1–SM2 (correlations of 0.59 for conversion and 0.82 for ΔΔ*G*^‡^) and SM2–SM3 (correlations of 0.65 and 0.80, respectively). In contrast, SM4 and SM5 did not exhibit significant correlations with the other substrates. For instance, among the top performers in enantioselectivity for substrates SM1–SM3 are ligands (*R*,*R*,*S*,*S*)-DuanPhos (L55) and (*R*,*R*)-Et-DuPhos (L68), whereas for substrates SM4 and SM5, the ligands providing highest enantioselectivity are SL-J505-1 (L18) and a BoQPhos PN ligand (L186), respectively. Notably, L18 and L186 exhibit significantly lower selectivity with other substrates. As further detailed, conducting such an analysis provides critical insights for selecting training sets for out-of-domain tasks.

## Catalysts and substrate descriptors

The next step towards a statistical model involved the featurization, *i.e.*, mathematical representation, of the chemical entities. Descriptors were generated for the 192 catalysts and five substrates independently and then used as input features in our predictive models to encode the entire reaction space.^81^

### Ligand descriptors

The numerical representation of chemical entities is key to the quality of machine learning models. We decided to test three different representations with increasing simplicity for our set of 192 catalysts: DFT-based descriptors derived from DFT optimized geometries, 2D cheminformatics based on extended-connectivity fingerprints (ECFP4 with 512 bits) and one hot-encoding (OHE) of ligands and substrates.

For the DFT-based descriptors, we opted to compute well-established general descriptors for this reaction type without designing descriptors tailored to a specific reaction mechanism. We aimed at a state-of-the-art level of accuracy by performing DFT optimization with the PBE0-D3(BJ)/def2-SVP method (see ESI Section S3† for details) on all 192 Rh-precatalyst, *i.e.*, the cationic square planar [Rh(L)(NBD)]^+^ (NBD = Norbornadiene) formed upon mixing [Rh(NBD)_2_]BF_4_ with a chiral ligand and therefore reflecting the precatalyst state in the experimental catalyst library. In addition to the alignment with the experimental workflow, the rigid and symmetrical nature of NBD was key to limit the conformational freedom and reduce the computational cost while featuring a Rh-olefin interaction.^82^ Although this complex needs to lose NBD to enter the catalytic cycle, we anticipated that the descriptors derived from such a metal–ligand complex would be close to the catalytically-relevant states where a square planar complex with a P–Rh-alkene and P–Rh–O bond is formed in the transition state. A Python package, Open Bidentate Ligand eXplorer (OBeLiX),^44^ was developed to extract and calculate steric, geometric and electronic descriptors. A more detailed explanation about the featurization of the precatalyst structure is provided in Section S4 of the ESI.† Among other features, OBeLix utilizes a graph-based method to identify the ligand in the complex. This ensured that steric descriptors, such as the buried volume, were only taking the ligand into account. For the non-symmetrical bidentate ligands, the two coordinating atoms were distinguished based on their charge with the label min/max denoting the least/most positively charged donor atom, respectively. In addition to descriptors derived from the [Rh(L)(NBD)]^+^ complex, we also generated electronic descriptors for the ligand alone (labelled as ‘free ligand’). For this purpose, the ligand geometry was extracted from the optimized structure of the corresponding [Rh(L)(NBD)]^+^ followed by a single-point (SP) DFT calculation. This entire workflow resulted in a total of 101 descriptors per catalyst. Highly correlated descriptors as well as descriptors judged redundant based on our computational chemistry intuition (*e.g.*, Mulliken charges for atoms where NBO charges were already available) were removed leaving a final set of 34 descriptors per catalyst.^83^ This set contained 15 steric, 8 geometric and 11 electronic descriptors. The steric descriptors include percent buried volumes calculated with either the donor atoms or metal as the center of the sphere, measuring the steric hindrance induced by a ligand.^84,85^ The geometric descriptors include specific angles and distances, such as the bite angle,^86^ the cone angle, a dihedral angle of NBD with respect to the donor atoms and distances between the donor atoms and the metal center in the complex. The Tolman cone angle^87^ often serves as a method to assess the steric size of a ligand, however it was shown to be inaccurate for asymmetric ligands. The exact cone angle^88^ as implemented in Morfeus was used instead. Finally, the electronic descriptors set consist of commonly used descriptors derived from electronic structure calculations such as the HOMO–LUMO gap, NBO charges of the donor and metal atoms, and lone pair occupancies of donor atoms. These descriptors represent either the metal–ligand bonding or the local electronic environment within the complex. Similarly, electronic parameters were extracted from the SP DFT calculation on the free ligand.

In addition to the 3D representation, 2D ECFP representations were generated from the metal–ligand complexes using RDKit.^89^

Recently, Sigman and coworkers published descriptors for 111 ligands present in our library.^68^ Although these authors used Pd(L)(Cl)_2_ in their DFT calculations, their global geometric and electronic descriptors correlate well with our descriptors. However, large discrepancies were observed for the local steric descriptors that are more sensitive to the procedure for the initial structure generation and to conformers search and selection (see ESI Section S5† for a detailed comparison).

### PCA analysis of 3D DFT-based descriptors

**Fig. 5:**
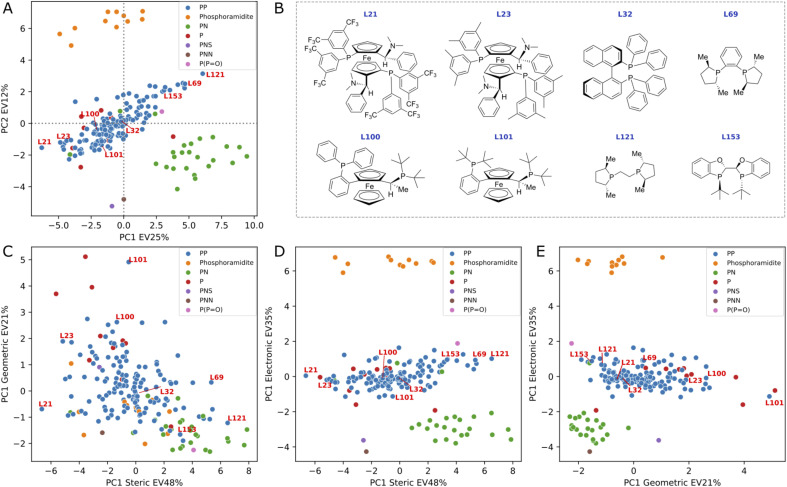
PCA score plot (A) and cross-sections (C–E) based on binning descriptors into three categories: steric, geometric and electronic. Eight bisphosphine ligands are included as example (B). Percent of explained variance (EV) is reported in the axis label.

We conducted a principal component analysis (PCA) on our dataset of DFT-based descriptors to investigate whether a dimensionality reduction would allow a visualization of the ligand space that aligns with human chemical intuition and understanding. Applying PCA directly to the 34 descriptors and selecting the first two principal components, which explain 37% of the variance, resulted in the formation of well-defined clusters (see Fig. 5A) corresponding to chemically distinct classes of ligands (*e.g.*, phosphoramidites, PN ligands, phosphine oxide). As expected, the loading plots for the first two principal components revealed a predominant influence of electronic descriptors, confirming that the clustering was primarily based on electronic differences (see ESI Fig. S11†).

In an effort to discern smaller clusters, subsequent PCA analyses were performed on each category of descriptors (namely, the electronic, steric and geometric descriptors). The first principal component from each category was then used to construct cross sections and the results are summarized in Fig. 5B–E (see PCA interactive figure in the ESI† for enhanced visualisation and analysis). The first component within each category accounts for a significant proportion of the variance explained by the descriptors, with values of 48%, 35%, and 21% for steric, electronic, and geometric descriptors, respectively. The cross section derived from geometric and steric descriptors clearly demonstrates that families of chemically distinct ligands occupy the entire geometric/steric space. The structures of a few ligands and their placements on the four PCA maps are depicted in Fig. 5. Notably, similar ligands, such as the MandyPhos (L21 and L23) and phospholane-based ligands (L69 and L121), are positioned in close proximity to one another. Along the steric axis, the bulky MandyPhos ligands (L21 and L23) are contrasted with the smaller DuPhos (L69) and BPE (L121), aligning with chemical intuition. Less intuitively, the WalPhos ligand (L101), characterized by two di-*tert*-butyl-phosphino groups and likely a large cone angle or Rh–P distance, is located at the extreme of the geometric axis. L100, another WalPhos ligand with only one di-*tert*-butyl-phosphino group, is appropriately positioned slightly below L101. At the opposite end of the geometric axis lies L153, a BIBOP ligand, presumably due to its compactness and small bite angle. These PCA maps could facilitate a data-driven approach for selecting a chemically diverse set of ligands for experimental screening. However, it is noteworthy that privileged structures,^90^ such as for example the BINAP ligand (L32), are located in the center of all maps and thus are not distinguished by this methodology.

### Substrate descriptors

A static representation of all five substrates, SM1–SM5, was generated using four sets of descriptors: 3D DFT-based steric fingerprint, 3D SMILES-based steric fingerprint, ECFP and OHE. The steric fingerprints aimed to describe the local steric environment surrounding the olefinic bond. They were either created from a DFT optimized structure of the substrate alone or from a 3D structure generated by Openbabel^91^ based on the SMILES of the substrate. The carbon atoms involved in the double bond (denoted as *C*_1_ or *C*_2_) as well as those directly connected to them were enumerated (denoted as *R*_1_ to *R*_4_). A buried volume for all these atoms and sterimol parameters (B1, B5 and L) for each possible C and R pairing were calculated, resulting in a fingerprint consisting of six buried volumes and 12 sterimol parameters (see our Github repository of the published ML pipeline for more details and code).

## In/out domain modeling

**Fig. 6:**
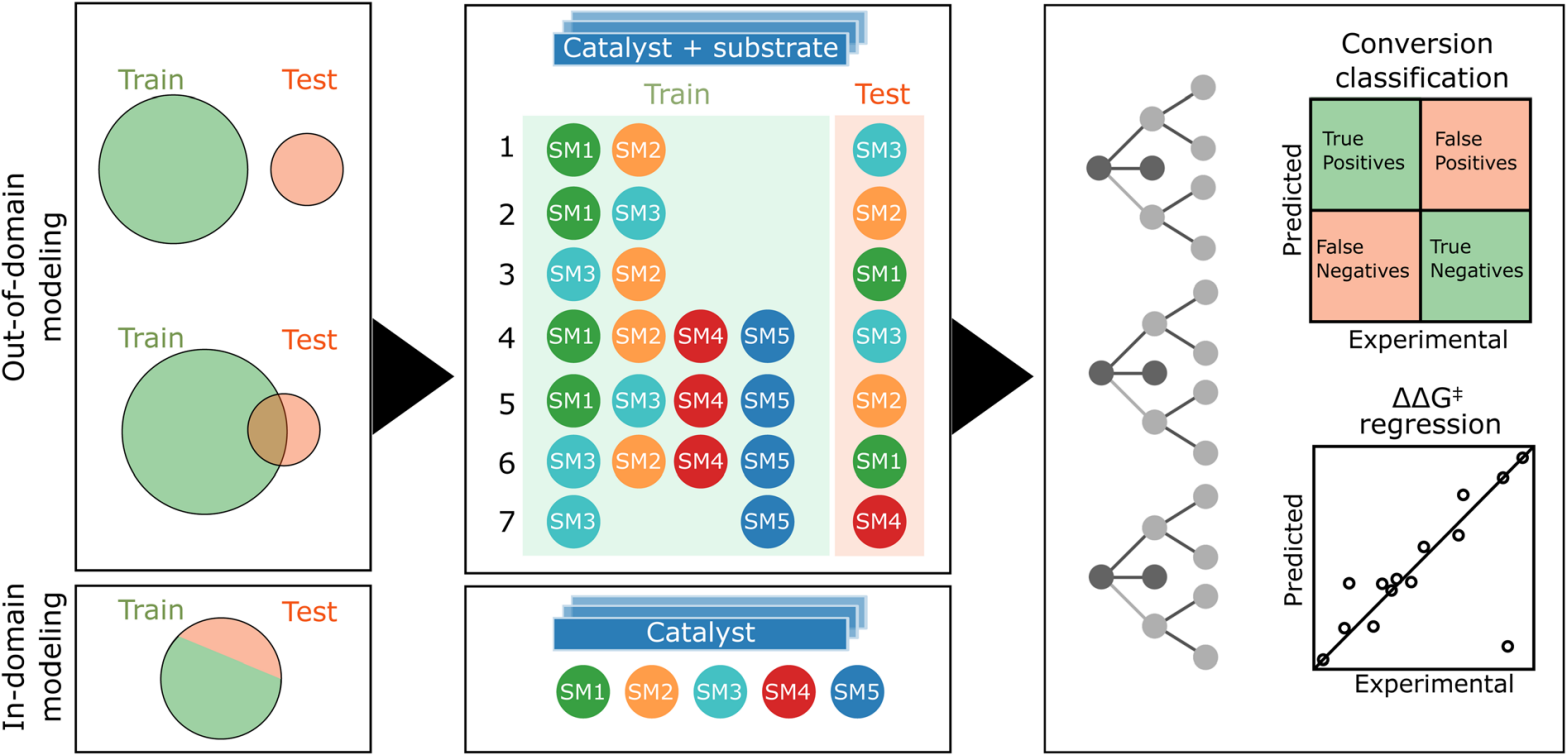
Schematic representation of the machine learning workflow. In both fully and partially out-of-domain modeling scenarios, for each target starting material (SM), the model is trained on data from at least two additional SMs in accordance with seven specific cases. The feature matrix, is formed by concatenating descriptors of both catalyst and starting material. In partially out-of-domain modeling, half of the target SM samples are included in the training set. For in-domain tasks, each SM model undergoes training with an 80 : 20 training-test split, focusing solely on catalyst descriptors. We use of random forest for classification (reactivity) and regression (selectivity).

As previously mentioned, our experimental data exhibits a bimodal distribution for conversion, biased towards higher conversion. Consequently, we opted for a classifier to model catalyst activity. The distribution of enantiomeric excesses (ΔΔ*G*^‡^) displayed a more normal pattern, rendering it suitable for regression analysis. Given the dataset encompassing five substrates (SM1–SM5) and 192 catalysts, our study presented a unique opportunity to explore both out-of-domain and in-domain modeling (see Fig. 6).

In the out-of-domain approach, which would constitute the most impactful scenario, samples of the target starting material are excluded from the training dataset to evaluate the model's ability to predict reactivity and selectivity for starting materials that it has not encountered. This method involves numerically encoding both catalysts and substrates, followed by concatenating these encoded representations. The theoretically most informative feature set combines DFT-based descriptors for ligands with DFT-based steric fingerprints for substrates. Conversely, the simplest feature combination employs one-hot encoding for both ligands and substrates. Additionally, we explored the scenario when only a half of the target starting material's samples is included in the training set, simulating the use of first HTE plate results before running a second plate. This approach is referred to as partially out-of-domain modeling. For conversion classification, we set a common threshold of 0.8 as the best balance between class distribution across substrate data.

During our data analysis, we observed varying levels of correlation of the results obtained with the five starting materials (see Fig. 4). This prompted us to investigate the impact of training set and target correlations on out-of-domain model accuracy. We detailed seven specific cases in Fig. 6. Cases 1–3 focused solely on the related starting materials SM1, SM2, and SM3, using only the two most closely related substrates for training when predicting the target substrate's behavior. Cases 4–6 were still focused on predicting SM1, SM2, SM3 but with the inclusion of additional, less related substrates (SM4 and SM5) into the training set. Here, the goal was to assess the effect of substrate set diversity on model performance. Case 7 posed a more complex challenge, aiming to predict reactivity and selectivity for SM4 using the unrelated substrates SM3 and SM5 for training.

In the in-domain modeling, the objective is to predict catalyst performance for a specific substrate, employing a portion of the 192 results for model training and the remainder for evaluation. The conversion classifier's threshold is set based on the median conversion specific to each substrate.

The efficacy of both out-of-domain and in-domain modeling approaches significantly depends on the choice of catalysts included in the training set. To mitigate this dependency and ensure the robustness of our findings, we tested three distinct random splits of the train/test set for each case. Hyperparameter tuning *via* a grid search within a predefined parameter space and *k*-fold cross-validation were performed for each split (see ESI Section S8† for more details on our ML pipeline).

Following preliminary screening with automated machine learning tools such as Auto-Sklearn^92^ and TPOT,^93^ we chose Sklearn's random forest implementation as our study's algorithm.^94,95^ Random forest, an ensemble learning algorithm, harnesses multiple decision trees and randomness to construct a predictive model capable of handling diverse data types and excelling in classification and regression tasks.^67,96^

Furthermore, correlation analysis (see Excel file with experimental data and descriptors in the ESI†), revealed a limited univariate linear correlation (maximum absolute Spearman correlation coefficient of 0.58) between conversion and the DFT-based descriptors. The correlation of these descriptors with enantioselectivity was generally weaker (maximum absolute Spearman correlation coefficient of 0.15). A preliminary in-domain linear regression modeling (see ESI Jupyter notebooks and pickle files with the final results in the ESI†) failed to accurately predict conversion and ΔΔ*G*^‡^, further justifying the selection of the Random forest algorithm.

Considering four possible representations for the five starting materials, three ligand representations, seven cases and the prediction of both conversion and enantioselectivity, we trained in total over 700 models including 168 models for the fully out-of-domain task, 504 for the partially out-of-domain task (across three different training-test splits), and 90 for the in-domain task. To evaluate the predictive performance of our classifiers and regressors, we calculated the balanced accuracy (BA) and the coefficient of determination (*R*^2^ score), respectively. BA is the average of recall obtained on each class and it ranges between 0 and 1 with 1 being the desired outcome (see the Data availability statement for more information on all code and data).

## Results and discussion

### Machine learning predictions

#### Out-of-domain approaches

**Fig. 7:**
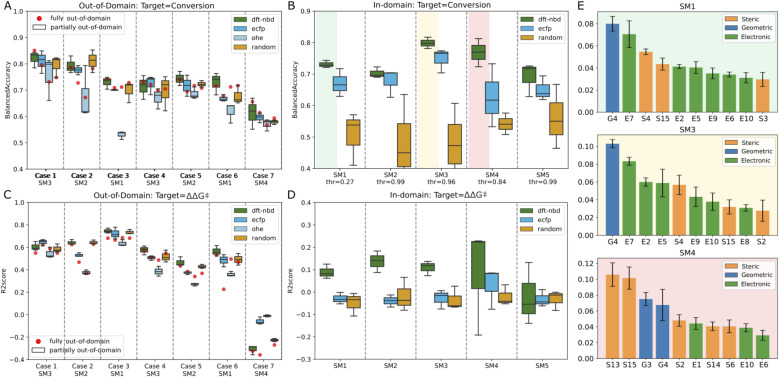
Performance metrics for out-of-domain and in-domain modeling. Panel A and C display the balanced accuracy and R2 score for out-of-domain modeling (A: Conversion; C: ΔΔ*G*^‡^), while Panel B and D illustrate the same for in-domain modeling (B: Conversion; D: ΔΔ*G*^‡^). In A and C the starting material's representation is one-hot encoded. Fully out-of-domain results for DFT-based descriptors are represented by red dots. E: Gini feature importance for RF in-domain classifiers trained on DFT-based descriptors to model conversion.

The performance metrics of the models for the out-of-domain task are presented in Fig. 7A and C. For reactivity modeling, we observed a BA ranging from 0.58 (case 7) to 0.85 (case 1, modeling SM3) with DFT-based descriptors for the catalysts, indicating that the models are able to estimate the experimental results with reasonable accuracy. Notably, SM3 modeling, when incorporating non-correlated substrates in case four, resulted in a marked decrease in performance (from 0.85 case 1 to 0.73 case 4), whereas this impact was minimal for other substrates (SM2, from 0.78 case 2 to 0.74 case 5; SM1, from 0.74 case 3 to 0.72 case 6). For the selectivity modeling using DFT-based descriptors for the catalysts, the highest *R*^2^ score of 0.68, was observed in case 3.

In this scenario, the target substrate SM1 exhibited a strong experimental correlation (ΔΔ*G*^‡^) with SM2 and SM3, as indicated by Spearman correlation scores of 0.82 and 0.83 respectively. Conversely, the performance of the model declined when introducing unrelated substrate in the training set (case 6: *R*^2^ score of 0.53) and was very poor when trying to estimate the unrelated substrate SM4 (case 7: *R*^2^ score of −0.3). Incorporating half of the catalyst set for the target substrate (partially out-of-domain task) generally did not significantly impact the balanced accuracy or the *R*^2^ score (see Fig. 7A and C, box plots). Indeed, the box plots obtained from random splits of the training set in the partially out-of-domain task almost always contain the red dots of the fully out-of-domain tasks.

Surprisingly, the more straightforwardly computed ECFP and trivial OHE exhibited BAs for conversion that were largely consistent with those of the more costly DFT-based descriptors both for the fully and partially out-of-domain tasks. For case 2 and 3 only, the performance of OHE was notably inferior, suggesting that these models are influenced by the selection of training sets and hyperparameters. To mitigate overfitting, we restricted the maximum depth of the trees. This constraint, however, can be a bottleneck for OHE, adversely affecting its effectiveness, therefore for OHE only we let the model expand nodes until all leaves are pure. Models built on ECFP and OHE features, demonstrated performance that was for most cases comparable to DFT-based descriptors. To further examine the influence of catalyst featurization, we introduced a new set of ligand descriptors consisting of 34 randomly generated values ranging from −100 to +100 for each ligand. A total of 192 vectors of random descriptors were generated and assigned to each ligand. In the out-of-domain modeling where multiple substrates are considered, a single ligand appears multiple times and was consistently represented by the same random vector. Interestingly, these random descriptors achieved the same performance as the DFT-based descriptors, with BAs ranging from 0.59 to 0.78 across the seven cases and *R*^2^ score between 0.37 (case 5) and 0.68 (case 3) for the first six cases and a drop in performance for case seven. The maximum disparity noted in outcomes derived from DFT-based descriptors *versus* random descriptors within the fully out-of-domain approach is 0.06, as observed for case 7 for modeling conversion. These data suggests that our generated DFT-based descriptors were not able to capture the essential chemistry in this dataset as they do not impart significant meaning to the model estimates. The poorer performance demonstrated by OHE in certain cases suggests that binary descriptors may be less informative or less prone to chance correlations compared to other descriptor types. In general, the model's effectiveness varied notably across different cases. The ability to estimate the experimental values correctly appears to be more related to the inherent correlation of catalyst performance across different substrates, rather than the intrinsic value of the descriptors. For instance, modeling SM4 using non-correlated substrates (SM3 and SM5) in case 7 proved unsuccessful (Fig. 7A and C), while outcomes based on correlated substrates (as in case 1) were more accurate (average BA for conversion of 0.8 and average *R*^2^ score for selectivity prediction of 0.6). This outcome highlights that our machine learning models primarily estimates based on the principle of “what works now, will work in other cases” and *vice versa* (see ESI Fig. S13†). Adding partial information about the target substrate to the training set (partially out-of-domain modeling) did not significantly enhance the accuracy nor alter the behavior of the model. In other words, a limited introduction of target substrate information does not substantially influence the performance of our models.

#### In-domain approach

Confronted with the unsuccessful out-of-domain modeling task, our focus shifted towards in-domain modeling, *i.e.*, a more manageable, albeit less valuable, endeavor, in line with recent literature.^58,59,68^ The objective was to test whether models constructed within a substrate-specific context, thus trained solely on catalyst descriptors, could effectively discern meaningful information. An additional goal was to assess whether DFT-based descriptors would introduce superior chemical information into the model compared to ECFPs and random descriptors. Results are summarized in Fig. 7B and D. For reactivity, modeling using random descriptors achieved a BA around 0.5. Models utilizing DFT-based descriptors and ECFPs exhibited superior performance across all substrates. Specifically, for substrates SM1, SM3, and SM4, DFT-based descriptors surpassed ECFPs, achieving average BAs of 0.73, 0.80, and 0.77 on the test set, respectively, compared to ECFPs average BAs of 0.64, 0.71, and 0.63. For substrates SM2 and SM5, the performance of ECFPs was comparable to DFT-based descriptors, recording BAs of 0.65 *versus* 0.66 and 0.67 *versus* 0.69, respectively.

For SM1, SM3 and SM4, we investigated the feature importance of the trained models (see Fig. 7E). Roughly, the same features are present for the related substrates SM1 and SM3 while different features are used by the model for unrelated SM4. For SM1 and SM3, most of the descriptors are electronic, for example, E7 (lone pair occupancy of the min donor atom calculated on free ligand), E5 (NBO charge of metal center) and E10 (NBO charge of the max donor atom calculated on the free ligand). The only geometric descriptor present in all four models is G4 (distance between Rh and the minimum donor). S15 (buried volume on minimum donor atom) seems to be the most important steric descriptors. Overall, it is difficult to derive any meaningful mechanistic considerations from these observations.

In-domain modeling for enantioselectivity was unsuccessful, as evidenced by *R*^2^ scores not exceeding 0.2 on the test set. DFT-based descriptors for substrates SM1, SM2, and SM3 showed marginally better results than other descriptors. To investigate whether best-performing models for enantioselectivity could reside within smaller subsets of related catalysts, we implemented a Monte-Carlo data selection approach. This involved testing 1000 random splits for each catalyst fraction, ranging from 90% to 10% of the entire catalyst set in decrements of 10%. Each subset was divided into an 80 : 20 training-test ratio, and RF models were trained exclusively using DFT-based descriptors. Our findings indicate the feasibility of deriving models with high *R*^2^ scores (up to 0.98) on sets comprising merely 10% of the catalysts (only 25 and 4 data points in training and test set, respectively, see ESI Fig. S14†). However, the lack of discernible pattern differentiating these catalysts from others, *e.g.* by ligand family or class, suggests that such high scores are solely due to chance correlation and test overfitting. This approach, deviating from standard machine learning practices, was employed to demonstrate the potential pitfalls when working with small datasets in machine learning pipelines, highlighting the risk of uncovering spurious, albeit appealing, correlations.

## Conclusions

Using our high throughput experimentation workflow, we have generated and made available a large and reliable dataset of asymmetric hydrogenation results, encompassing most of the commercially available chiral ligands. Our experimental results align well with existing literature, affirming their validity but substantially exceed those in uniformity and comprehensiveness. Discrepancies observed were meticulously analysed and satisfactorily accounted for, ensuring the robustness of our dataset.

We proved that the application of machine learning modeling to estimate the reactivity of an unseen substrate relies solely on ligand differentiation with only a marginal improvement in performance observed for our set of DFT-based descriptors in the fully out-of-domain task for three out of seven cases. Several factors may contribute to this outcome. The dataset encompasses a limited range of substrates (five) and catalyst variability (192). This constraint hinders the models' ability to effectively interpret and differentiate features based on meaningful chemical properties. Instead, the models tend to rely on mere object differentiation, which explains why random descriptors exhibit performance levels comparable to those of more expensive DFT-based descriptors. The outcomes of our models can be explained by the fact that we derived our descriptors from the precatalyst with the goal to produce models with broad applicability. However, the combination of the reaction constituents, *i.e.*, [Rh(L)(NBD)]^+^ and substrate, may not be descriptive of reactivity-/stereo determining steps in the reaction mechanism. Given the current dataset, considering a transition state of catalysts with a specific substrate might be a more accurate alternative for modeling, albeit more computationally intensive and lacking the desired generality.

The “in-domain” strategy, while successful to some degree in modeling conversion, did not perform as well as anticipated. For certain substrates, we observed that ECFPs descriptors performed similarly to our DFT-based descriptors, indicating that our generated DFT-based descriptors might not significantly impact the model's effectiveness in certain scenarios. We acknowledge that our general, but still computationally intensive descriptors do not automatically outperform descriptors computed through more simplistic, naïve methods. This observation underscores the necessity for both a critical evaluation of the computational cost–benefit ratio and a tailored approach in the selection and engineering of descriptors for specific applications.

Enantioselectivity modeling remains a considerable challenge. We found no discernible correlations between our DFT-based descriptors and ΔΔ*G*^‡^, especially when working with small datasets (fewer than 20 data points). In such datasets, any strong correlations observed are likely due to chance, underscoring the necessity for cautious interpretation of results.

The insights garnered from our study highlight the importance of dataset diversity and mechanistic insights. Our findings suggest that expanding the dataset to include a broader range of substrates and ligands and applying an *ad hoc* DFT-based feature engineering process could potentially enhance model performance, particularly in out-of-domain scenarios. The library of 192 Rh catalysts is regularly being tested for new substrates of our development pipeline. The generated data are used to augment the dataset of the model with more diverse chemical entities. In addition, we are exploring alternative modeling approaches and new descriptors that might better capture the complexities of reactivity and selectivity modeling. Once our model will exhibit a high level of accuracy, we will include the screening of virtual ligands generated *via in silico* modification of the existing ones^44^ with the ambition to discover entirely new asymmetric hydrogenation catalysts.

## Author contributions

A. V. Kalikadien and C. Valsecchi contributed equally to this work. A. V. Kalikadien: conceptualization, methodology, software, validation, formal analysis, investigation, data curation, writing – original draft, writing – review & editing, visualization, project administration C. Valsecchi: conceptualization, methodology, software, validation, formal analysis, investigation, data curation, writing – original draft, writing – review & editing, visualization, project administration R. van Putten: conceptualization, methodology, validation, formal analysis, investigation, data curation, writing – original draft, writing – review & editing, visualization T. Maes: conceptualization, writing – review & editing M. Muuronen: conceptualization, methodology, writing – review & editing N. Dyubankova: conceptualization, writing – review & editing, supervision L. Lefort: conceptualization, methodology, validation, resources, writing – original draft, writing – review & editing, visualization, supervision, project administration, funding acquisition E. A. Pidko: conceptualization, methodology, validation, resources, data curation, writing original draft, writing – review & editing, supervision, project administration, funding acquisition.

## Conflicts of interest

There are no conflicts to declare.

## Supplementary Material

SC-015-D4SC03647F-s001

SC-015-D4SC03647F-s002

SC-015-D4SC03647F-s003

SC-015-D4SC03647F-s004

SC-015-D4SC03647F-s005

SC-015-D4SC03647F-s006

## Data Availability

The used machine learning pipeline can be accessed on the Github organization page of the ISE group at TU Delft: EPiCs-group (https://github.com/EPiCs-group/obelix-ml-pipeline). Similarly, the used Python package for featurization of catalyst structures, OBeLiX, can be accessed on the Github organization page of the ISE group at TU Delft: EPiCs-group (https://github.com/EPiCs-group/obelix). In addition to this manuscript, ESI† and all used datasets are available together with an extensive readme *via* 4TU.ResearchData at https://doi.org/10.4121/ecbd4b91-c434-4bdf-a0ed-4e9e0fb05e94: • List and visualization of ligands (‘ligand_list.pdf’) • Interactive figures (‘Fig. 2.html’,‘ Fig. 3.html’ and ‘PCA.html’) • DFT input and output files for metal–ligand complexes and extracted free ligand structures (‘nbd_metal_ligand_dft_output.zip’ and ‘free_ligand_extracted_from_dft_output.zip’) • Excel file with experimental data and descriptors (‘C=C_AH_dataset.xlsx’) • Excel file with ML results (‘ml_results_tables.xlsx’) • Jupyter notebooks and pickle files with the final results (‘data_analysis.ipynb’, ‘Literature_comparison_Reaxys_SciFinder.ipynb’, ‘dft_nbd_model_literature_comparison.zip’, ‘view.ipynb’, ‘dict_res_obj1.pkl’, ‘dict_res_obj2.pkl’, ‘dict_res_obj3.pkl’, ‘dict_res_obj4.pkl’).
