# Supplementary material for: Probing machine learning models based on high throughput experimentation data for the discovery of asymmetric hydrogenation catalysts
Source: Chem Sci. 2024 Jul 16. Online ahead of print. doi: 10.1039/d4sc03647f (PMC11352728; doi:10.1039/d4sc03647f)
Supplement: SC-OLF-D4SC03647F-s004 [file SC-OLF-D4SC03647F-s004.pdf]

Electronic Supplementary Material (ESI) for Chemical Science.
This journal is © The Royal Society of Chemistry 2024

Definitive list of ligands (Oct '23)

#	Structure	Alias or name	CAS#	Type	Formula	Eq. to Rh	M_w [g mol^{-1}]	Vendor	Catalog #
L1	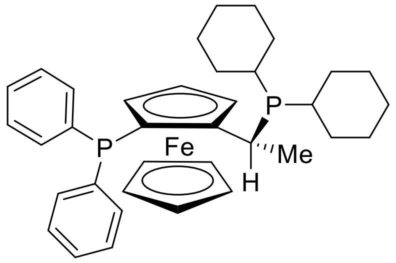	SL-J001-1	155806-35-2	PP	$C_{36}H_{44}FeP_2$	1.01	594.54	Sigma-Aldrich	88717
L2	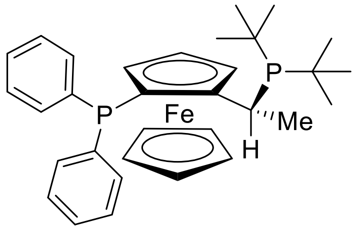	SL-J002-1	155830-69-6	PP	$C_{32}H_{40}FeP_2$	1.01	542.46	Sigma-Aldrich	88719
L3	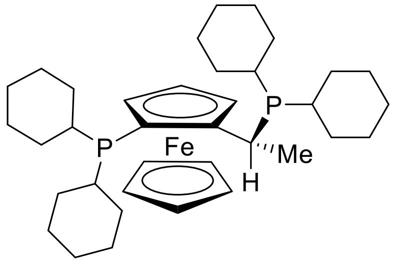	SL-J003-1	167416-28-6	PP	$C_{36}H_{56}FeP_2$	1.04	606.64	Sigma-Aldrich	88721
L4	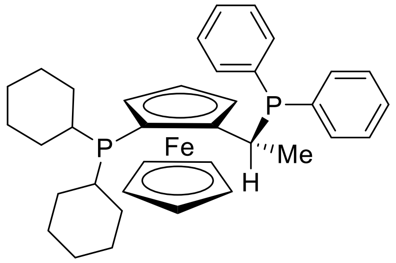	SL-J004-1	158923-09-2	PP	$C_{36}H_{44}FeP_2$	1.03	594.54	Sigma-Aldrich	88723
L5	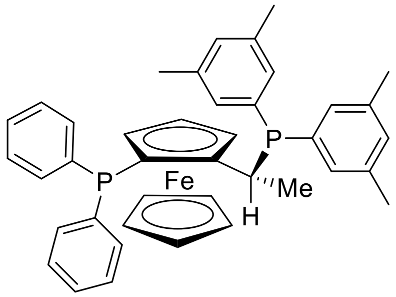	SL-J005-1	184095-69-0	PP	$C_{40}H_{40}FeP_2$	1.03	638.55	Sigma-Aldrich	88725
L6	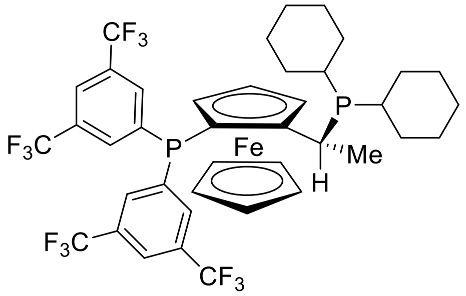	SL-J006-1	292638-88-1	PP	$C_{40}H_{40}F_{12}FeP_2$	1.06	866.53	Sigma-Aldrich	88727

#	Structure	Alias or name	CAS#	Type	Formula	Eq. to Rh	M_w [g mol^{-1}]	Vendor	Catalog #
L7	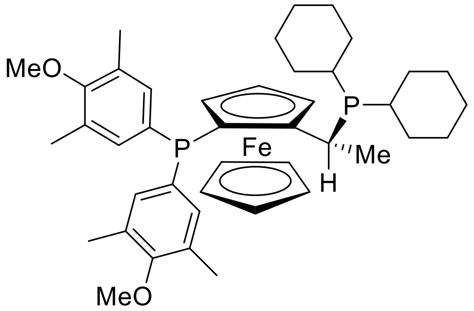	SL-J007-1	360048-63-1	PP	$C_{42}H_{56}FeO_2P_2$	1.00	710.70	Sigma-Aldrich	88729
L8	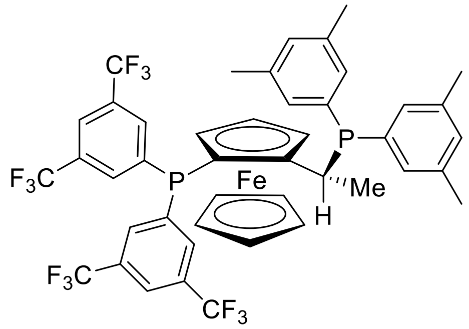	SL-J008-1	166172-63-0	PP	$C_{44}H_{36}F_{12}FeP_2$	1.03	910.55	Sigma-Aldrich	88731
L9	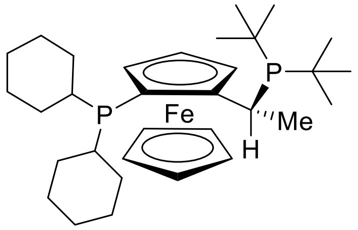	SL-J009-1	158923-11-6	PP	$C_{32}H_{52}FeP_2$	1.03	554.56	Sigma-Aldrich	88733
L10	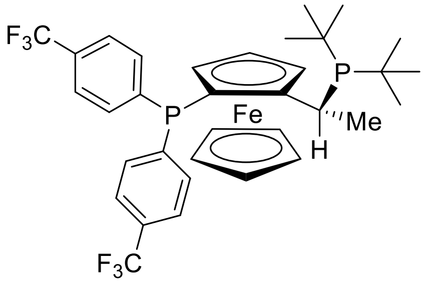	SL-J011-1	246231-79-8	PP	$C_{34}H_{38}F_6FeP_2$	1.04	678.46	Sigma-Aldrich	88735
L11	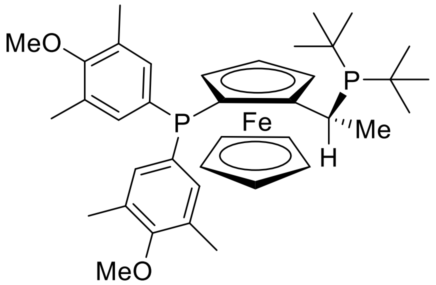	SL-J013-1	187733-50-2	PP	$C_{38}H_{52}FeO_2P_2$	1.03	658.62	Sigma-Aldrich	88737
L12	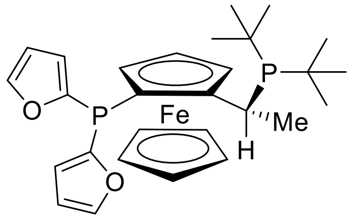	SL-J212-1	849924-41-0	PP	$C_{28}H_{36}FeO_2P_2$	1.08	522.39	abcr	AB426473
L13	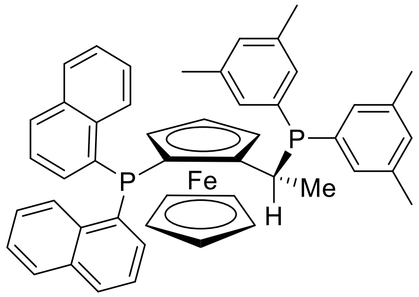	SL-J404-1	851308-40-2	PP	$C_{48}H_{44}FeP_2$	1.03	738.67	STREM	26-1175

#	Structure	Alias or name	CAS#	Type	Formula	Eq. to Rh	M_w [g mol^{-1}]	Vendor	Catalog #
L14	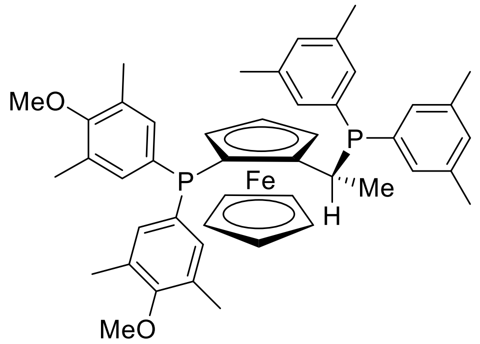	SL-J418-1	849924-45-4	PP	$C_{46}H_{52}FeO_2P_2$	1.03	754.71	Sigma-Aldrich	88747
L15	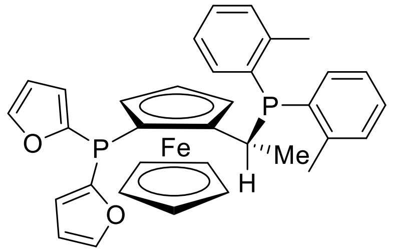	SL-J452-1	849924-73-8	PP	$C_{34}H_{32}FeO_2P_2$	1.05	590.42	Sigma-Aldrich	88751
L16	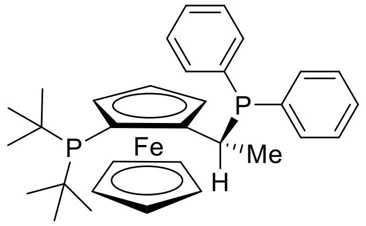	SL-J502-1	223120-71-6	PP	$C_{32}H_{40}FeP_2$	1.06	542.46	Sigma-Aldrich	88753
L17	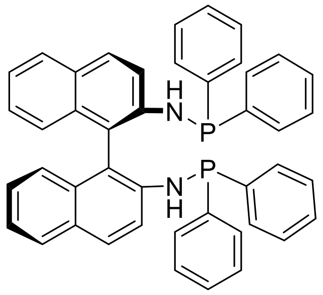	(*R*)-BINAM-P	74974-14-4	PP	$C_{44}H_{34}N_2P_2$	1.02	652.72	Sigma-Aldrich	708615
L18	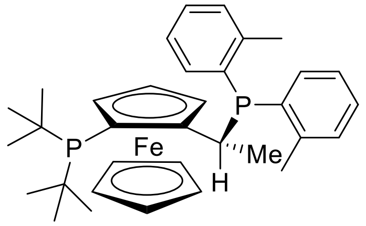	SL-J505-1	849924-76-1	PP	$C_{34}H_{44}FeP_2$	1.07	570.52	Sigma-Aldrich	88755
L19	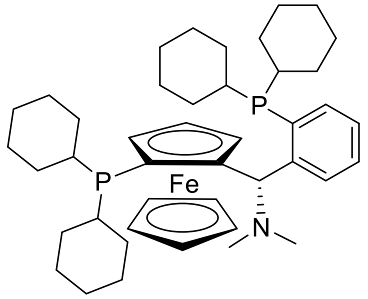	SL-T002-2	914089-00-2	PP	$C_{43}H_{63}FeNP_2$	1.01	711.78	Sigma-Aldrich	07542
L20	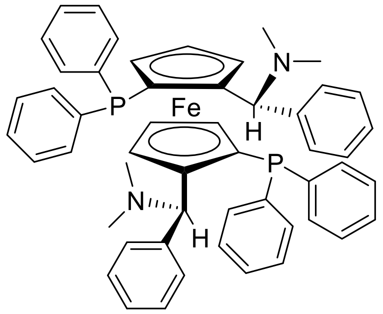	SL-M001-1	174467-31-3	PP	$C_{52}H_{50}FeN_2P_2$	1.03	820.78	Sigma-Aldrich	73463

#	Structure	Alias or name	CAS#	Type	Formula	Eq. to Rh	M_w [g mol^{-1}]	Vendor	Catalog #
L21	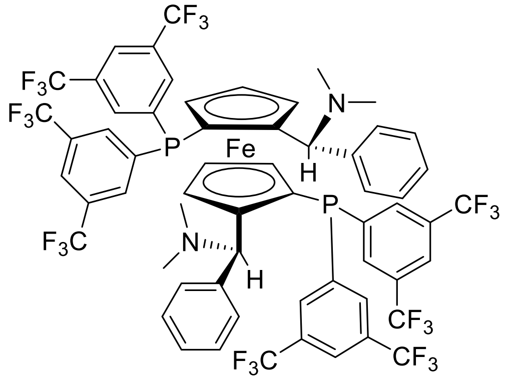	SL-M003-1	494227-36-0	PP	$C_{60}H_{42}F_{24}FeN_2P_2$	1.02	1364.76	Sigma-Aldrich	73467
L22	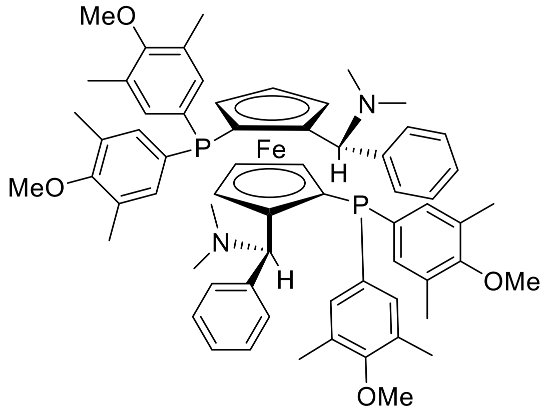	SL-M004-1	494227-37-1	PP	$C_{64}H_{74}FeN_2O_4P_2$	1.04	1053.10	Sigma-Aldrich	73469
L23	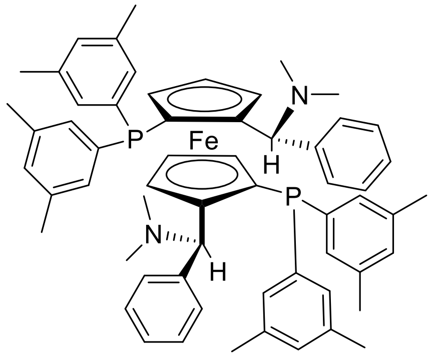	SL-M009-1	793718-16-8	PP	$C_{60}H_{66}FeN_2P_2$	1.01	932.99	Sigma-Aldrich	73471
L24	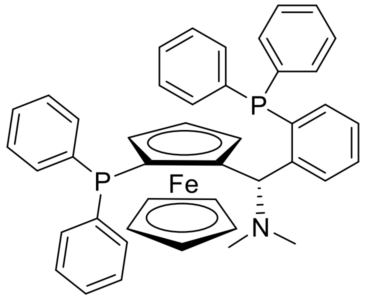	SL-T001-2	850444-36-9	PP	$C_{43}H_{39}FeNP_2$	1.03	687.58	Sigma-Aldrich	73476
L25	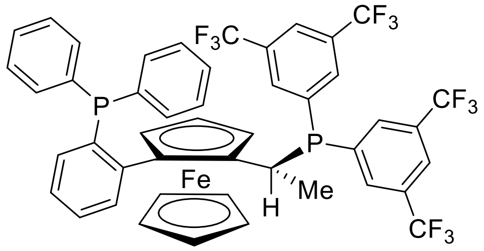	SL-W001-1	565184-33-0 (AKA 387868-06-6)	PP	$C_{46}H_{32}F_{12}FeP_2$	1.05	930.54	Sigma-Aldrich	65671
L26	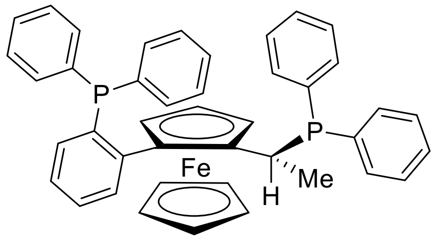	SL-W002-1	388079-58-1	PP	$C_{42}H_{36}FeP_2$	1.08	658.54	Sigma-Aldrich	65673

#	Structure	Alias or name	CAS#	Type	Formula	Eq. to Rh	M_w [g mol^{-1}]	Vendor	Catalog #
L27	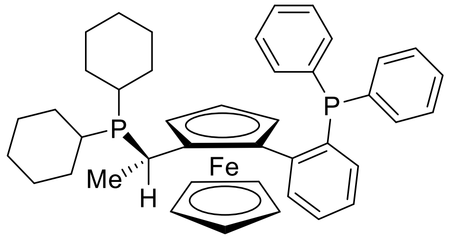	SL-W003-2	849925-19-5	PP	$C_{42}H_{48}FeP_2$	1.06	670.64	Sigma-Aldrich	65676
L28	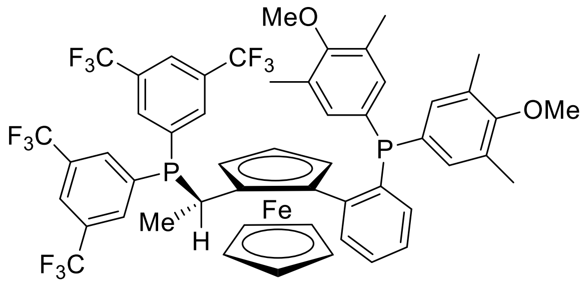	SL-W005-2	849925-20-8	PP	$C_{52}H_{44}F_{12}FeO_2P_2$	1.02	1046.70	Sigma-Aldrich	65678
L29	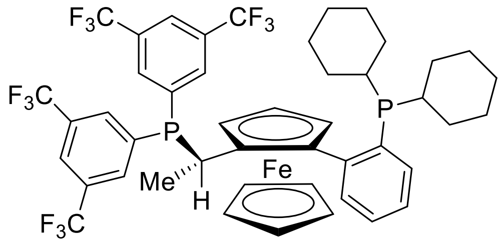	SL-W008-2	849925-22-0	PP	$C_{46}H_{44}F_{12}FeP_2$	1.07	942.63	Sigma-Aldrich	65682
L30	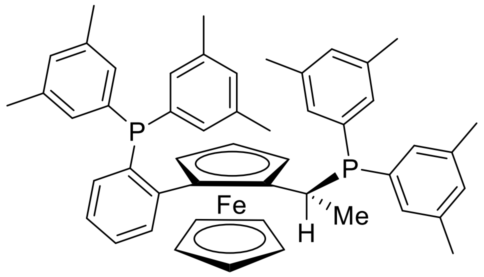	SL-W009-1	894771-28-9	PP	$C_{50}H_{52}FeP_2$	1.02	770.76	STREM	26-1555
L31	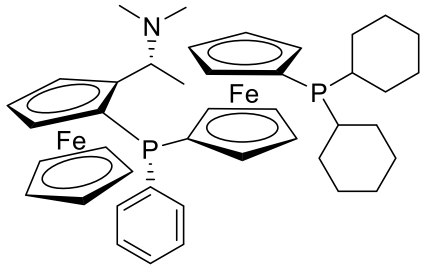	SL-F356-1	952586-19-5	PP	$C_{42}H_{53}Fe_2NP_2$	1.04	745.53	Sigma-Aldrich	779075
L32	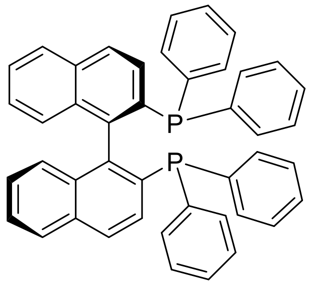	(*R*)-BINAP	76189-55-4	PP	$C_{44}H_{32}P_2$	1.09	622.69	Sigma-Aldrich	693065

#	Structure	Alias or name	CAS#	Type	Formula	Eq. to Rh	M_w [g mol^{-1}]	Vendor	Catalog #
L33	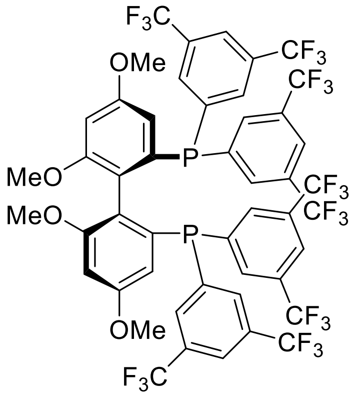	(*R*)-BTFM-GarPhos	1365531-84-5	PP	$C_{48}H_{28}F_{24}O_4P_2$	1.07	1186.66	STREM	15-1663
L34	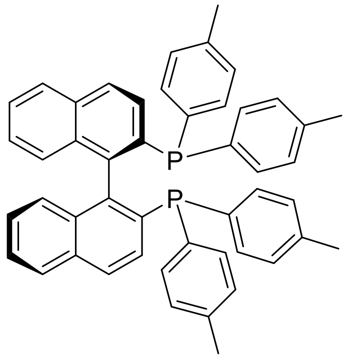	(*R*)-Tol-BINAP	99646-28-3	PP	$C_{48}H_{40}P_2$	1.04	678.80	Sigma-Aldrich	693049
L35	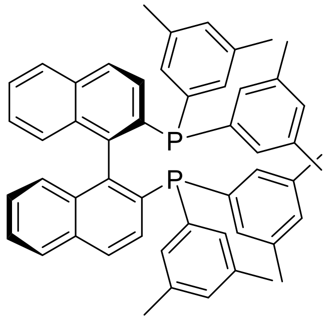	(*R*)-Xyl-BINAP<br>(AKA (*R*)-DM-BINAP)	137219-86-4	PP	$C_{52}H_{48}P_2$	1.05	734.90	Sigma-Aldrich	692379
L36	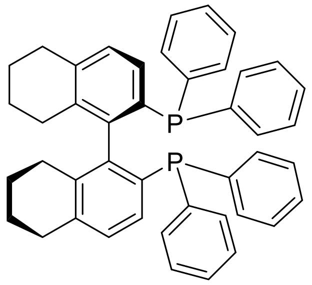	(*R*)-H_8-BINAP	139139-86-9	PP	$C_{44}H_{40}P_2$	1.09	630.75	Sigma-Aldrich	692387
L37	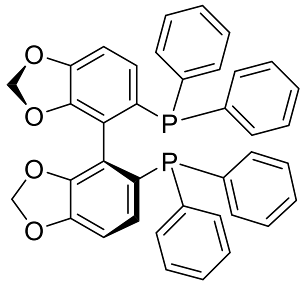	(*S*)-SegPhos	210169-54-3	PP	$C_{38}H_{28}O_4P_2$	1.09	610.59	Sigma-Aldrich	693006

#	Structure	Alias or name	CAS#	Type	Formula	Eq. to Rh	M_w [g mol^{-1}]	Vendor	Catalog #
L38	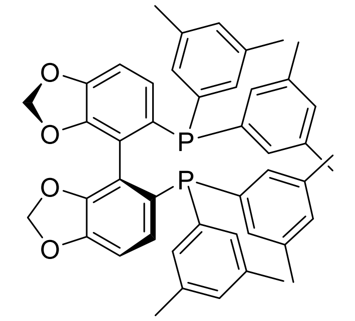	(*S*)-Xyl-SegPhos<br>(AKA (*S*)-DM-SegPhos)	210169-57-6	PP	$C_{46}H_{44}O_4P_2$	1.05	722.80	Sigma-Aldrich	692999
L39	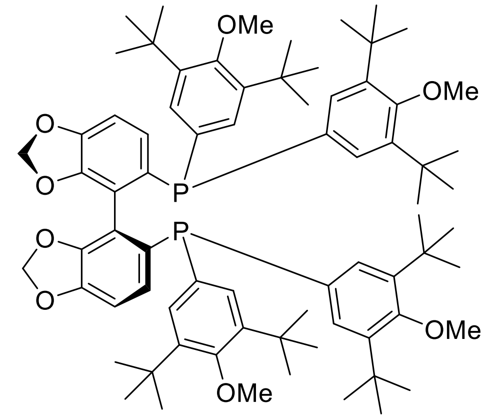	(*S*)-DTBM-SegPhos	210169-40-7	PP	$C_{74}H_{100}O_8P_2$	1.09	1179.55	Sigma-Aldrich	692980
L40	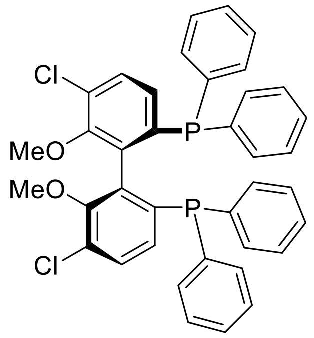	(*R*)-Cl-MeO-BIPHEP	185913-97-7	PP	$C_{38}H_{30}Cl_2O_2P_2$	1.00	651.50	Sigma-Aldrich	96738
L41	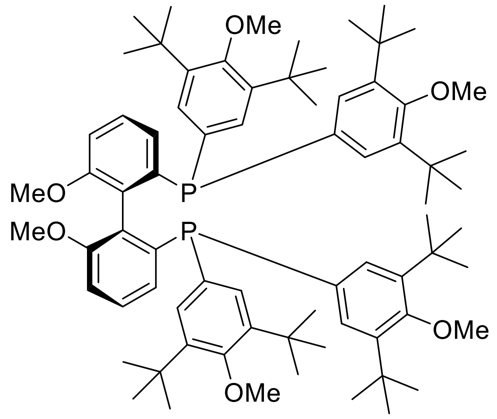	SL-A109-1	352655-61-9	PP	$C_{74}H_{104}O_6P_2$	1.01	1151.59	Sigma-Aldrich	29512
L42	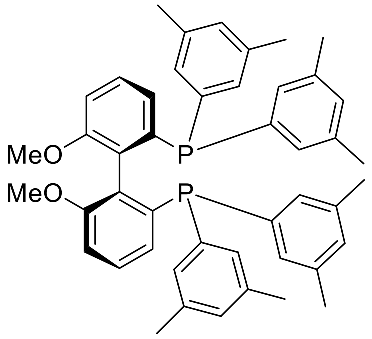	SL-A120-1	394248-45-4	PP	$C_{46}H_{48}O_2P_2$	1.07	694.84	Sigma-Aldrich	29516

#	Structure	Alias or name	CAS#	Type	Formula	Eq. to Rh	M_w [g mol^{-1}]	Vendor	Catalog #
L43	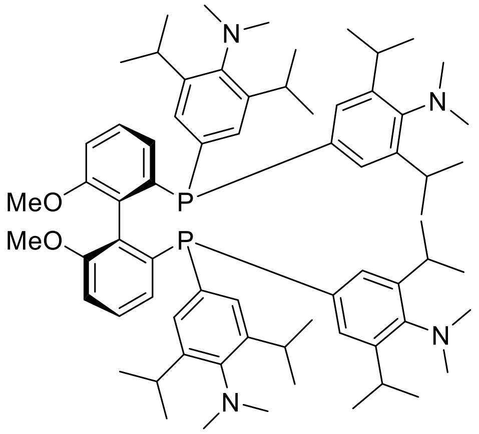	SL-A107-1	352655-40-4	PP	$C_{70}H_{100}N_4O_2P_2$	1.02	1091.54	Sigma-Aldrich	29528
L44	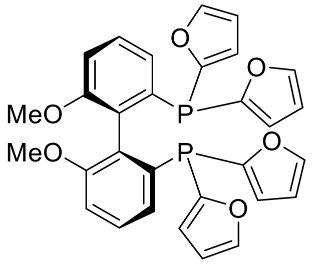	SL-A108-2	145214-59-1	PP	$C_{30}H_{24}O_6P_2$	1.08	542.46	Sigma-Aldrich	29515
L45	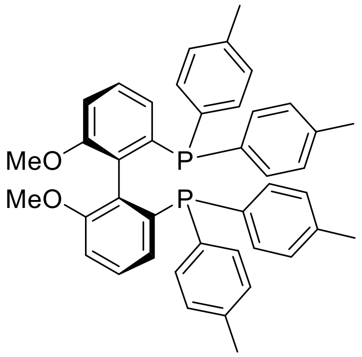	SL-A102-2	133545-25-2	PP	$C_{42}H_{40}O_2P_2$	1.01	638.73	Sigma-Aldrich	29521
L46	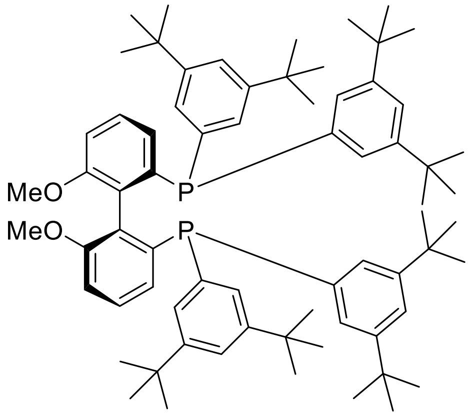	SL-A121-1	192138-05-9	PP	$C_{70}H_{96}O_2P_2$	1.10	1031.48	Sigma-Aldrich	29524
L47	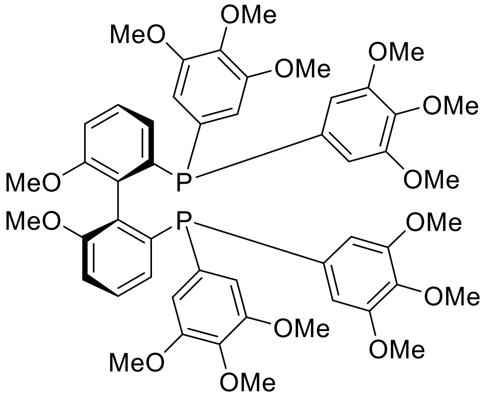	SL-A104-1	256390-47-3	PP	$C_{50}H_{56}O_{14}P_2$	1.05	942.93	Sigma-Aldrich	29526

#	Structure	Alias or name	CAS#	Type	Formula	Eq. to Rh	M_w [g mol^{-1}]	Vendor	Catalog #
L48	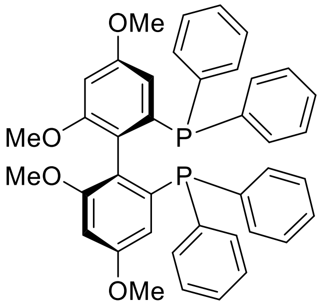	(*R*)-GarPhos	1365531-75-4	PP	$C_{40}H_{36}O_4P_2$	1.06	642.67	Sigma-Aldrich	754927
L49	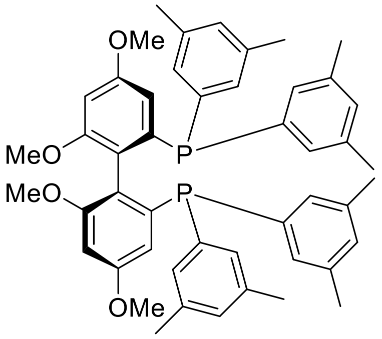	(*R*)-Xyl-GarPhos	1365531-89-0	PP	$C_{48}H_{52}O_4P_2$	1.00	754.89	Sigma-Aldrich	761389
L50	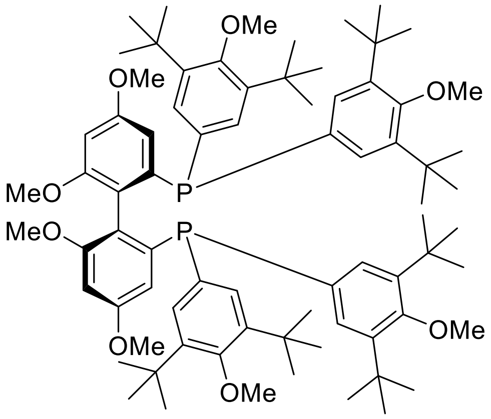	(*R*)-DTBM-GarPhos	1365531-98-1	PP	$C_{76}H_{108}O_8P_2$	1.03	1211.64	Sigma-Aldrich	761419
L51	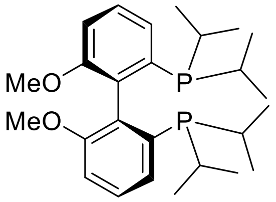	(*S*)-^{i}Pr-BIPHEP	150971-43-0	PP	$C_{26}H_{40}O_2P_2$	1.03	446.55	STREM	15-0655
L52	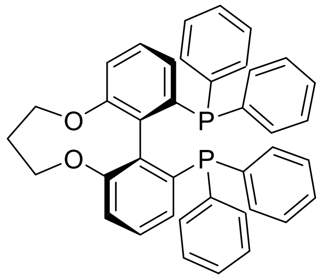	(*R*)-C$_3$-TunePhos	301847-89-2	PP	$C_{39}H_{32}O_2P_2$	1.05	594.63	STREM	15-0175
L53	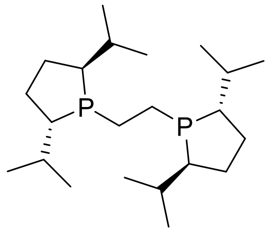	(*S*,*S*)-^{i}Pr-BPE	528854-34-4	PP	$C_{22}H_{44}P_2$	1.07	370.54	Sigma-Aldrich	668435

#	Structure	Alias or name	CAS#	Type	Formula	Eq. to Rh	M_w [g mol^{-1}]	Vendor	Catalog #
L54	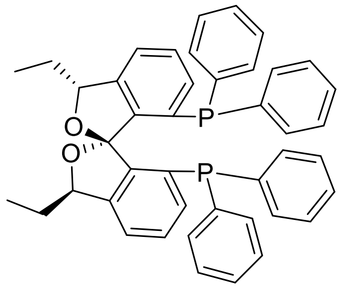	(*R*,*R*,*R*)-SPIRAP<br>(AKA (*R*)-CrabPhos)	NA	PP	$C_{43}H_{38}O_2P_2$	1.03	648.72	Sigma-Aldrich	905240
L55	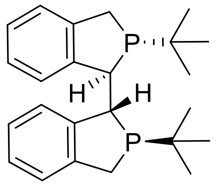	(*R*,*R*,*S*,*S*)-DuanPhos	528814-26-8	PP	$C_{24}H_{32}P_2$	1.09	382.46	Sigma-Aldrich	657697
L56	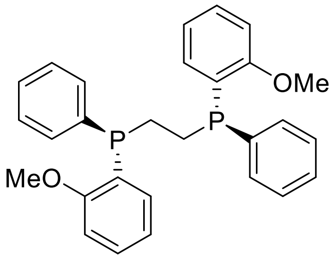	(*R*,*R*)-DIPAMP	55739-58-7	PP	$C_{28}H_{28}O_2P_2$	1.06	458.48	Sigma-Aldrich	697761
L57	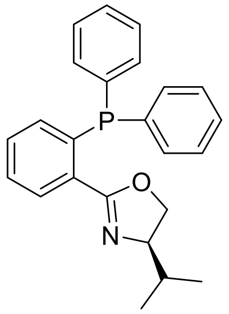	(*R*)-^{i}Pr-PHOX	164858-78-0	PN	$C_{24}H_{24}NOP$	1.09	373.44	Sigma-Aldrich	72575
L58	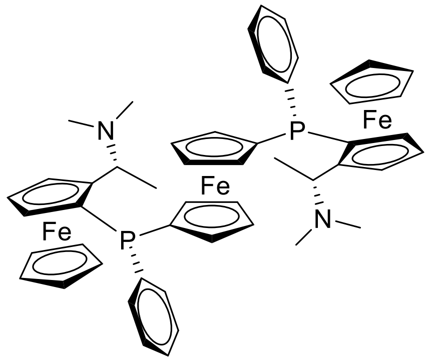	SL-F131-1<br>(AKA Trifer)	899811-43-9	PP	$C_{50}H_{54}Fe_3N_2P_2$	1.03	912.48	STREM	26-1260
L59	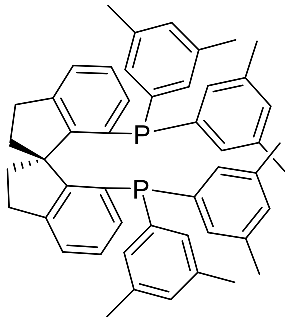	(*R*)-Xyl-SDP	917377-75-4	PP	$C_{49}H_{50}P_2$	1.04	700.89	STREM	15-5168
L60	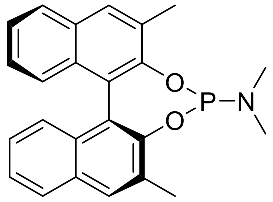	(*S*)-DM-MonoPhos	185449-86-9	Phosphoramidite	$C_{24}H_{22}NO_2P$	2.00	387.42	STREM	15-1255

#	Structure	Alias or name	CAS#	Type	Formula	Eq. to Rh	M_w [g mol^{-1}]	Vendor	Catalog #
L61	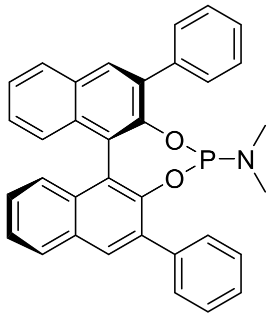	(*R*)-Ph-MonoPhos	936010-61-6	Phosphoramidite	$C_{34}H_{26}NO_2P$	2.00	511.56	STREM	15-5620
L62	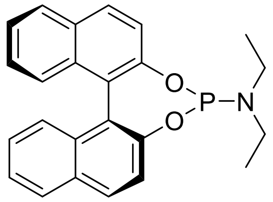	(*S*)-NEt$_2$-MonoPhos	252288-04-3	Phosphoramidite	$C_{24}H_{22}NO_2P$	2.00	387.42	STREM	15-1231
L63	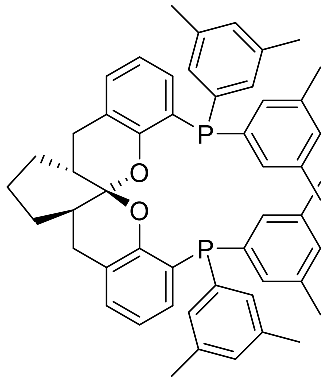	(*R*,*R*,*R*)-Xyl-SKP	1429939-35-4	PP	$C_{52}H_{54}O_2P_2$	1.03	772.95	STREM	15-4320
L64	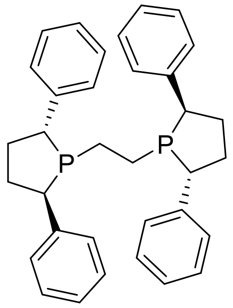	(*R*,*R*)-Ph-BPE	528565-79-9	PP	$C_{34}H_{36}P_2$	1.09	506.61	Sigma-Aldrich	667811
L65	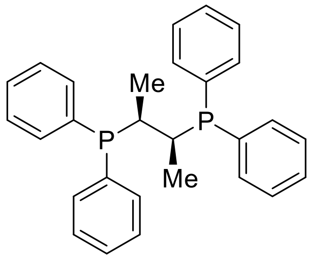	(*S*,*S*)-ChiraPhos	64896-28-2	PP	$C_{28}H_{28}P_2$	1.04	426.48	Sigma-Aldrich	259098
L66	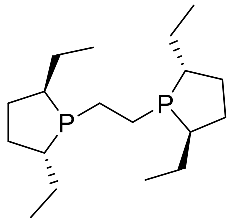	(*R*,*R*)-Et-BPE	136705-62-9	PP	$C_{18}H_{36}P_2$	1.06	314.43	Sigma-Aldrich	668478
L67	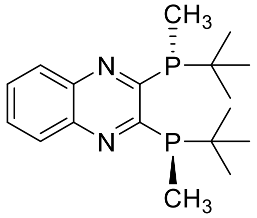	(*R*)-QuinoxP	866081-62-1	PP	$C_{18}H_{28}N_2P_2$	1.06	334.38	Sigma-Aldrich	676403

#	Structure	Alias or name	CAS#	Type	Formula	Eq. to Rh	M_w [g mol^{-1}]	Vendor	Catalog #
L68	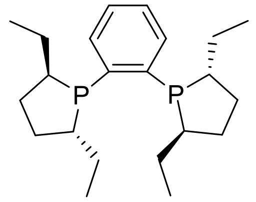	(*R*,*R*)-Et-DuPhos	136705-64-1	PP	$C_{22}H_{36}P_2$	1.04	362.48	Sigma-Aldrich	668494
L69	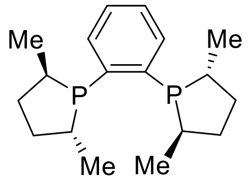	(*R*,*R*)-Me-DuPhos	147253-67-6	PP	$C_{18}H_{28}P_2$	1.08	306.37	Sigma-Aldrich	665258
L70	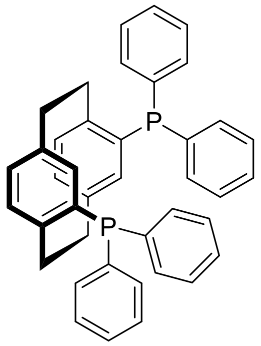	(*S*)-PhanePhos	192463-40-4	PP	$C_{40}H_{34}P_2$	1.09	576.66	Sigma-Aldrich	682136
L71	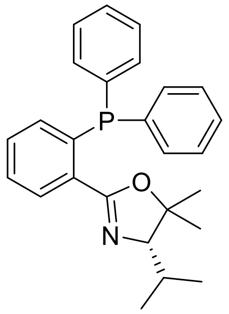	(*S*)-Me-^{i}Pr-PHOX	1152313-76-2	PN	$C_{26}H_{28}NOP$	1.05	401.49	Sigma-Aldrich	719641
L72	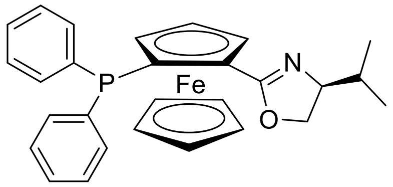	SL-N003-2	163169-29-7	PN	$C_{28}H_{28}FeNOP$	1.09	481.36	Sigma-Aldrich	717398
L73	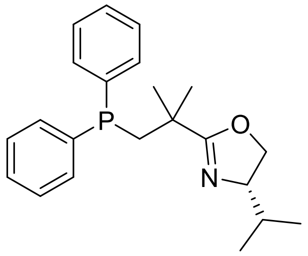	(*S*)-NeoPHOX	1199225-38-1	PN	$C_{22}H_{28}NOP$	1.05	353.45	Sigma-Aldrich	729264
L74	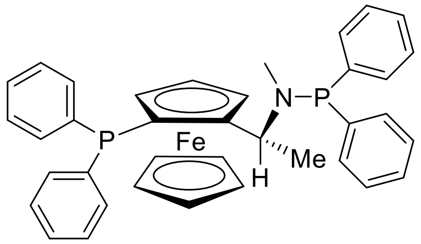	(*R*,*S*)-Me-BoPhoz	406680-94-2	PP	$C_{37}H_{35}FeNP_2$	1.06	611.49	Sigma-Aldrich	682322

#	Structure	Alias or name	CAS#	Type	Formula	Eq. to Rh	M_w [g mol^{-1}]	Vendor	Catalog #
L75	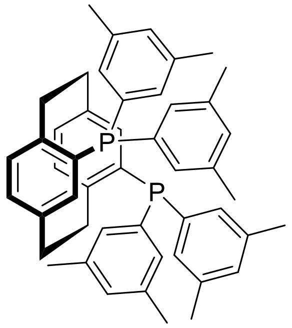	(*R*)-Xyl-PhanePhos	325168-89-6	PP	$C_{48}H_{50}P_2$	1.08	688.88	Sigma-Aldrich	682306
L76	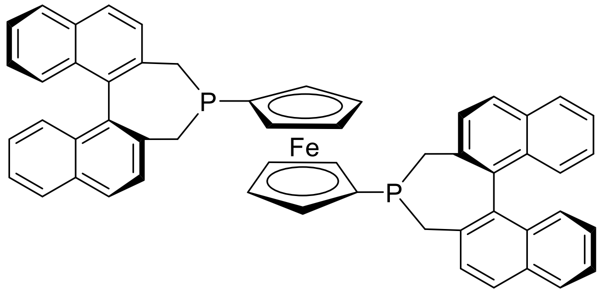	(*S*,*S*)-f-Binaphane	544461-38-3	PP	$C_{54}H_{40}FeP_2$	1.09	806.71	STREM	26-0243
L77	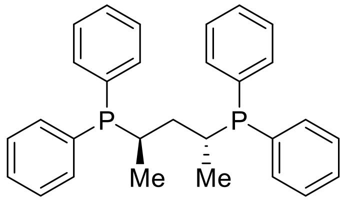	(*R*,*R*)-BDPP	96183-46-9	PP	$C_{29}H_{30}P_2$	1.09	440.51	STREM	15-0432
L78	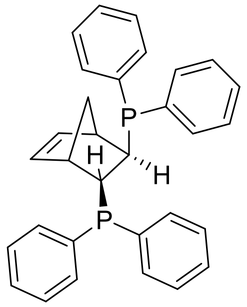	(*R*,*R*)-NorPhos	71042-55-2	PP	$C_{31}H_{28}P_2$	1.09	462.51	STREM	15-0140
L79	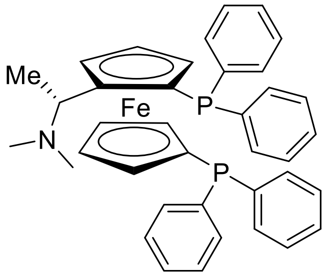	(*R*,*S*)-BPPFA	74311-56-1	PP	$C_{38}H_{37}FeNP_2$	1.06	625.51	Sigma-Aldrich	344087
L80	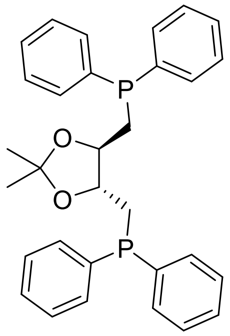	(*R*,*R*)-DIOP	32305-98-9	PP	$C_{31}H_{32}O_2P_2$	1.07	498.54	Sigma-Aldrich	237655

#	Structure	Alias or name	CAS#	Type	Formula	Eq. to Rh	M_w [g mol^{-1}]	Vendor	Catalog #
L81	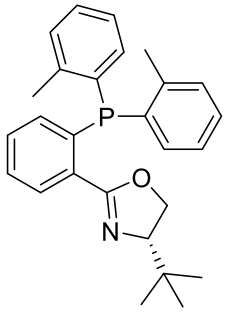	(*S*)-Tol-^{t}Bu-PHOX	218460-00-5	PN	C27H30NOP	1.10	415.52	Sigma-Aldrich	688533
L82	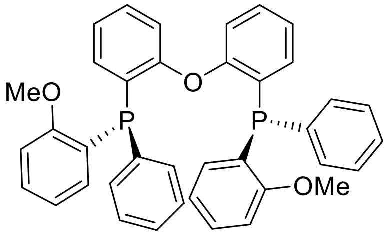	(*S*,*S*)-DPE-Phos	2119686-55-2	PP	$C_{38}H_{32}O_3P_2$	1.06	598.62	STREM	15-1279
L83	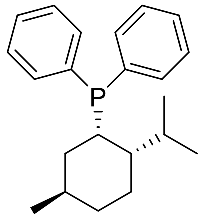	(*S*)-NMDPP	43077-29-8	P	$C_{22}H_{29}P$	2.00	324.45	STREM	15-3490
L84	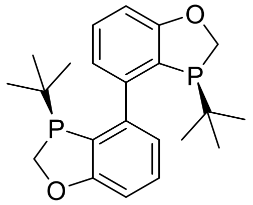	(*S*,*S*)-BABIBOP	2207601-04-3	PP	$C_{22}H_{28}O_2P_2$	1.03	386.41	STREM	15-6410
L85	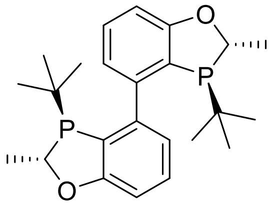	(*S*,*S*,*S*,*S*)-Me-BABIBOP	2207601-10-1	PP	$C_{24}H_{32}O_2P_2$	1.06	414.47	STREM	15-6420
L86	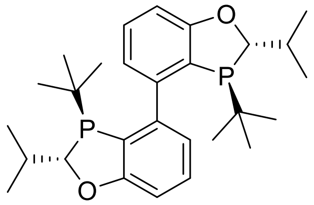	(*S*,*S*,*S*,*S*)-^{i}Pr-BABIBOP	2207601-12-3	PP	$C_{28}H_{40}O_2P_2$	1.08	470.57	STREM	15-6430
L87	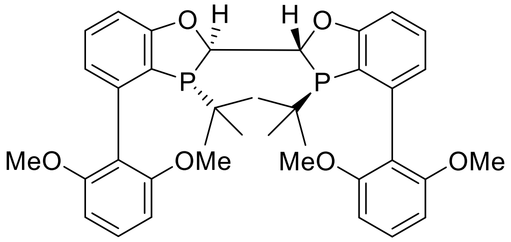	(*R*,*R*,*R*,*R*)-Bis-BIDIME	1884680-48-1	PP	$C_{38}H_{44}O_6P_2$	1.03	658.71	STREM	15-6240
L88	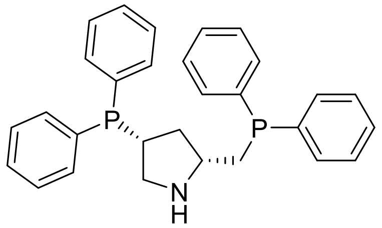	(*R*,*R*)-PPM	77450-05-6	PP	$C_{29}H_{29}NP_2$	1.05	453.51	STREM	15-7210

#	Structure	Alias or name	CAS#	Type	Formula	Eq. to Rh	M_w [g mol^{-1}]	Vendor	Catalog #
L89	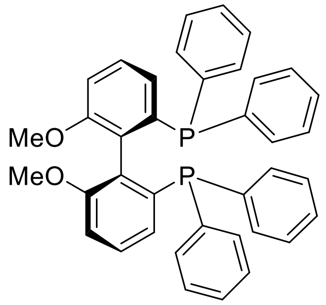	SL-A101-1	133545-16-1	PP	$C_{38}H_{32}O_2P_2$	1.03	582.62	Sigma-Aldrich	29510
L90	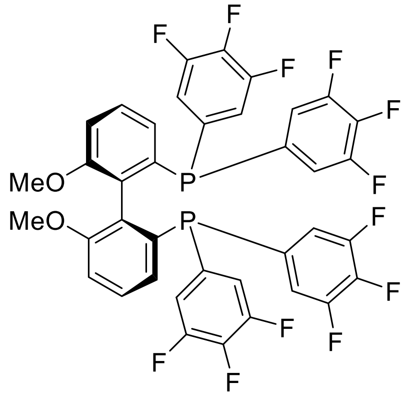	(*S*)-MeO-F$_{12}$-BIPHEP	116008-37-6	PP	$C_{38}H_{20}F_{12}O_2P_2$	1.06	798.50	Kanto	25971-95
L91	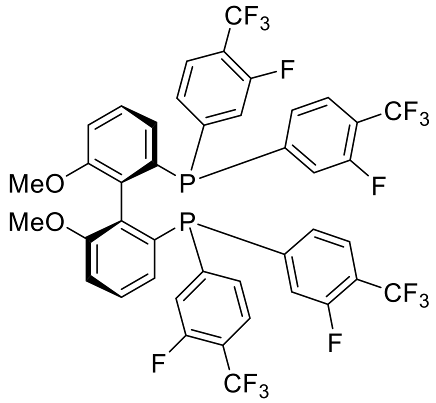	(*R*)-MeO-F$_{16}$-BIPHEP	NA	PP	$C_{42}H_{24}F_{16}O_2P_2$	1.03	926.57	Kanto	2022-008K
L92	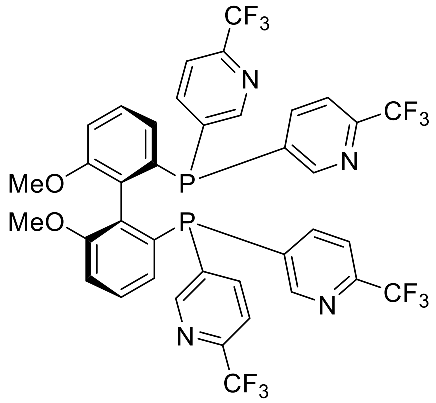	(*R*)-MeO-Py-F$_{12}$-BIPHEP	NA	PP	$C_{38}H_{24}F_{12}N_4O_2P_2$	1.06	858.56	Kanto	2022-009K
L93	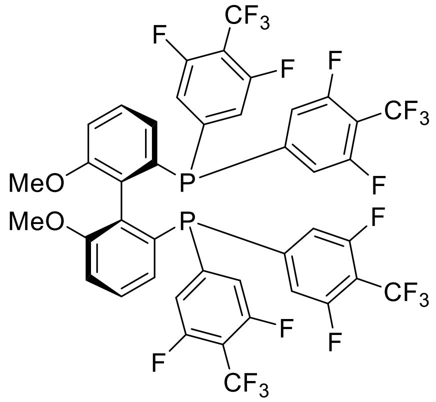	(*R*)-MeO-F$_{20}$-BIPHEP	NA	PP	$C_{42}H_{20}F_{20}O_2P_2$	1.02	998.54	Kanto	2022-010K

#	Structure	Alias or name	CAS#	Type	Formula	Eq. to Rh	M_w [g mol^{-1}]	Vendor	Catalog #
L94	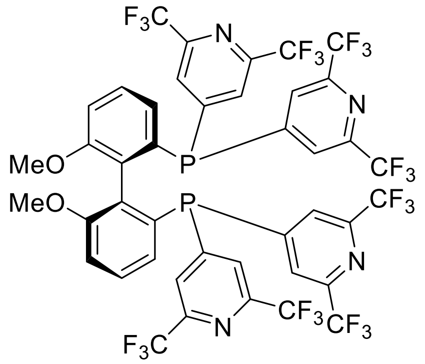	(*R*)-MeO-BFPy-BIPHEP	NA	PP	$C_{42}H_{20}F_{24}N_4O_2P_2$	1.03	1130.56	Kanto	2022-011K
L95	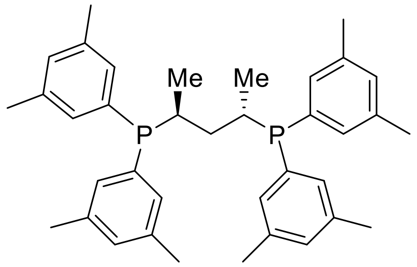	(*S*,*S*)-XylSKEWPhos	551950-92-6	PP	$C_{37}H_{46}P_2$	1.08	552.72	Kanto	05843-68
L96	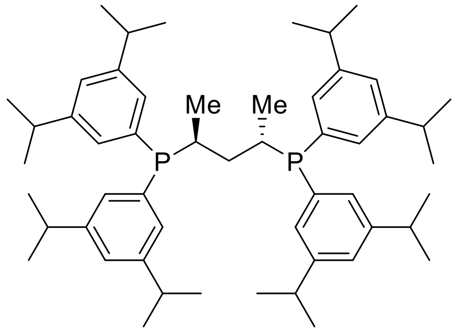	(*S*,*S*)-DIPSKEWPhos	NA	PP	$C_{53}H_{78}P_2$	1.10	777.15	Kanto	05845-55
L97	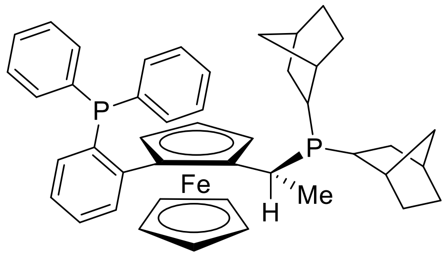	SL-W022-1	849925-29-7	PP	$C_{44}H_{48}FeP_2$	1.08	694.66	Santa Cruz Biotechnology	sc-237419
L98	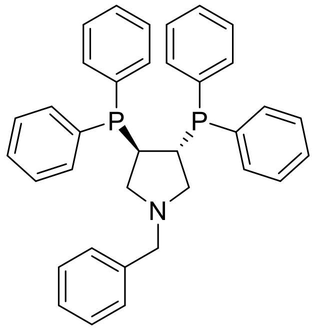	catASium D(*R*)	99135-95-2	PP	$C_{35}H_{33}NP_2$	1.02	529.60	abcr	AB152827
L99	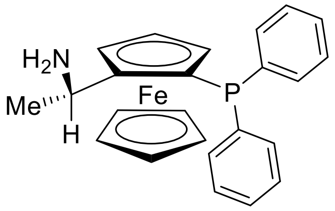	(2*R*)-1-[(1*S*)-1-Aminoethyl]-2-(diphenylphosphino)ferrocene	607389-84-4	PN	$C_{24}H_{24}FeNP$	1.07	413.28	abcr	AB268697

#	Structure	Alias or name	CAS#	Type	Formula	Eq. to Rh	M$_w$ [g mol^{-1}]	Vendor	Catalog #
L100	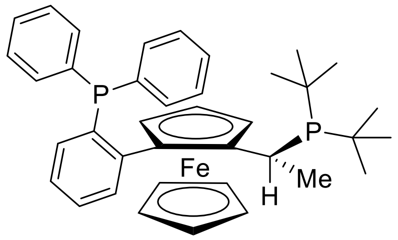	SL-W012-1	565184-30-7	PP	$C_{38}H_{44}FeP_2$	1.06	618.56	abcr	AB426507
L101	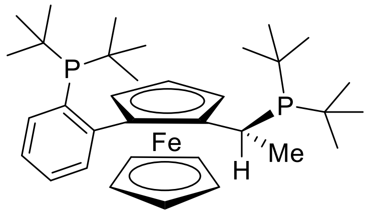	SL-W030-1	1854067-62-1	PP	$C_{34}H_{52}FeP_2$	1.03	578.58	abcr	AB426511
L102	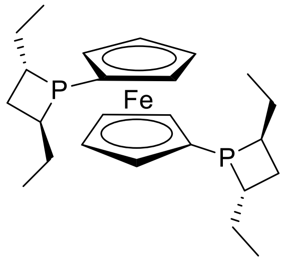	(*S*,*S*)-Et-FerroTANE	290347-66-9	PP	$C_{24}H_{36}FeP_2$	1.09	442.34	abcr	AB131607
L103	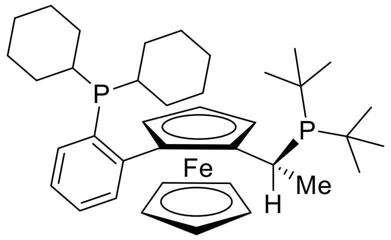	SL-W029-1	1854067-50-7	PP	$C_{38}H_{56}FeP_2$	1.06	630.66	abcr	AB426509
L104	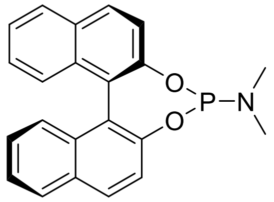	(*R*)-MonoPhos	157488-65-8	Phosphoramidite	$C_{22}H_{18}NO_2P$	2.02	359.36	Sigma-Aldrich	668206
L105	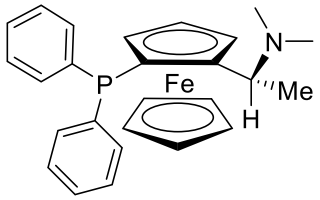	SL-F103-1	55700-44-2	PN	$C_{26}H_{28}FeNP$	1.05	441.34	abcr	AB180833
L106	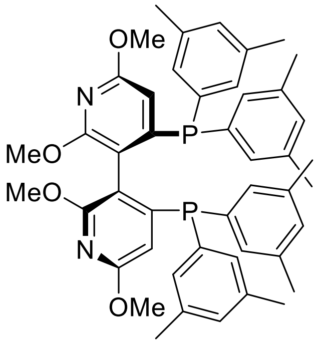	(*R*)-Xyl-P-Phos	442905-33-1	PP	$C_{46}H_{50}N_2O_4P_2$	1.06	756.86	STREM	15-5210
L107	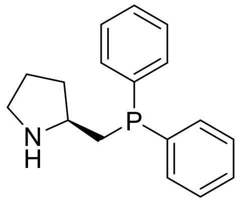	(*S*)-2-(Diphenylphosphinomethyl)pyrrolidine	60261-46-3	PN	$C_{17}H_{20}NP$	1.06	269.33	Sigma-Aldrich	716715

#	Structure	Alias or name	CAS#	Type	Formula	Eq. to Rh	M_w [g mol^{-1}]	Vendor	Catalog #
L108	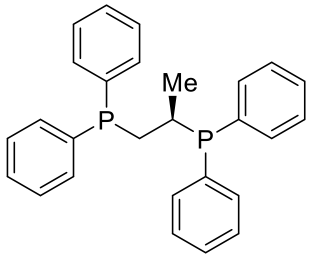	(*R*)-ProPhos	67884-32-6	PP	$C_{27}H_{26}P_2$	1.06	412.45	STREM	15-0440
L109	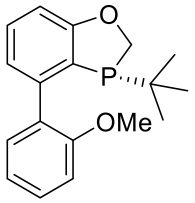	(3*R*)-3-(1,1-Dimethylethyl)-2,3-dihydro-4-(2-methoxyphenyl)-1,3-benzoxaphosphole	1338454-28-6	P	$C_{18}H_{21}O_2P$	2.04	300.34	STREM	15-6872
L110	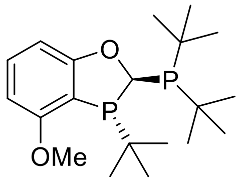	(2*S*,3*R*)-MeO-POP	1215081-28-9	PP	$C_{20}H_{34}O_2P_2$	1.04	368.44	STREM	15-6280
L111	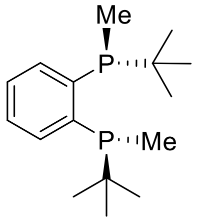	(*R*,*R*)-BenzP*	919778-41-9	PP	$C_{16}H_{28}P_2$	1.08	282.35	STREM	15-0166
L112	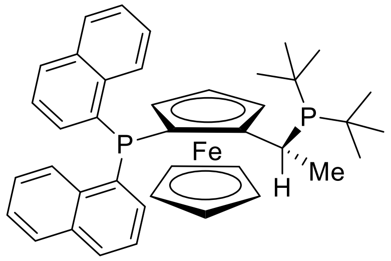	SL-J216-1	849924-43-2	PP	$C_{40}H_{44}FeP_2$	1.04	642.58	abcr	AB426475
L113	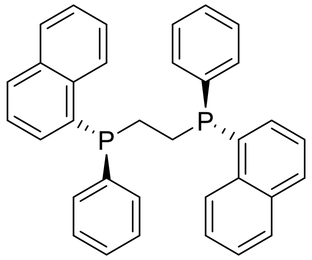	(*S*,*S*)-1-Naphthyl-DIPAMP	256469-70-2	PP	$C_{34}H_{28}P_2$	1.00	498.55	Sigma-Aldrich	697788
L114	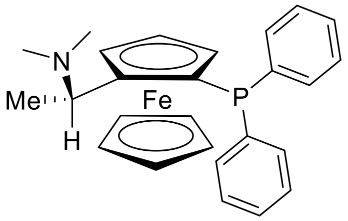	(*S*,*R*)-PPFA	55650-58-3	PN	$C_{26}H_{28}FeNP$	1.04	441.34	abcr	AB180834
L115	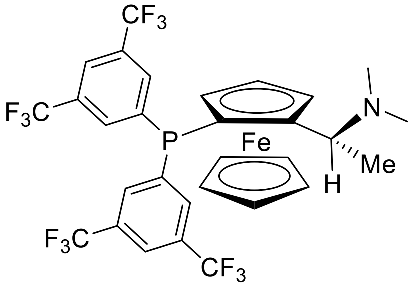	SL-F173-1	166172-70-9	PN	$C_{30}H_{24}F_{12}FeNP$	1.03	713.33	Sinocompound	SC-4479

#	Structure	Alias or name	CAS#	Type	Formula	Eq. to Rh	M_w [g mol^{-1}]	Vendor	Catalog #
L116	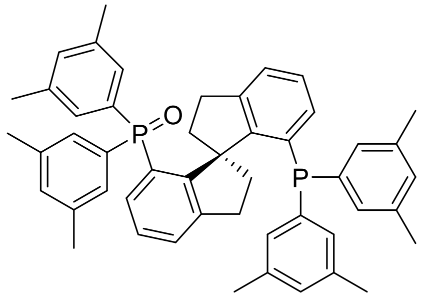	(*R*)-Xyl-SDP Oxide	1462321-89-6	P(P=O)	$C_{49}H_{50}OP_2$	1.04	716.89	Sigma-Aldrich	798711
L117	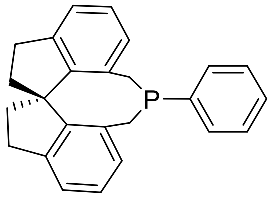	(*R*)-SITCP	856407-37-9	P	$C_{25}H_{23}P$	2.07	354.43	STREM	15-5184
L118	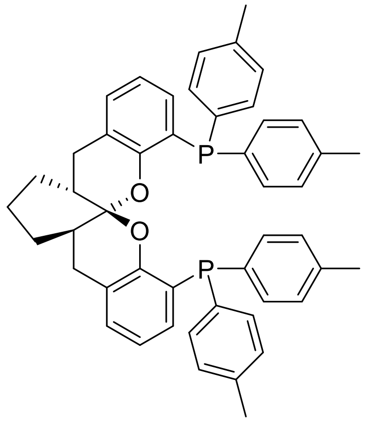	(*R*,*R*,*R*)-Tol-SKP	1429939-32-1	PP	$C_{48}H_{46}O_2P_2$	1.01	716.84	STREM	15-4330
L119	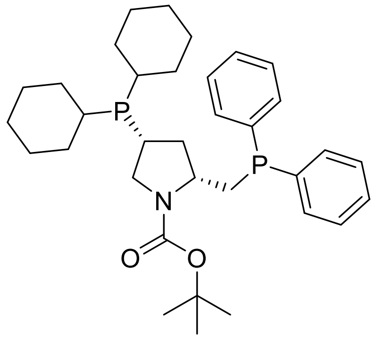	(*R*,*R*)-BCPM	114751-47-2	PP	$C_{34}H_{49}NO_2P_2$	1.06	565.72	STREM	15-7220
L120	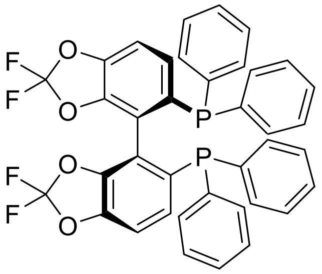	(*R*)-DiFluorPhos	503538-69-0	PP	$C_{38}H_{24}F_4O_4P_2$	1.04	682.55	STREM	15-0486
L121	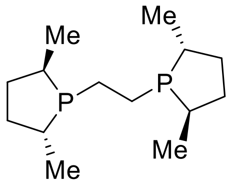	(*R*,*R*)-Me-BPE	129648-07-3	PP	$C_{14}H_{28}P_2$	1.10	258.33	STREM	15-0104

#	Structure	Alias or name	CAS#	Type	Formula	Eq. to Rh	M_w [g mol^{-1}]	Vendor	Catalog #
L122	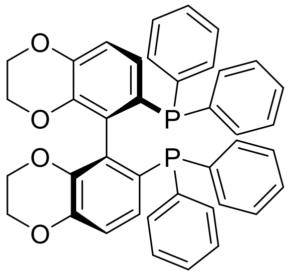	(*R*)-SynPhos	445467-61-8	PP	$C_{40}H_{32}O_4P_2$	1.06	638.64	STREM	15-0490
L123	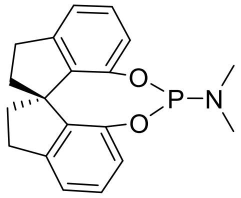	(*R*)-SIPhos	443965-14-8	Phosphoramidite	$C_{19}H_{20}NO_2P$	2.03	325.35	STREM	15-5150
L124	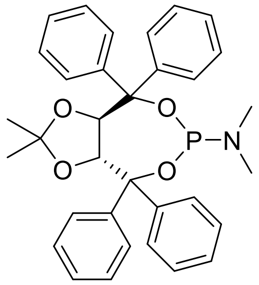	(3R,8R)-Tetrahydro-N,N,2,2-tetramethyl-4,4,8,8-tetraphenyl-1,3-dioxolo[4,5-e][1,3,2]dioxaphosphepin-6-amine	213843-90-4	Phosphoramidite	$C_{33}H_{34}NO_4P$	2.07	539.61	Sigma-Aldrich	665460
L125	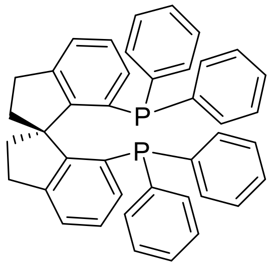	(*R*)-SDP	917377-74-3	PP	$C_{41}H_{34}P_2$	1.07	588.67	STREM	15-5174
L126	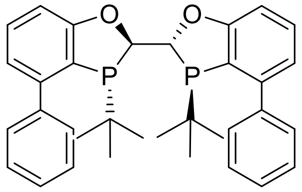	(*R*,*R*,*R*,*R*)-Ph-BIBOP	2301856-53-9	PP	$C_{34}H_{36}O_2P_2$	1.04	538.61	STREM	15-6260
L127	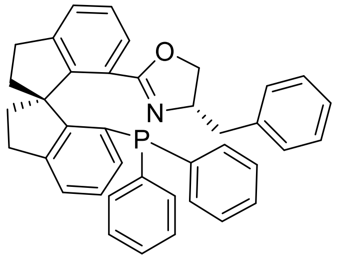	(*R*,*S*)-Ph-Bn-SIPHOX	2074610-05-0	PN	$C_{39}H_{34}NOP$	1.02	563.68	STREM	15-5186
L128	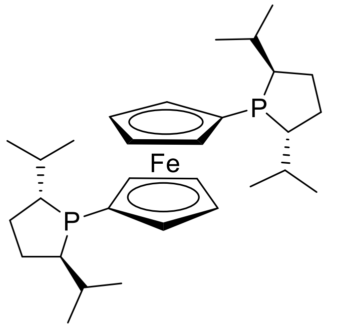	(*R*,*R*)-^{i}Pr-Ferrocelane<br>(AKA (*R*,*R*)-^{i}Pr-BPF)	849950-54-5	PP	$C_{30}H_{48}FeP_2$	1.02	526.51	STREM	26-1610

#	Structure	Alias or name	CAS#	Type	Formula	Eq. to Rh	M_w [g mol^{-1}]	Vendor	Catalog #
L129	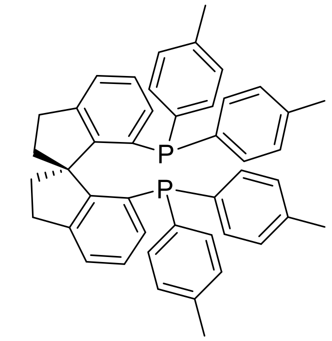	(*R*)-Tol-SDP	528521-87-1	PP	$C_{45}H_{42}P_2$	1.09	644.78	STREM	15-5180
L130	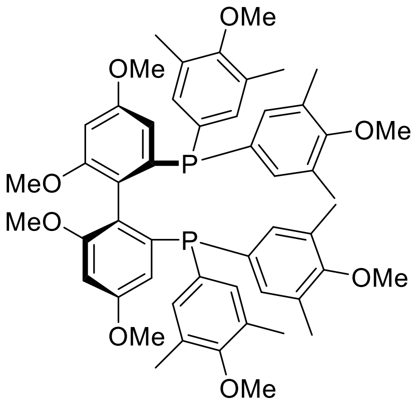	(*R*)-DMM-GarPhos	1365531-93-6	PP	$C_{52}H_{60}O_8P_2$	1.00	874.99	STREM	15-1666
L131	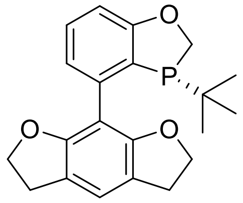	8-[(3*R*)-3-(1,1-Dimethylethyl)-2,3-dihydro-1,3-benzoxaphosphol-4-yl]benzo[1,2-b:5,4-b′]difuran	1835717-07-1	P	$C_{21}H_{23}O_3P$	2.03	354.39	STREM	15-6290
L132	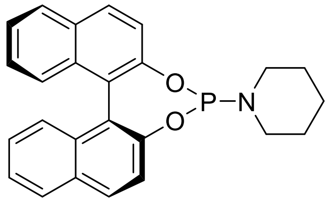	(*S*)-PipPhos	284472-79-3	Phosphoramidite	$C_{25}H_{22}NO_2P$	2.06	399.43	STREM	15-1234
L133	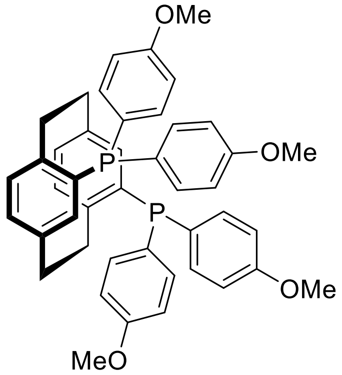	(*R*)-An-PhanePhos	364732-86-5	PP	$C_{44}H_{42}O_4P_2$	1.01	696.76	STREM	15-0710
L134	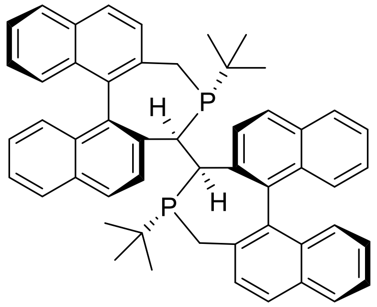	(*S*)-BINAPINE	528854-26-4	PP	$C_{52}H_{48}P_2$	1.00	734.90	STREM	15-1053

#	Structure	Alias or name	CAS#	Type	Formula	Eq. to Rh	M_w [g mol^{-1}]	Vendor	Catalog #
L135	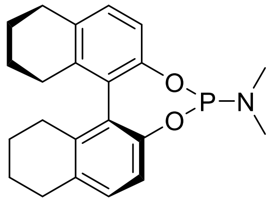	(*S*)-H8-MonoPhos	389130-06-7	Phosphoramidite	$C_{22}H_{26}NO_2P$	2.07	367.43	Sigma-Aldrich	685569
L136	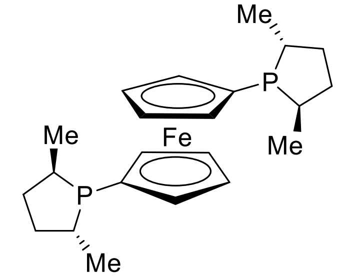	(*R*,*R*)-Me-Ferrocelane	540475-45-4	PP	$C_{22}H_{32}FeP_2$	1.03	414.29	Sigma-Aldrich	675601
L137	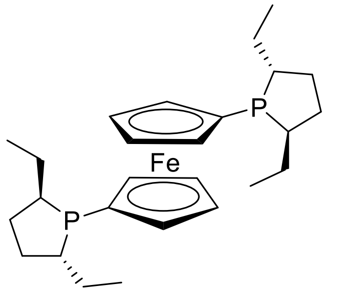	(*R*,*R*)-Et-Ferrocelane	147762-89-8	PP	$C_{26}H_{40}FeP_2$	1.05	470.40	Sigma-Aldrich	680990
L138	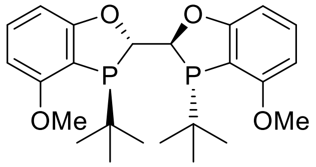	(*S*,*S*,*S*,*S*)-MeO-BIBOP	1202033-19-9	PP	$C_{24}H_{32}O_4P_2$	1.03	446.46	STREM	15-6255
L139	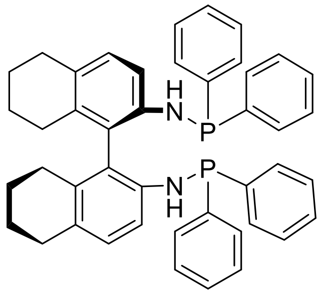	(*R*)-CTH-BINAM	208248-67-3	PP	$C_{44}H_{42}N_2P_2$	1.07	660.78	Fisher Scientific	11455420
L140	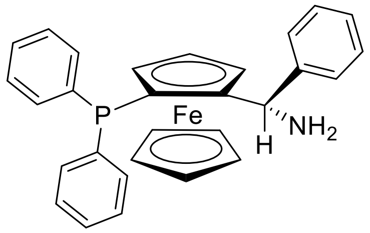	(2*R*)-1-[(*R*)-Aminophenylmethyl]-2-(diphenylphosphino)ferrocene	498580-48-6	PN	$C_{29}H_{26}FeNP$	1.09	475.35	STREM	26-1151
L141	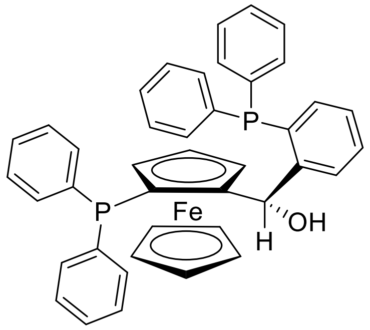	(1*R*,2*S*)-TaniaPhos-OH	851308-43-5	PP	$C_{41}H_{34}FeOP_2$	1.02	660.51	STREM	26-1160

#	Structure	Alias or name	CAS#	Type	Formula	Eq. to Rh	M_w [g mol^{-1}]	Vendor	Catalog #
L142	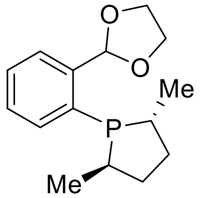	2-[2-[(2*R*,5*R*)-2,5-Dimethyl-1-phospholanyl]phenyl]-1,3-dioxolane	1044256-04-3	P	$C_{15}H_{21}O_2P$	2.09	264.30	STREM	15-7335
L143	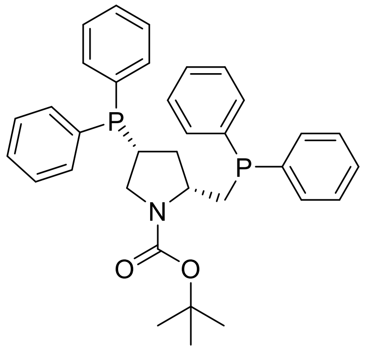	(*R*,*R*)-BPPM	72598-03-9	PP	$C_{34}H_{37}NO_2P_2$	1.02	553.62	STREM	15-7216
L144	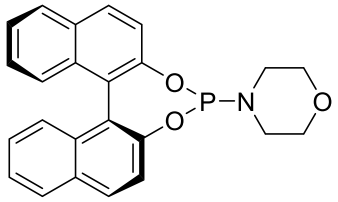	(*S*)-MorfPhos	185449-81-4	Phosphoramidite	$C_{24}H_{20}NO_3P$	2.04	401.40	STREM	15-1235
L145	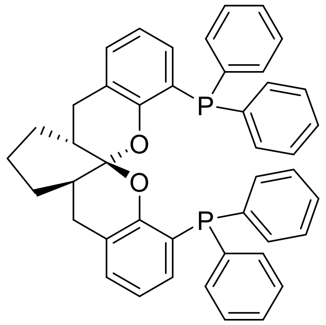	(*R*,*R*,*R*)-Ph-SKP	1360823-43-3	PP	$C_{44}H_{38}O_2P_2$	1.10	660.73	STREM	15-4310
L146	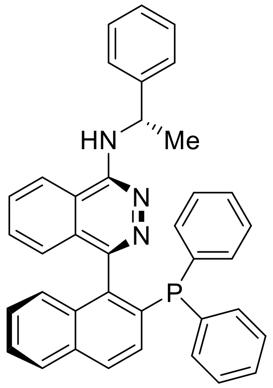	(*S*,*R*)-N-PINAP	1173836-08-2	PN	$C_{38}H_{30}N_3P$	1.08	559.65	STREM	15-1787
L147	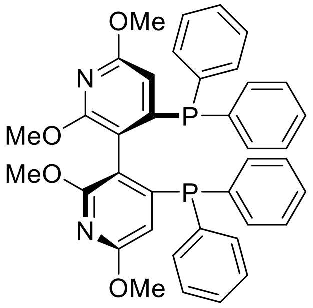	(*R*)-CTH-P-Phos	221012-82-4	PP	$C_{38}H_{34}N_2O_4P_2$	1.10	644.65	STREM	15-5200

#	Structure	Alias or name	CAS#	Type	Formula	Eq. to Rh	M_w [g mol^{-1}]	Vendor	Catalog #
L148	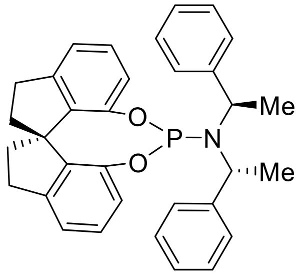	(*R*)-SIPHOS-PE	500997-69-3	Phosphoramidite	$C_{33}H_{32}NO_2P$	2.00	505.60	Sigma-Aldrich	700770
L149	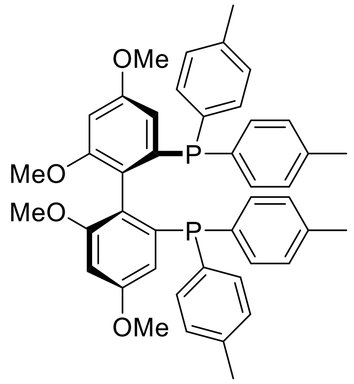	(*R*)-Tol-GarPhos	1365531-81-2	PP	$C_{44}H_{44}O_4P_2$	1.05	698.78	STREM	15-1657
L150	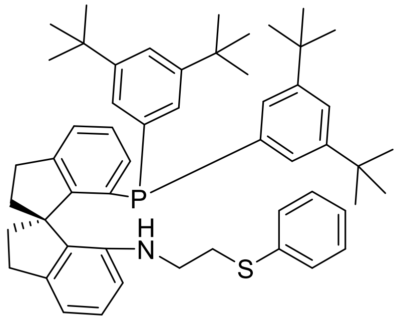	(*R*)-DTB-SpiroSAP-Ph	1809609-38-8	PNS	$C_{53}H_{66}NPS$	1.03	780.15	abcr	AB470528
L151	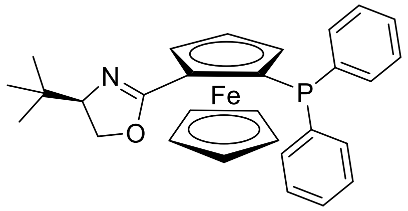	SL-N004-1	1226898-27-6	PN	$C_{29}H_{30}FeNOP$	1.03	495.38	Solvias	NA
L152	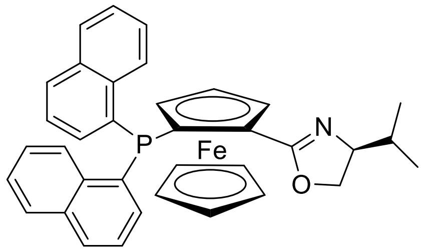	SL-N011-2	950201-43-1	PN	$C_{36}H_{32}FeNOP$	1.00	581.48	Solvias	NA
L153	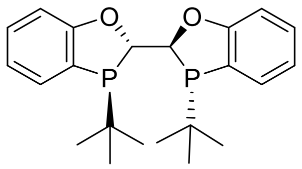	(*S*,*S*,*S*,*S*)-BIBOP	1202033-17-7	PP	$C_{22}H_{28}O_2P_2$	1.05	386.41	STREM	15-6270
L154	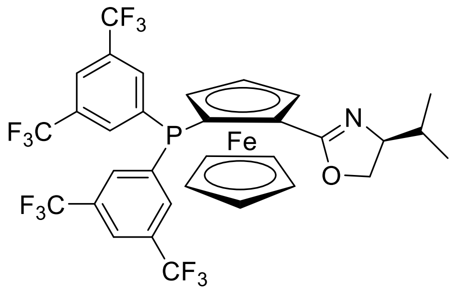	SL-N009-2	706814-27-9	PN	$C_{32}H_{24}F_{12}FeNOP$	1.01	753.35	Solvias	NA

#	Structure	Alias or name	CAS#	Type	Formula	Eq. to Rh	M_w [g mol^{-1}]	Vendor	Catalog #
L155	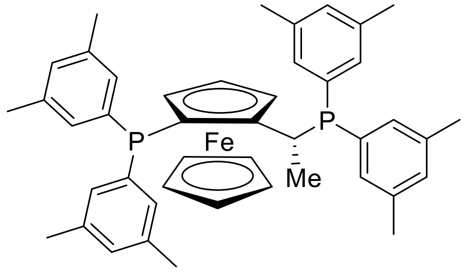	SL-J408-1	950982-69-1	PP	$C_{44}H_{48}FeP_2$	1.03	694.66	BLDpharm	BD01285898
L156	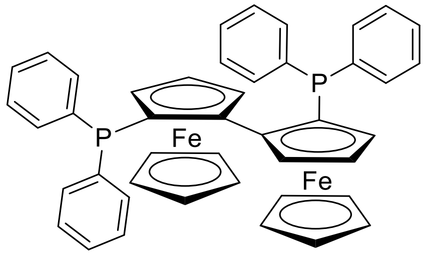	(2*R*,2*R*)-2,2-bis(diphenylphosphino)-1,1-biferrocene	136274-57-2	PP	$C_{44}H_{36}Fe_2P_2$	1.07	738.41	TCI	B3196
L157	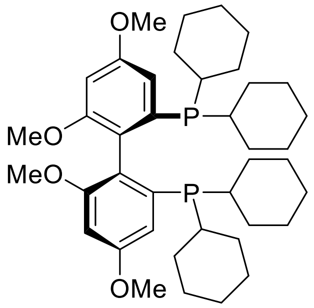	(*R*)-Cy-GarPhos	2829282-18-8	PP	$C_{40}H_{60}O_4P_2$	1.09	666.86	BLDpharm	BD01593307
L158	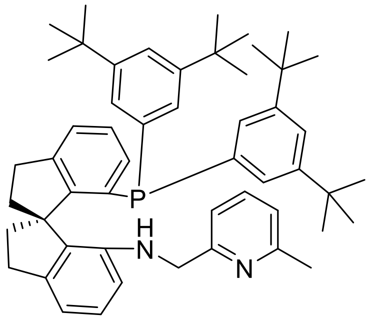	(*R*)-DTB-SpiroPAP-6-Me	1298133-26-2	PNN	$C_{52}H_{65}N_2P$	1.05	749.08	abcr	AB470532
L159	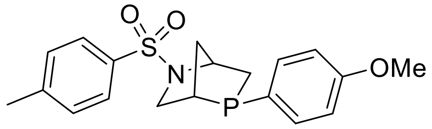	Exo-4-Methoxyphenyl Kwon [2.2.1] Bicyclic Phosphine	1975180-37-0	PN	$C_{19}H_{22}NO_3PS$	1.01	375.42	Sigma-Aldrich	798746
L160	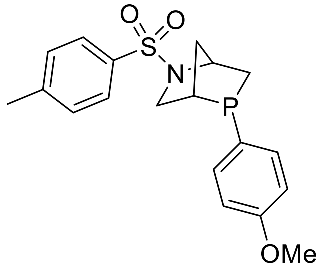	Endo-4-Methoxyphenyl Kwon [2.2.1] Bicyclic Phosphine	1883493-01-3	PN	$C_{19}H_{22}NO_3PS$	1.08	375.42	Sigma-Aldrich	798444
L161	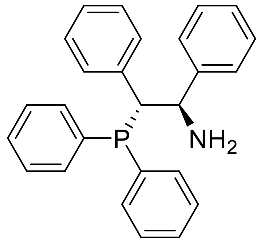	(1*R*,2*R*)-(Diphenylphosphino)phenylbenzeneethanamine	1091606-68-6	PN	$C_{26}H_{24}NP$	1.08	381.46	STREM	15-7102

#	Structure	Alias or name	CAS#	Type	Formula	Eq. to Rh	M_w [g mol^{-1}]	Vendor	Catalog #
L162	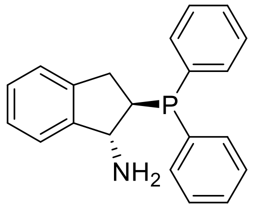	(1*R*,2*R*)-2-(Diphenylphosphino)-2,3-dihydro-1H-inden-1-amine	1091606-70-0	PN	$C_{21}H_{20}NP$	1.02	317.37	STREM	15-7110
L163	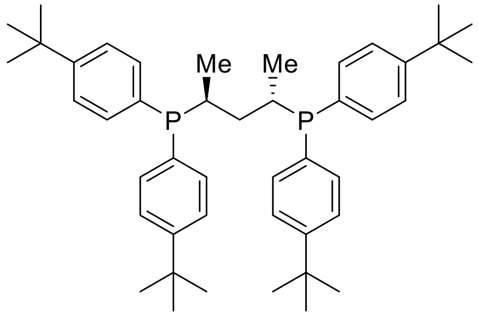	(*S*,*S*)-^{t}BuPh-SKEWPhos	911415-22-0	PP	$C_{45}H_{62}P_2$	1.07	664.94	Sinocompound	SC-4481
L164	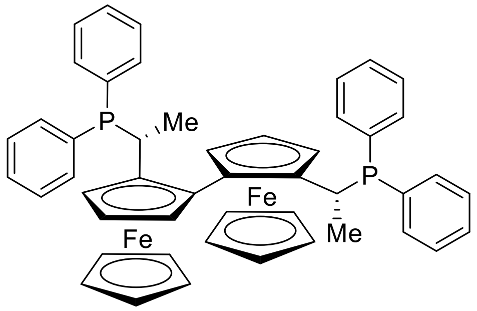	(*R*,*R*)-(*S*,*S*)-Ph-TRAP	137096-37-8	PP	$C_{48}H_{44}Fe_2P_2$	1.00	794.52	Sinocompound	SC-4624
L165	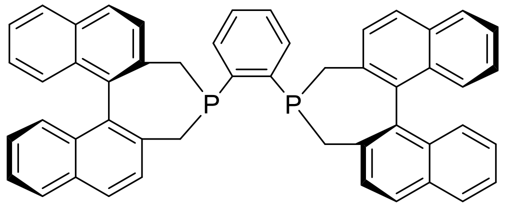	(*R*)-BINAPhane	253311-88-5	PP	$C_{50}H_{36}P_2$	1.05	698.79	Sinocompound	SC-4623
L166	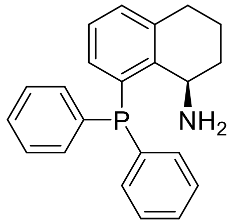	(1*R*)-8-(Diphenylphosphino)-1,2,3,4-tetrahydro-1-naphthalenamine	960128-64-7	PN	$C_{22}H_{22}NP$	1.09	331.40	Sigma-Aldrich	716685
L167	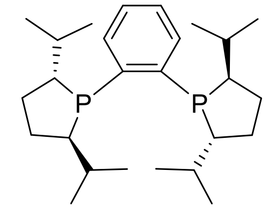	(*R*,*R*)-^{i}Pr-DuPhos	136705-65-2	PP	$C_{26}H_{44}P_2$	1.06	418.59	STREM	15-0410
L168	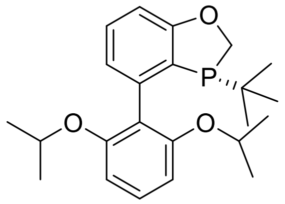	(3*R*)-4-[2,6-Bis(1-methylethoxy)phenyl]-3-(1,1-dimethylethyl)-2,3-dihydro-1,3-benzoxaphosphole	1338454-38-8	P	$C_{23}H_{31}O_3P$	2.04	386.47	STREM	15-6810

#	Structure	Alias or name	CAS#	Type	Formula	Eq. to Rh	M_w [g mol^{-1}]	Vendor	Catalog #
L169	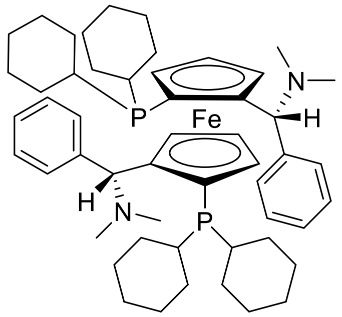	SL-M002-1	494227-35-9	PP	$C_{52}H_{74}FeN_2P_2$	1.06	844.97	STREM	26-0240
L170	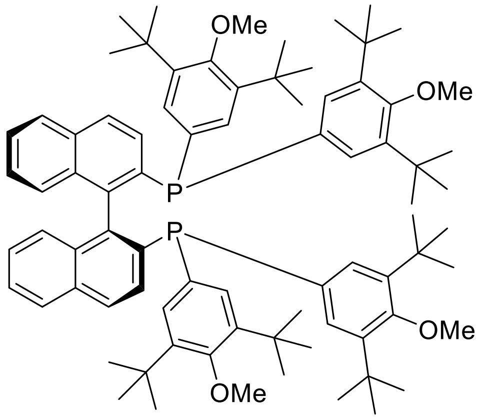	(*S*)-DTBM-BINAP	541502-07-2	PN	$C_{80}H_{104}O_4P_2$	1.06	1191.66	Sinocompound	SC-4495
L171	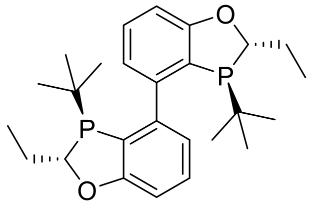	(*S*,*S*,*S*,*S*)-Et-BABIBOP	2415751-83-4	PP	$C_{26}H_{36}O_2P_2$	1.03	442.52	abcr	AB541715
L172	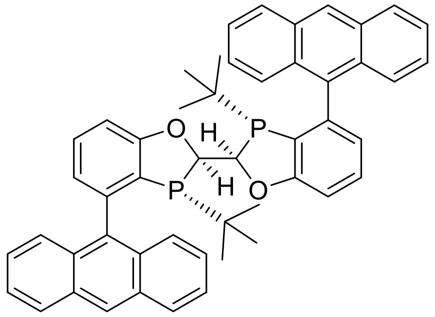	(*R*,*R*,*R*,*R*)-WingPhos	1884680-45-8	PP	$C_{50}H_{44}O_2P_2$	1.07	738.85	STREM	15-1970
L173	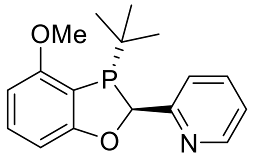	2-[(2*S*,3*S*)-3-(1,1-Dimethylethyl)-2,3-dihydro-4-methoxy-1,3-benzoxaphosphol-2-yl]pyridine	2565792-52-9	PN	$C_{17}H_{20}NO_2P$	1.07	301.33	abcr	AB549293
L174	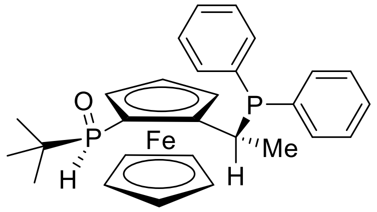	SL-J681-1	1221745-90-9	P(P=O)	$C_{28}H_{32}FeOP_2$	1.07	502.36	STREM	26-1270

#	Structure	Alias or name	CAS#	Type	Formula	Eq. to Rh	M_w [g mol^{-1}]	Vendor	Catalog #
L175	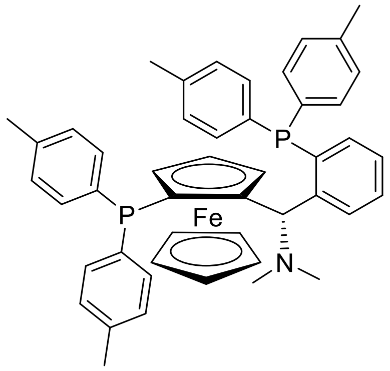	(*S*,*S*)-Tol-TaniaPhos	NA	PP	$C_{47}H_{47}FeNP_2$	1.02	743.69	Sinocompound	SC-4582
L176	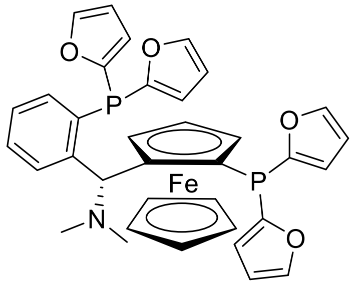	(*R*,*R*)-2-Furyl-TaniaPhos	NA	PP	$C_{35}H_{31}FeNO_4P_2$	1.04	647.43	Sinocompound	SC-4575
L177	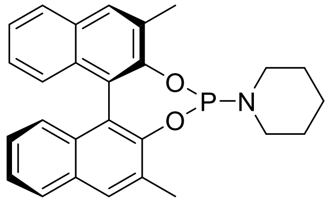	(*R*)-DM-MorfPhos	864529-90-8	Phosphoramidite	$C_{27}H_{26}NO_2P$	2.00	427.48	Ambeed	A1220020
L178	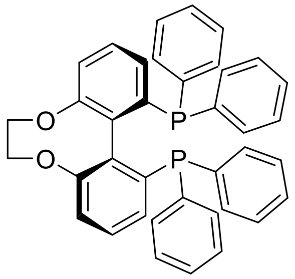	(*R*)-C$_2$-TunePhos	301847-88-1	PP	$C_{38}H_{30}O_2P_2$	1.04	580.60	Ambeed	A976565
L179	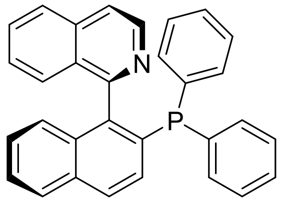	(*R*)-QUINAP	149341-34-4	PN	$C_{31}H_{22}NP$	1.03	439.50	Sigma-Aldrich	743461
L180	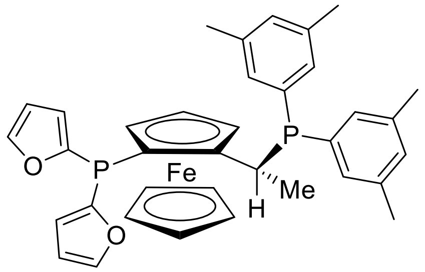	SL-J015-1	649559-65-9	PP	$C_{36}H_{36}FeO_2P_2$	1.01	618.47	BLDpharm	BD00958825

#	Structure	Alias or name	CAS#	Type	Formula	Eq. to Rh	M_w [g mol^{-1}]	Vendor	Catalog #
L181	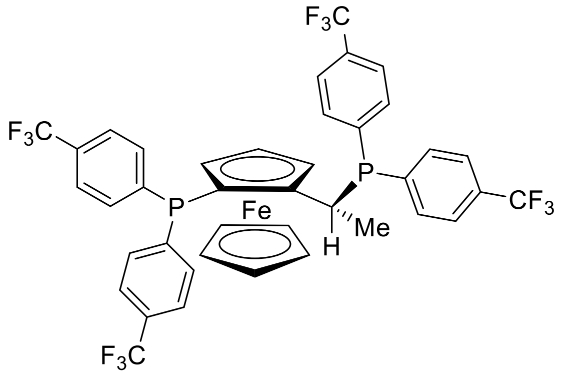	SL-J403-1	166172-60-7	PP	$C_{40}H_{28}F_{12}FeP_2$	1.02	854.45	BLDpharm	BD01153829
L182	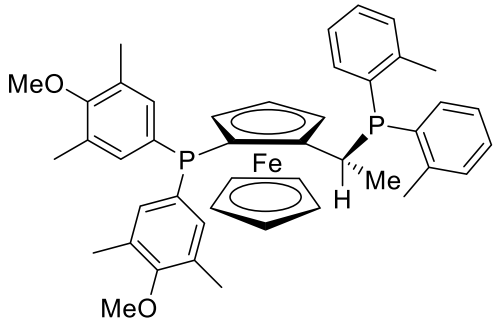	SL-J425-1	849924-49-8	PP	$C_{44}H_{48}FeO_2P_2$	1.05	726.66	BLDpharm	BD633987
L183	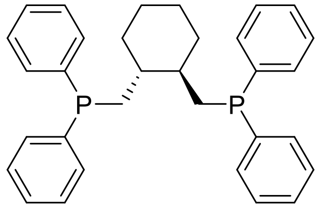	(*R*,*R*)-CyPP	70774-28-6	PP	$C_{32}H_{34}P_2$	1.07	480.57	Ambeed	A458339
L184	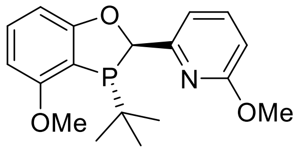	(*R*,*R*)-MeO-BoQPhos	1542796-16-6	PN	$C_{18}H_{22}NO_3P$	1.04	331.35	BLDpharm	BD01107463
L185	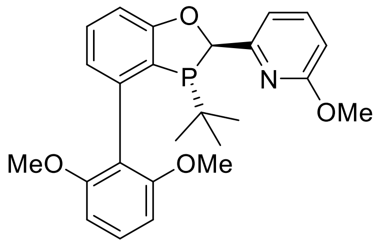	2-[(2*R*,3*R*)-4-(2,6-Dimethoxyphenyl)-3-(1,1-dimethylethyl)-2,3-dihydro-1,3-benzoxaphosphol-2-yl]-6-methoxypyridine	2565792-77-8	P	$C_{25}H_{28}NO_4P$	2.07	437.48	BLDpharm	BD01151007
L186	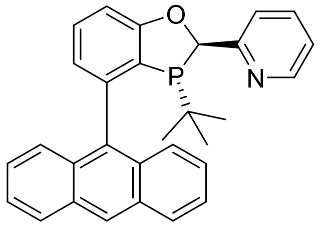	2-[(2*R*,3*R*)-4-(9-Anthracenyl)-3-(1,1-dimethylethyl)-2,3-dihydro-1,3-benzoxaphosphol-2-yl]pyridine	1542796-14-4	P	$C_{30}H_{26}NOP$	2.06	447.52	BLDpharm	BD01118981
L187	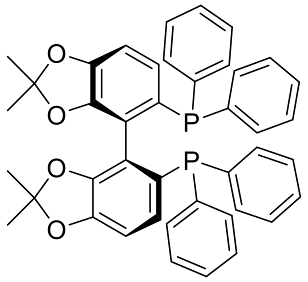	(*S*)-SunPhos	765312-54-7	PP	$C_{42}H_{36}O_4P_2$	1.02	666.69	BLDpharm	BD01178671

#	Structure	Alias or name	CAS#	Type	Formula	Eq. to Rh	M_w [g mol^{-1}]	Vendor	Catalog #
L188	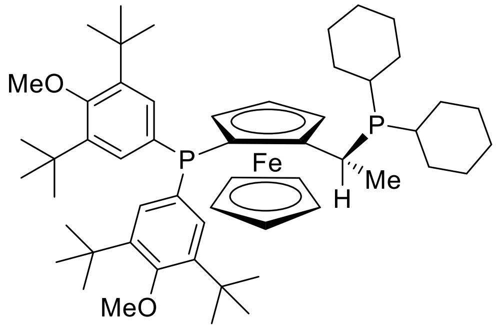	(1*R*)-1-[Bis[3,5-bis(1,1-dimethylethyl)-4-methoxyphenyl]phosphino]-2-[(1R)-1-(dicyclohexylphosphino)ethyl]ferrocene	1453803-83-2	PP	$C_{54}H_{80}FeO_2P_2$	1.07	879.02	BLDpharm	BD01227140
L189	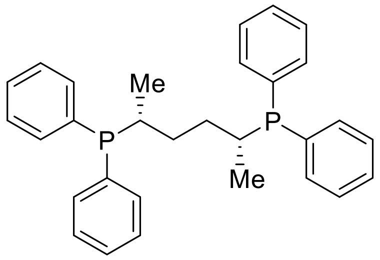	((2*R*,5*R*)-hexane-2,5-diyl)bis(diphenylphosphane)	142494-67-5	PP	$C_{30}H_{32}P_2$	1.10	454.53	BLDpharm	BD01130209
L190	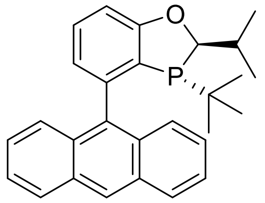	(2*R*,3*R*)-4-(9-Anthracenyl)-3-(1,1-dimethylethyl)-2,3-dihydro-2-(1-methylethyl)-1,3-benzoxaphosphole	1891002-60-0	P	$C_{28}H_{29}OP$	2.04	412.51	STREM	15-6818
L191	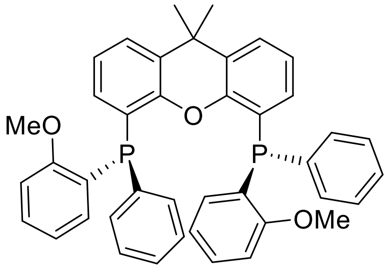	(*S*,*S*)-XantPhos	2119686-35-8	PP	$C_{41}H_{36}O_3P_2$	1.00	638.68	STREM	15-1239
L192	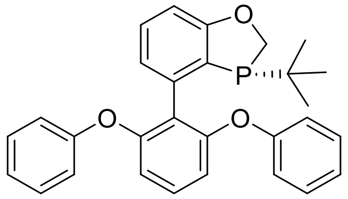	(*R*)-3-(tert-Butyl)-4-(2,6-diphenoxyphenyl)-2,3-dihydrobenzo[d][1,3]oxaphosphole	1441830-74-5	PP	$C_{29}H_{27}O_3P$	2.05	454.51	STREM	15-6814
