# Supplementary material for: Probing machine learning models based on high throughput experimentation data for the discovery of asymmetric hydrogenation catalysts
Source: Chem Sci. 2024 Jul 16. Online ahead of print. doi: 10.1039/d4sc03647f (PMC11352728; doi:10.1039/d4sc03647f)
Supplement: SC-OLF-D4SC03647F-s006 [file SC-OLF-D4SC03647F-s006.pdf]

Electronic Supplementary Material (ESI) for Chemical Science.
This journal is © The Royal Society of Chemistry 2024

Supporting Information

Probing Machine Learning Models based on High-Throughput Experimentation Data for the Discovery of Asymmetric Hydrogenation Catalysts

Adarsh V. Kalikadien,† Cecile Valsecchi,‡ Robbert van Putten,¶ Tor Maes,¶ Mikko Muuronen,¶ Natalia Dyubankova,¶ Laurent Lefort,*,¶ and Evgeny A. Pidko*,†

†*Inorganic Systems Engineering, Department of Chemical Engineering, Faculty of Applied Sciences, Delft University of Technology, Van der Maasweg 9, 2629 HZ, Delft, The Netherlands.*

‡*Discovery, Product Development and Supply, Janssen Cilag S.p.a., Viale Fulvio Testi, 280/6, 20126 Milano, Italy*

¶*Discovery, Product Development and Supply, Janssen Pharmaceutical N.V., Turnhoutseweg 30, 2340 Beerse, Belgium*

E-mail: llefort@its.jnj.com; e.a.pidko@tudelft.nl

Contents

S1 Experimental settings S3

S1.1 General considerations S3

S1.2 Experimental details S3

S1.2.1 Catalyst library preparation S3

S1.2.2 Catalyst kit preparation S4

S1.2.3 Reaction execution S4

S1.2.4 Analytical details S5

S2 Additional information on internal data analysis S8

S3 Density Functional Theory calculations S9

S4 Details of featurization S10

S4.1 Buried volumes S12

S4.2 Dihedral angles S12

S5 Literature comparison of 3D DFT-based descriptors S14

S6 Principal Component Analysis S19

S7 Linear Regression S20

S8 Details for Random Forest (RF) S21

S9 Extended partially out-of-domain approach S21

S10 Naive out-of-domain approach S22

S11 Monte Carlo in-domain approach S24

References S26

S1 Experimental settings

S1.1 General considerations

All manipulations were, unless stated otherwise, performed under inert atmosphere in a nitrogen-filled glovebox. Chemicals, pre-catalysts, and anhydrous solvents were purchased from Sigma-Aldrich, STREM, Solvias, abcr, Santa Cruz Biotechnology, Ambeed, Kanto, Fisher Scientific, TCI, Sinocompound, and BLDpharm, and were used as received. Air- and/or moisture sensitive materials were stored inside the glovebox.

S1.2 Experimental details

S1.2.1 Catalyst library preparation

Inside the glovebox, 50 μmol (~20-50 mg) chiral ligand was weighed into a 1 ml glass shell (8x30 mm, Analytical Sales and Services or V&P Scientific). This was repeated for all 192 chiral ligands. Two equivalents (100 μmol) were added for monodentate phosphines and phosphoramidites. Up to 10% overdosage was accepted, i.e., actual dosing was between 50-55 µmol.

A PTFE-coated magnetic stirring bar and 500 μl 1,2-dichloroethane (DCE) were added to each well. Plates were tumble stirred at room temperature, solubility was recorded (Y/N), and DCE was removed by parallel evaporation (Genevac EZ-2 Elite). For DCE-insoluble ligands this procedure was repeated with THF.

For complexation, a PTFE-coated stirring bar was added to each well and the metal precursor was (slurry) dispensed using a multichannel pipette (50 μl DCE well^{-1}): $[Rh(NBD)_2]BF_4$: 3.70 mg/9.90 μmol well^{-1}. Chiral ligands were dissolved/suspended in 500 μl DCE or THF. 100 μl ligand solution was dispensed to the metal precursor solution using a multichannel pipette. Reactor blocks (Para-dox, Analytical Sales and Services) were then closed and stirred overnight at room temperature (~35 °C on tumble stirrer inside glovebox). Afterwards, solvent was removed by parallel evaporation. Plates were stored inside

the glovebox and were used throughout the experimental campaign.

S1.2.2 Catalyst kit preparation

Catalysts were dissolved/suspended in 250 μl DCE (or the appropriate volume for 0.2 μmol/5 μl). A Parylene-C-coated stirring bar was added to each well (8x30 mm glass shells) and 5 μl of each catalyst solution was dispensed using a multichannel pipette (0.2 μmol catalyst per well). Care was taken to dispense to the bottom of each well. Solvent was removed from the source plates and kits by parallel evaporation. Kits were used immediately or stored inside the glovebox.

S1.2.3 Reaction execution

Pre-dispensed catalyst kits were used for all experiments. To each well a stock solution of 150 μl was added that contained the starting material (e.g., 4.4 mg well-1 of SM1 in 150 μl methanol or DCE to screen at 1 mol% Rh). The reactor block was closed with a pre-slitted PFA mat and Para-dox lid (Analytical Sales and Services). The reactor block was transferred to the parallel reactor system.

Experiments were performed in a custom-made parallel reactor system (Integrated Lab Solutions, Berlin, Germany, Figure S1). This reactor system was designed to fit four SBS-sized well plates and offers individual control over gas composition (N_2, H_2, specialty, pressure (up to 100 bar), and reaction temperature (sub-ambient to 150 °C). Stirring is performed by tumble stirring.

Upon transfer of the reactor block to the reactor, the reactor was pre-heated to 25 °C, and the reactor headspace was flushed with N_2 ($\geq$2 min at 5 l min^{-1}) while the reactor lid was closed. The headspace was then flushed with H_2 ($\geq$30 s) and pressurized to the desired pressure. Tumble stirring was then engaged to start the reaction.

At the end of the reaction test, the reactor was cooled down and vented to ambient pressure. Reaction mixtures were diluted with 200 μl methanol using an Eppendorf EpMotion

96XL semi-automated pipettor. An aliquot of the reaction mixture was removed (∼50 μl, to target concentration of ∼1 mg ml^{-1}), diluted into 500 μl methanol in a polypropylene deep well plate, and analyzed as described.

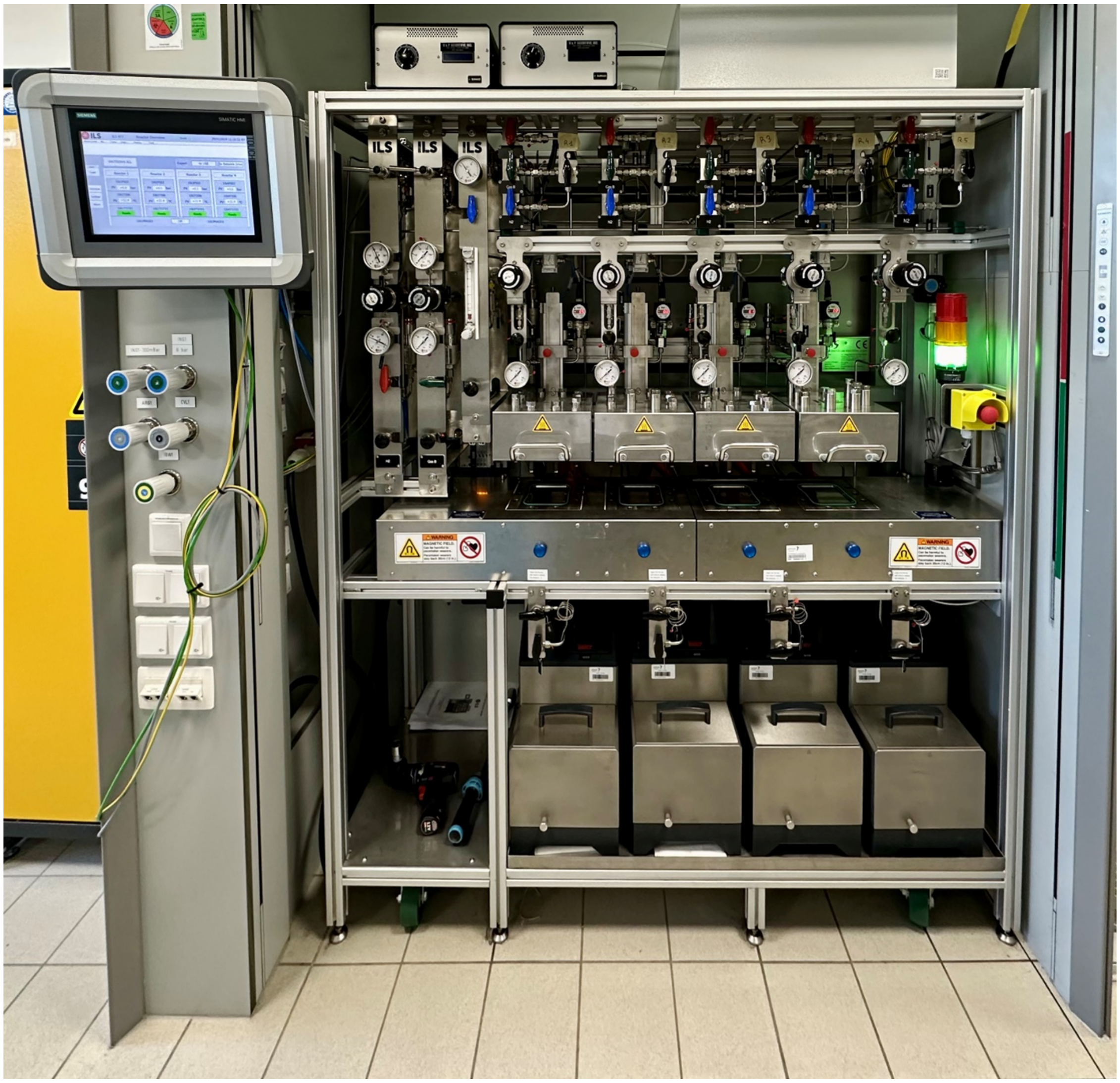

Figure S1: Parallel reactor system used in this work (Integrated Lab Solutions, Berlin, Germany).

S1.2.4 Analytical details

Measurements were performed on a Waters UPC2 SFC system equipped with PDA and MS detectors. Method details are described below. A representative chromatogram is shown in Figure S2. Chromatographic data were processed with Virscidian Analytical Studio Pro-

fessional. Product identity and absolute configuration were determined using the retention time of analytically pure reference materials. Conversion and yield were calculated using relative response factors.

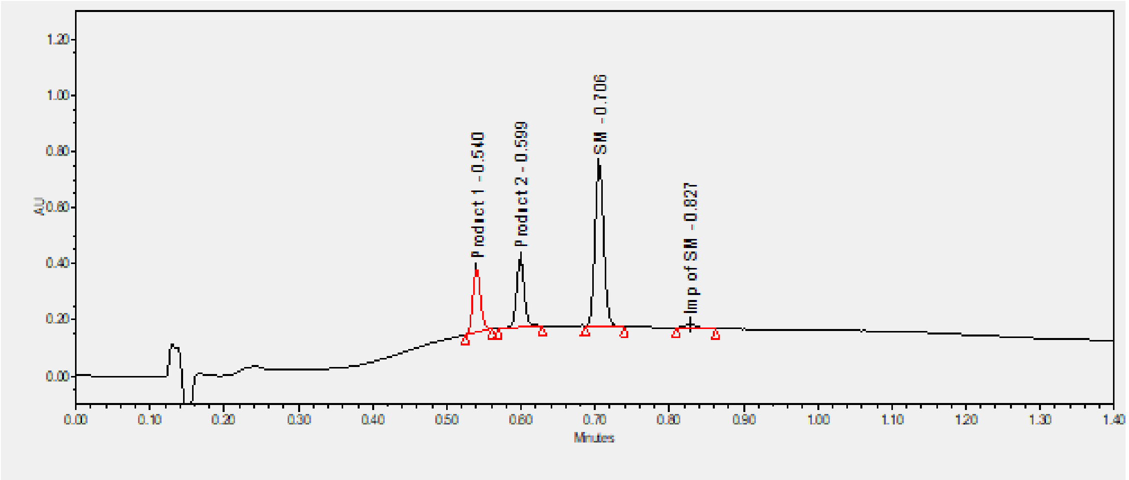

Figure S2: Representative chromatogram for SM1.

SM1: Chiralpak OZ-3 column (50x3 mm, 3 μm particle size) at 30 °C. Samples were eluted at 2.8 ml min^{-1} using CO2 (mobile phase A) and methanol (mobile phase B). Method details (gradient reported as A/B v/v): start at 97/3, ramp to 45/55 in 1.0 min, hold 45/55 for 0.5 min, ramp to 97/3 in 0.1 min (total runtime 1.4 min). MS make-up consistent of 0.450 ml min^{-1} 10 mM NH4OAc in H2O/MeOH (5/95 v/v). Chromatograms were analyzed at 204 nm. Retention times: 0.540 min ((S)-product), 0.599 min ((R)-product), 0.706 min (SM).

SM2: Chiralpak IG-3 column (150x3 mm, 3 μm particle size) at 30 °C. Samples were eluted at 1.7 ml min^{-1} using CO2 (mobile phase A) and methanol (mobile phase B). Method details (gradient reported as A/B v/v): start at 97/3, ramp to 50/50 in 3.0 min, hold 50/50 for 0.5 min, ramp to 97/3 in 0.1 min, hold 97/3 for 0.4 min (total runtime 4.0 min). MS make-up consistent of 0.450 ml min^{-1} 10 mM NH4OAc in H2O/MeOH (5/95 v/v). Chromatograms were analyzed at 203 nm. Retention times: 1.258 min (SM), 1.435 min ((R)-product), 1.924 min ((S)-product).

SM3: Chiralpak IG-3 column (50x3 mm, 3 μm particle size) at 30 °C. Samples were eluted at 2.8 ml min^{-1} using CO2 (mobile phase A) and methanol (mobile phase B). Method details

(gradient reported as A/B v/v): start at 97/3, ramp to 45/55 in 1.0 min, hold 45/55 for 0.5 min, ramp to 97/3 in 0.1 min (total runtime 1.4 min). MS make-up consistent of 0.450 ml min^{-1} 10 mM NH4OAc in H2O/MeOH (5/95 v/v). Chromatograms were analyzed at 210 nm. Retention times: 0.617 min ((R)-product), 0.651 min ((S)-product), 0.877 min (SM).

SM4: Chiralpak AD-3 column (150x3 mm, 3 μm particle size) at 30 °C. Samples were eluted at 1.2 ml min^{-1} using CO2 (mobile phase A) and methanol with 0.2% trifluoroacetic acid (mobile phase B). Method details (gradient reported as A/B v/v): start at 97/3, ramp to 50/50 in 3.0 min, hold 50/50 for 0.5 min, ramp to 97/3 in 0.1 min, hold 97/3 for 0.4 min (total runtime 4.0 min). MS make-up consistent of 0.450 ml min^{-1} 10 mM NH4OAc in H2O/MeOH (5/95 v/v). Chromatograms were analyzed at 210 nm. Retention times: 1.424 min ((S)-product), 1.451 min ((R)-product, partial overlap with (S)-product), 1.805 min (SM).

SM5: (S,S) Whelk-O 1 column (150x3 mm, 3 μm particle size) at 30 °C. Samples were eluted at 1.2 ml min^{-1} using CO2 (mobile phase A) and methanol (mobile phase B). Method details (gradient reported as A/B v/v): start at 97/3, hold 97/3 for 3.0 min, ramp to 50/50 in 0.5 min, ramp to 97/3 in 0.1 min, hold 97/3 for 0.4 min (total runtime 4.0 min). MS make-up consistent of 0.450 ml min^{-1} 10 mM NH4OAc in H2O/MeOH (5/95 v/v). Chromatograms were analyzed at 210 nm. Retention times: 1.363 min ((R)-product), 1.470 min ((S)-product), 1.529 min (SM).

S2 Additional information on internal data analysis

Three independent runs were performed with SM1 to assess the reproducibility of the approach. Results are reported in figure S4.

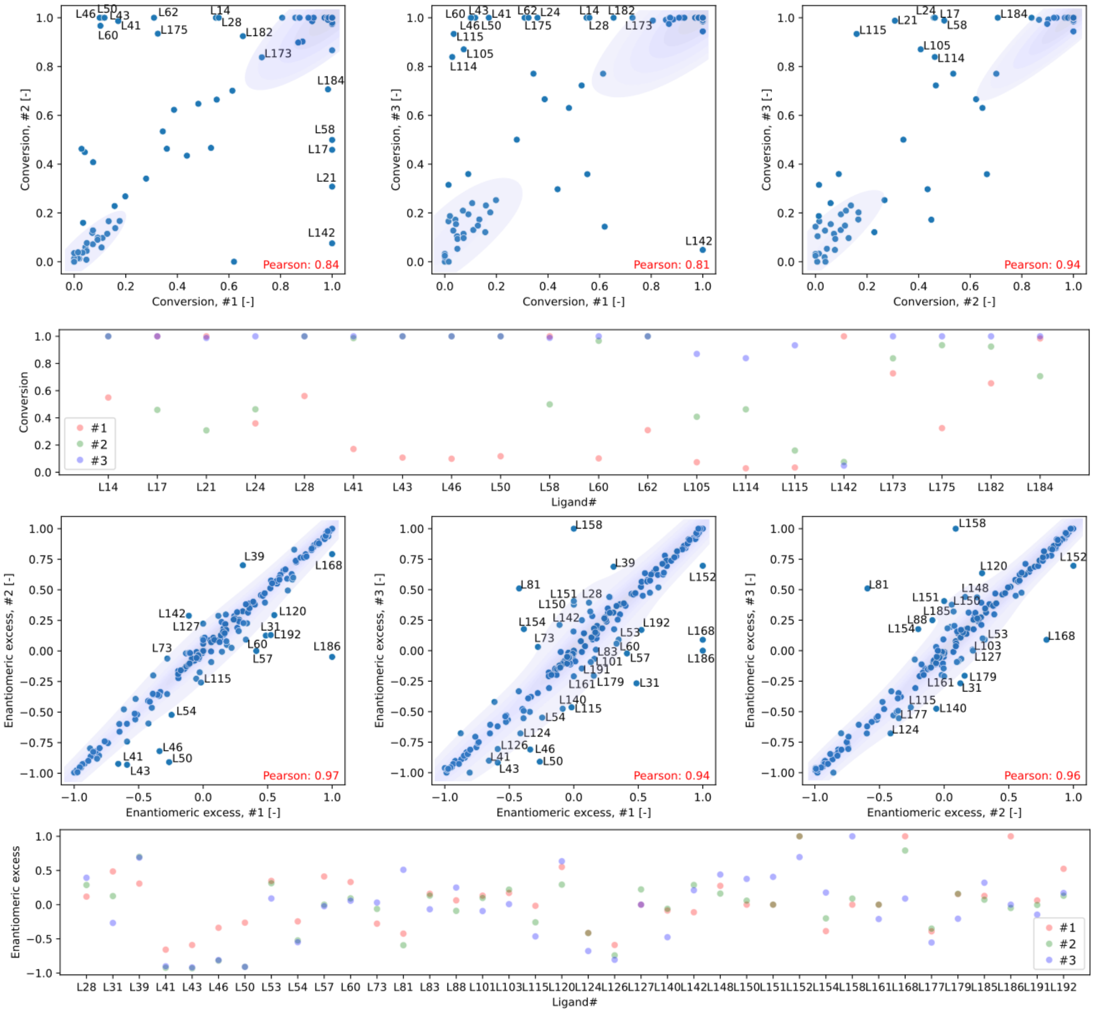

Figure S3: Comparison of conversion and enantiomeric excess across three runs (#1,#2 and #3) for SM1, 16h, Methanol. Discrepancies are labelled and illustrated more in detail in the categorical scatter plots.

Validation of the parallel pressure reactor was performed by testing the same catalyst in all the wells across the 96-wells reactor block. Standard deviation for conversion: $< 0.5\%$; ee $= 1.5\%$.

Validation ILS Parallel Reactor

Test reaction:

Asymmetric hydrogenation of α,β-disubstituted acrylic acid

In a standard autoclave, such a reaction leads to full conversion and ee of 94-96%

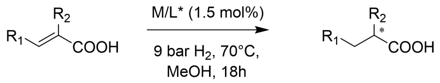

Reactor block:

8 x 12, 1mL vial, Analytical Sales reactor block with preslit mat
Same reaction mixture in all vials
10mg substrate in 150uL MeOH

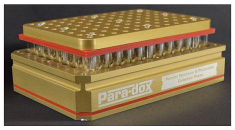

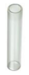

1mL vial
8 x 30mm,
flat bottom

Results:

Conversion (%)

	1	2	3	4	5	6	7	8	9	10	11	12
A	100	100	100	100	100	100	100	100	100	100	100	100
B	100	100	100	100	100	100	100	100	100	100	100	100
C	100	100	100	100	100	100	100	100	100	100	100	100
D	100	100	100	100	100	100	100	100	100	100	100	100
E	100	100	100	100	100	100	100	100	100	100	100	100
F	100	100	100	100	100	100	100	100	100	100	100	100
G	100	100	100	100	100	100	100	100	100	100	100	100
H	100	100	100	100	100	100	100	100	100	100	100	100

Full conversion obtained in all vials

Enantiomeric Excess (%)

	1	2	3	4	5	6	7	8	9	10	11	12
A	96	96	92	92	94	93	92	91	91	93	92	92
B	94	91	92	91	91	94	91	91	91	91	91	91
C	92	91	91	90	91	91	91	93	91	91	91	94
D	94	92	91	91	94	91	93	91	91	94	91	92
E	94	91	91	91	91	91	92	91	91	91	91	96
F	94	91	91	91	91	91	90	91	91	92	91	91
G	92	93	91	91	91	91	91	93	92	91	96	91
H	95	96	93	92	91	92	92	94	91	91	93	92

Enantiomeric excess varies between 91-96%.

Figure S4: Validation of the utilized parallel pressure reactor (top) resulting in a standard deviation (bottom) for conversion of $< 0.5\%$ and a standard deviation of 1.5% for ee.

S3 Density Functional Theory calculations

DFT calculations were performed using Gaussian 16 C.01 and C.02.[1] The calculations were executed at the PBE0-D3(BJ)/def2-SV(P) level in gas phase.[2–4] This combination of functional and basis set were chosen in an effort to balance computational cost and accuracy. These methods have previously been established to generate reasonable energies and structures for similar TM-based complexes.[5–8] The nature of each stationary point was confirmed via frequency analysis. Thermochemical parameters (e.g. ZPE, finite temperature corrections and entropy contributions to Gibbs free energies) were computed from analytical frequencies (Hessian) at 298.15K and 1 atm. Single point calculations on free ligands extracted from the optimized metal-ligand complexes were done at the same level of theory to

obtain ligand descriptors. A Natural Population Analysis (NPA) was performed using the NBO program version 3.1 as implemented in Gaussian 16.

S4 Details of featurization

The in-house developed Python package Open Bidentate Ligand eXplorer (OBeLiX) was used for the automated extraction and calculation of descriptors.[9] An installation and usage guide can be found in its Github repository (https://github.com/EPiCs-group/obelix). Model homogeneous catalyst structure were constructed, featuring a rhodium (Rh) metal center. The metal center was coordinated with a biphosphine bidentate ligand. Additionally, a norbornadiene (NBD) moiety was coordinated as a model substrate representative of the experimental protocol for pre-catalyst generation (Figure S5). In short, the workflow uses a graph method to find and enumerate the metal center and bidentate ligand donor atoms in these model structures. This enumeration was necessary for the calculation of local descriptors and orientation of descriptors such as quadrant/octant contributions of the buried volume. The enumeration of ligand donor atoms is based on a GFN2-xTB single-point calculation as implemented in Morfeus.[10,11] For the bidentate ligands, the two coordinating atoms were distinguished based on their charge with the label 'min'/'max' denoting the least/most positively charged atom respectively.

Most calculated descriptors are self-explanatory and readily extracted using the default settings of Morfeus[11] or cclib.[12] A detailed overview of all descriptors can be found in the 'C=C_AH_dataset.xlsx' Excel file. A more detailed explanation for the definition of the calculated dihedral angles and oriented buried volume are given in the text below.

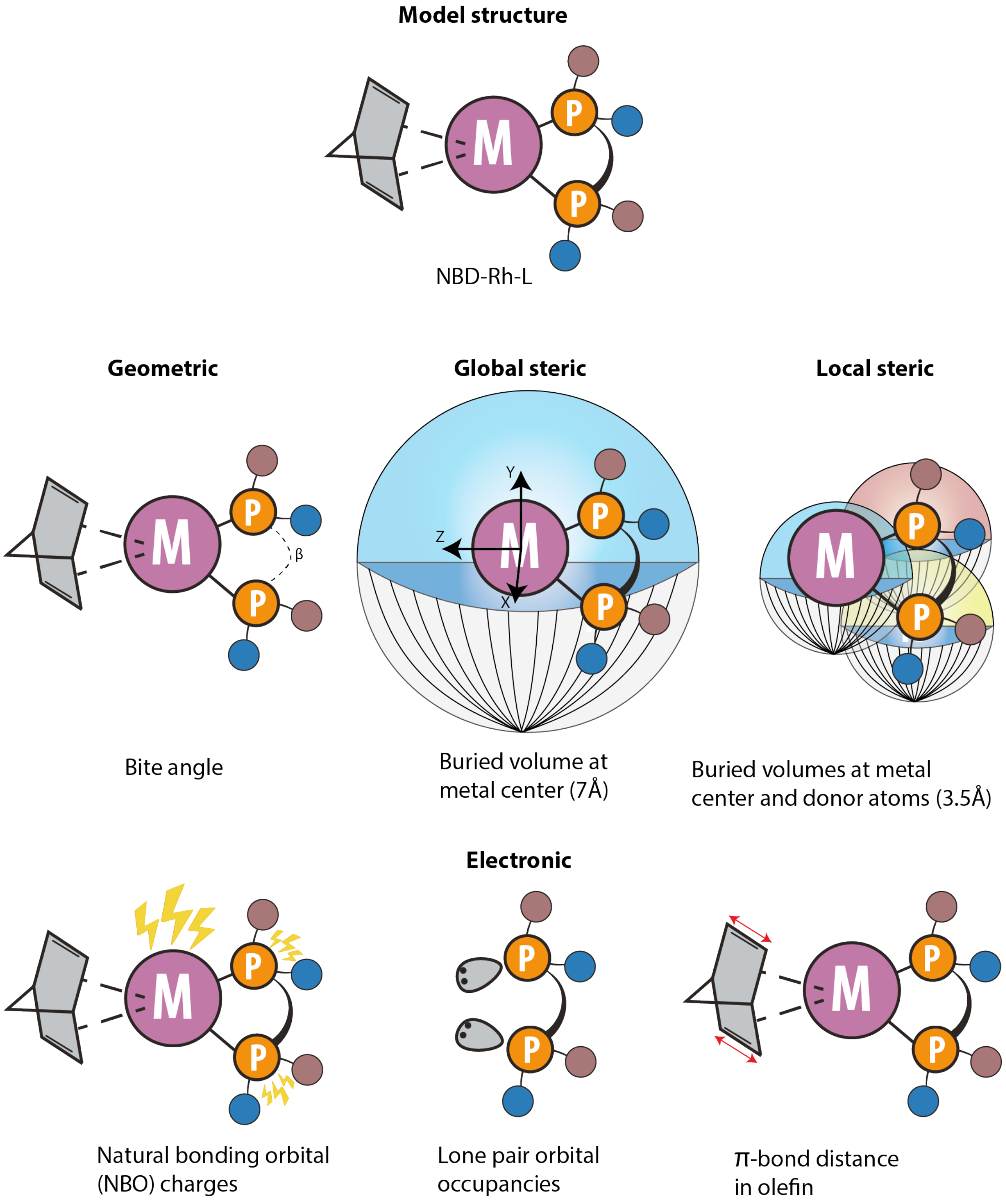

Figure S5: A selection of calculated descriptors. The precatalyst model structure is shown on the top. Examples of descriptors in various categories, such as geometric, global/local steric and electronic are also displayed.

S4.1 Buried volumes

The buried volume parameters were obtained utilizing the Morfeus package. This involved centering spheres of varying radii (ranging from 3.0 to 7.0 Å) on the Rh metal center and subsequently reporting the percentage of the sphere's volume occupied by the ligand. Additionally, the buried volume with a radius of 3.5 Å was calculated locally on both ligand donor atoms, referred to as buried_volume_donor_max and -donor_min.

Further analysis was conducted on the quadrant and octant contributions to the buried volume. These were defined using a buried volume radius of 7 Å. The donor_min and donor_max were oriented in the negative and positive x directions respectively, with the y-axis positioned perpendicular to the plane formed by donor_min, donor_max, and Rh. The quadrant buried volumes were defined by quadrants in the x,y plane, extending in both the positive and negative z directions. This approach ensures a thorough analysis of the buried volume parameters. An example of the steric maps are shown in Figure S6 for ligand L16, where the ligand structure and the oriented buried volume are shown next to each other. In this case, the P atom with index 22 (Figure S6a) is the max_donor and thus points towards the positive x-axis. In the steric plot (Figure S6b), the t-butyl groups can be identified on the right, while the phenyl groups and their respective orientation are visible on the left.

S4.2 Dihedral angles

The NBD moiety comprised a central carbon atom, which was connected to two hydrogen atoms. To assess the spatial arrangement of the hydrogen atoms with respect to the metal center and the diagonally opposite phosphorus atom, the dihedral angles between each hydrogen atom, the metal center, and the corresponding phosphorus atom were calculated (Figure S7). The dihedral angles provide insights into the spatial orientation of the substrate and potential steric interactions between the metal center and ligand in the catalyst structure.

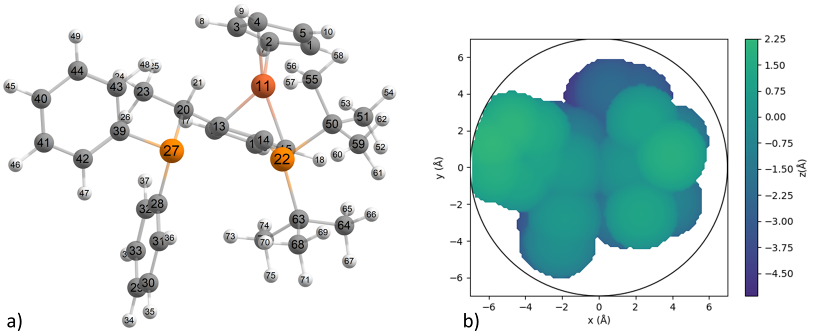

Figure S6: (a) Visualization of the 3D structure of L16, a Josiphos ligand. (b) Oriented map of the buried volume occupied by the bidentate ligand.

The figures below are of the DFT optimized structure of ligand complex L3, a Josiphos ligand, the H-C_nbd-Rh-P dihedral angle is selected in white (Figure S7a). The top view and view through the NBD molecule (Figure S7b) are shown.

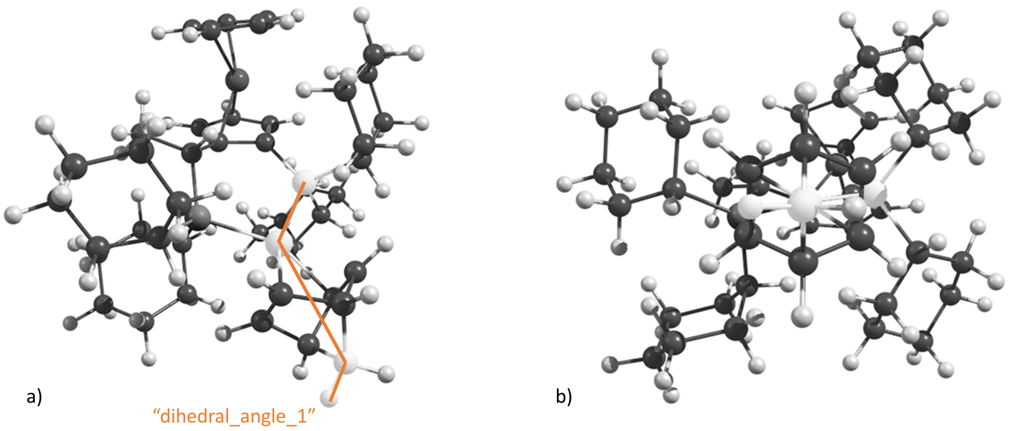

Figure S7: (a) Side view of the dihedral angle comprised of the H-C_nbd-Rh-P atoms. (b) Front view of the same dihedral angle.

S5 Literature comparison of 3D DFT-based descriptors

In this section more details are given on the comparison of the descriptor library for overlapping ligands (111 out of 192) published by Sigman's group in collaboration with Genentech.[8] The full analysis as described in this section can be found in the 'dft_nbd_model_literature_comparison.zip' in the SI which contains a Jupyter Notebook. Various aspects in the workflow to create the published descriptor library were observed to be slightly different from our study, such as the nature of the placeholder substrate and metal center (Rh(L)(NBD) in our case and $Pd(L)(Cl)_2$ in the published study), structure generation, geometry optimization, and descriptor calculation methods. Initially, we attempted to reconstruct the descriptor library via our methods to mitigate any difference in the descriptor calculation method. This was done by applying our OBeLiX workflow to the published xyz structures of catalysts. The comparison was done by extracting the subset of structures that are exactly the same, also in axial symmetry and orientation of stereocenters,

and using our own OBeLiX package to calculate descriptors on them. The xyz files of the $Pd(L)(Cl)_2$ structures were extracted, and a DFT SP calculation was performed on these structures to derive the DFT-based descriptors (vide supra). It is crucial to emphasize that no supplementary geometry optimization was conducted; therefore, the comparison is based on the structures as originally published by Dotson et al.[8] The comparative study focused on a curated selection of descriptors chosen to comprehensively represent various catalytic properties. This encompassed the calculation of descriptors both on the complex and the free ligand, providing a holistic view. Three types of comparisons were conducted:

1. Global descriptors, capturing the overall electronic structure of the ligand.

2. Local descriptors, characterizing the environment around the metal center and ligand donor atoms.

3. Spatial arrangement via the buried volume, quantifying differences in the ligand's configuration.

For global descriptors, we selected the HOMO (figure S8 A), LUMO (figure S8 B), dipole moment (figure S8 C) and bite angle (figure S8 D). Interestingly, all descriptors exhibited a Pearson correlation coefficient (R^2) exceeding 0.75, except for the dipole moment. It is noteworthy that the dipole moment is particularly susceptible to variations in the ligand's conformation, exerting a significant influence on the steric environment. This nuanced effect remains unaccounted for in the bite angle, given its constrained measurement between three points, such as P-M-P in the case of PP ligands.

To compare the local environment of the donor atoms, the NBO charge, mulliken charge and buried volume on the donor atoms of the ligand were selected (Figure S9 A,B and D). For the comparison, these descriptors were averaged over both donor atoms, implying that only the average contribution is compared. This was necessary since in the descriptor calculation, the donors are labeled based on their charge. To ease the comparison, we thus averaged the descriptor over both donors. The metal center's environment was compared

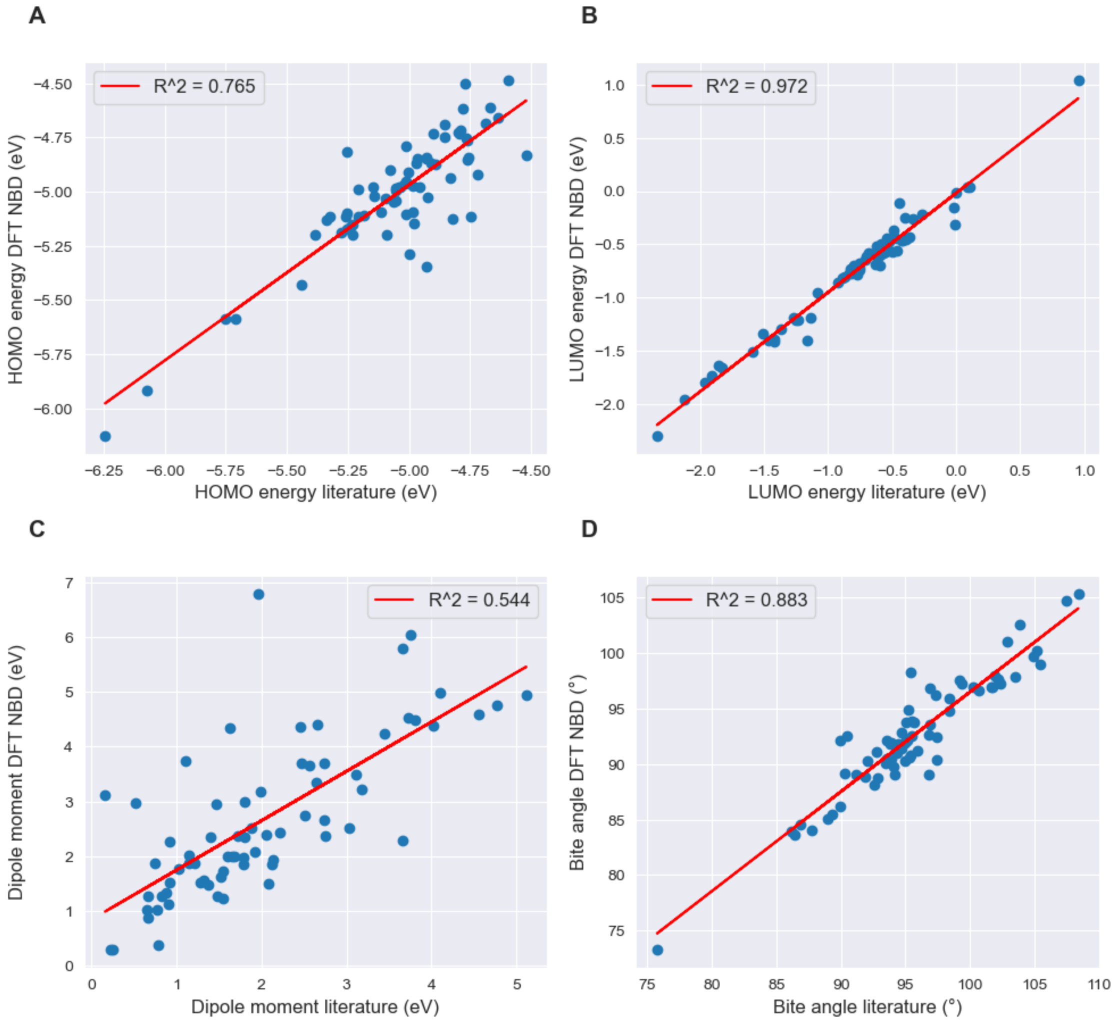

Figure S8: The figure illustrates the correlation between four global descriptors—HOMO (A), LUMO (B), dipole moment (C), and bite angle (D)—derived from ligand structures analyzed in our study, compared to descriptors derived from the same ligand structures as used in literature. The R^2 values indicate strong correlations (> 0.75) for all descriptors except dipole moment.

using a buried volume at the metal center. The electronic descriptors on the donors showed good correlations ($R^2 > 0.8$), even after removal of two extreme cases (L17 (R)-BINAM-P and L139 (R)-CTH-BINAM). The correlations for steric descriptors are significantly worse, indicating a large difference in local steric environment, both around the metal center (Figure S9C) and the donor atoms (Figure S9D).

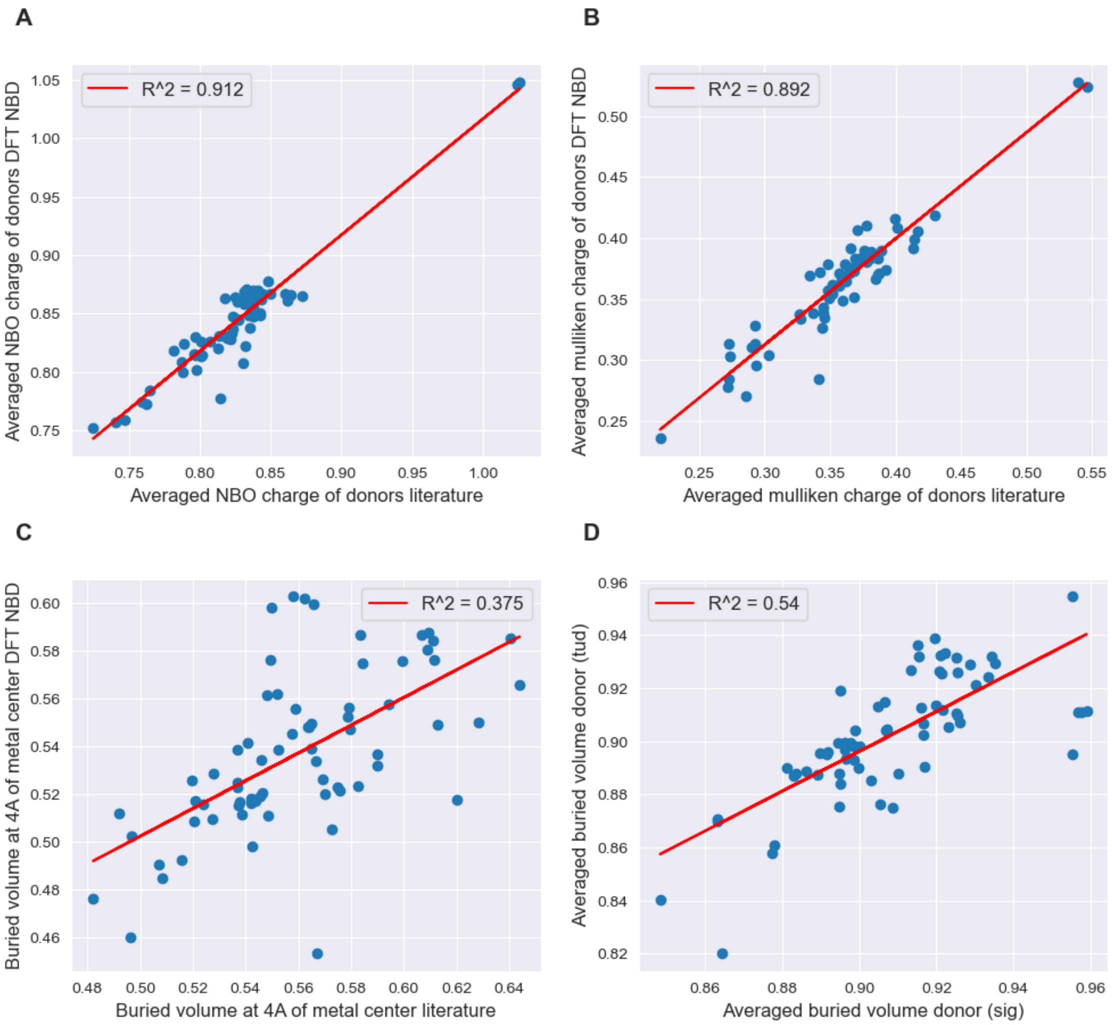

Figure S9: The figure illustrates the correlation between four local descriptors—average NBO charge of donors (A), average Mulliken charge of donors (B), buried volume at 4Å of metal center (C), and average buried volume of donors at 3.5Å (D)—derived from ligand structures analyzed in our study, compared to descriptors derived from the same ligand structures as used in literature. The electronic descriptors on the donors showed good correlations ($R^2 > 0.8$), while the steric descriptors indicate a large difference in local steric environment.

Finally, the difference in the spatial arrangement of the ligand was compared through a quadrant/octant analysis (Figure S10). To do this, the buried volume at the metal center with a radius of 7Å was separated into quadrant and octant contributions. To compare octant contributions regardless of specific orientation, we chose to compare the the minimum, maximum and average of octant contributions (Figure S10). This means that the minimum, maximum and average over the eight octants were calculated for both structures and compared. These comparisons show that although there is a trend, the correlation between the minimal contributions ($R^2 = 0.402$) and maximum contributions ($R^2 = 0.586$) are rather weak. However, the averaged octants show a reasonable correlation ($R^2 = 0.72$). This shows that although the extremes of the buried volume contributions might be different, the averages are similar. This also indicates that the local steric environment of the ligand is sensitive to small changes, e.g. to the nature of the placeholder substrate used in the metal-ligand complex (Rh(L)(NBD) in our case and Pd(L)(Cl)$_2$ in the published study).

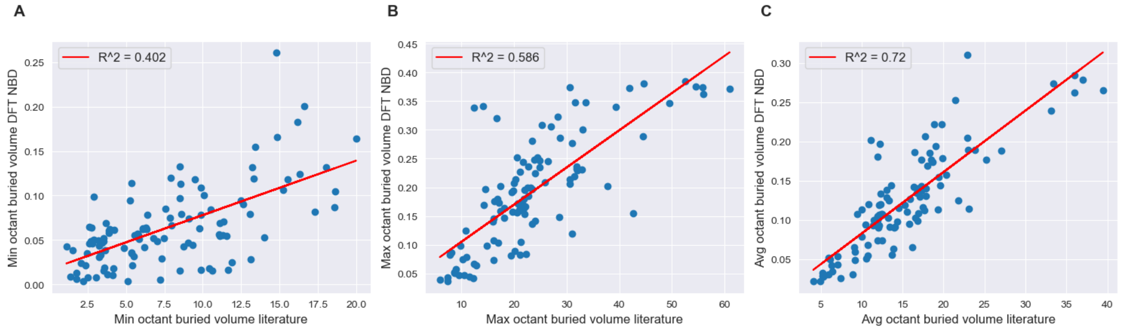

Figure S10: The figure illustrates the correlation between the octant contributions of the buried volume at the metal center with a radius of 7Å. The minimum (A), maximum (B) and average (C) over the eight octants were derived from ligand structures analyzed in our study and compared to descriptors derived from the same ligand structures as used in literature. The correlation between the averaged octants show a reasonable correlation, while the extremes show a weak correlation.

An initial direct comparison of the published descriptor library by the Sigman group, despite variations in structure generation, geometry optimization, and descriptor calculation methods, yielded satisfactory and very similar correlations for the selected descriptors. The details for this comparison can be found in the 'dft_nbd_model_literature_comparison.ipynb'

Jupyter Notebook in the SI.

S6 Principal Component Analysis

The first component explains 27% of the variance observed in the DFT NBD descriptors, while 11% of the variance is explained by the second component. In the score plot, each point is categorized by the family of ligands under investigation, such as bisphosphines, phosphine-amines, etc. This plot reveals the degree of similarity among catalysts based on their respective descriptors and three main clusters can be identified. Ligands belonging to the phosphoramidite class are distinguished by their positive values on both principal components, forming a distinct cluster in the upper right quadrant. Conversely, ligands classified as phosphine-amines are characterized by negative values on the second principal component, signifying a shared similarity in ligand properties within this category. Bisphosphine (PP) ligands are dispersed around the central region of the plot, indicating that their characteristics are representative of an average catalyst in this dataset. This observation aligns with expectations, given that bisphosphines constitute the majority of the ligands tested.

Our DFT NBD descriptors were binned into three categories: steric, geometric and electronic/thermodynamic. Correspondingly, the loading plot presented in figure SS11 is color-coded to reflect these categories. Steric descriptors correspond to negative values of the first principal component (PC1), whereas electronic descriptors are linked to extreme variations in both PC1 and PC2. This pattern allows us to infer that electronic properties are primarily responsible for differentiating the first two principal components in our analysis.

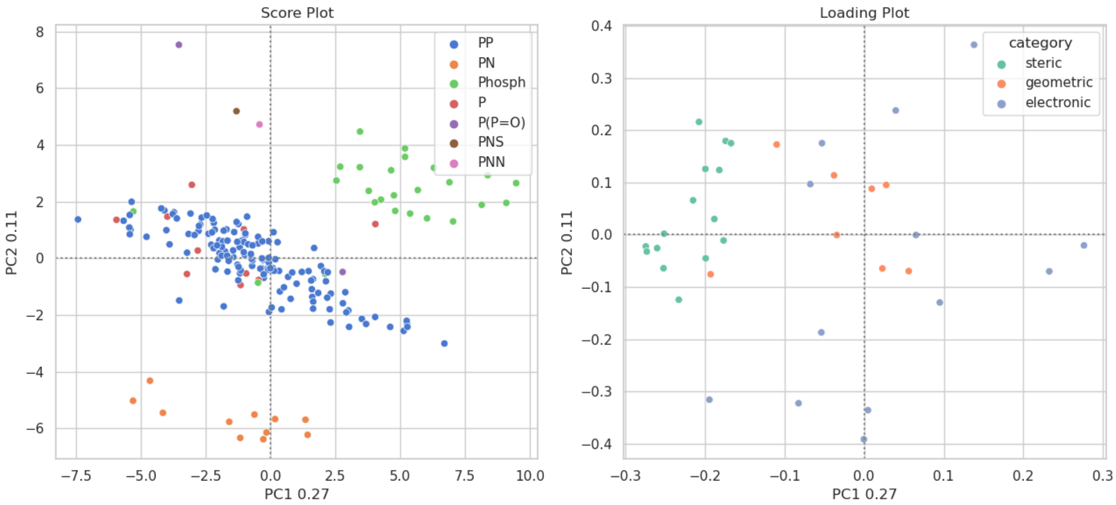

Figure S11: (left) PCA score and loading plots obtained from DFT-based descriptors, colored based on ligand families in the experimental screening set.

S7 Linear Regression

For in-domain approach we tested also Linear Regression for Conversion and DDG with up to three DFT-based descriptors (brute-force approach) for a total of 7770 linear regression models (with leave one out validation) for both regression tasks. Results are reported in Figure S12.

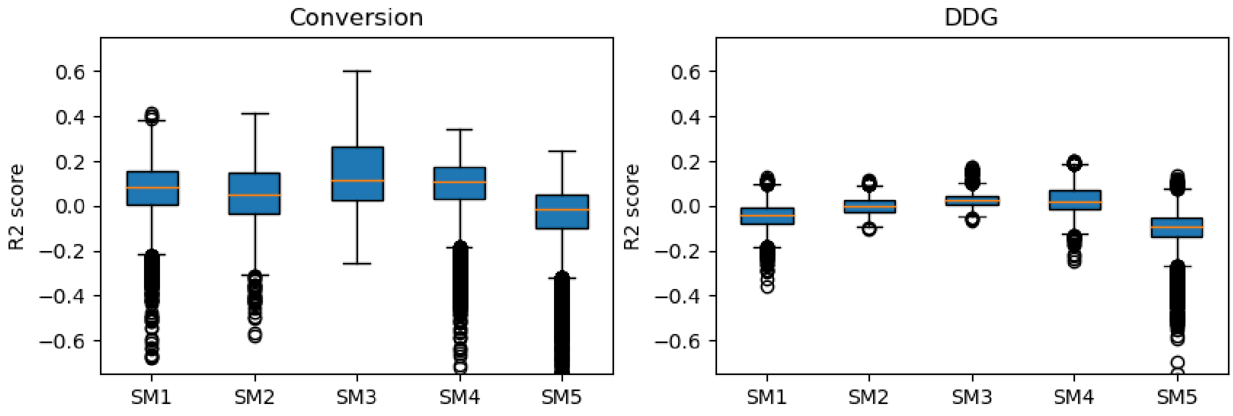

Figure S12: R2 scores distribution for brute-force in-domain Linear Regression modelling

S8 Details for Random Forest (RF)

Based on various tests with auto-ML and TPOT, the RF algorithm was suggested as a suitable non-linear model for our data. Subsequently, the RF model was implemented in our ML pipeline which can be found on our Github page (https://github.com/epics-group/obelix-ml-pipeline). The scikit-learn Python package was used for all functionalities included this pipeline. Feature importances were calculated using the deault Gini importance as implemented in SKlearn. An 80/20 train/test split was used for the out-of-domain approach, while for in-domain the median for each substrate's data was used. For each training, a 5-fold cross-validation method is applied. A grid search cross-validation method was used for selecting hyperparameters. Within this grid search, the options for each hyperparameter were:

- 'bootstrap' = [False],
- 'max_depth' = [5, 50, 100, None], *None applied only to OHE-based models
- 'max_features' = [3, 5],
- 'min_samples_leaf' = [1, 2, 5, 10],
- 'min_samples_split' = [2, 5, 10],
- 'n_estimators' = [50, 100, 200],

S9 Extended partially out-of-domain approach

In our study, we expanded the partially out-of-domain methodology with DFT-based descriptors for ligands to assess the impact of correlated substrates. Adopting a similar workflow, we trained models using not only half of the target substrate samples but also included samples from one of the other substrates, resulting in a total of 20 distinct models. The objective was to evaluate whether a correlation exists between the Pearson correlation scores observed for

experimental values (as reported in figureS13) and the balanced accuracies achieved when training on one substrate and predicting on another, across all possible substrate pairs. This approach serves as an empirical investigation into the impact of correlated substrates.

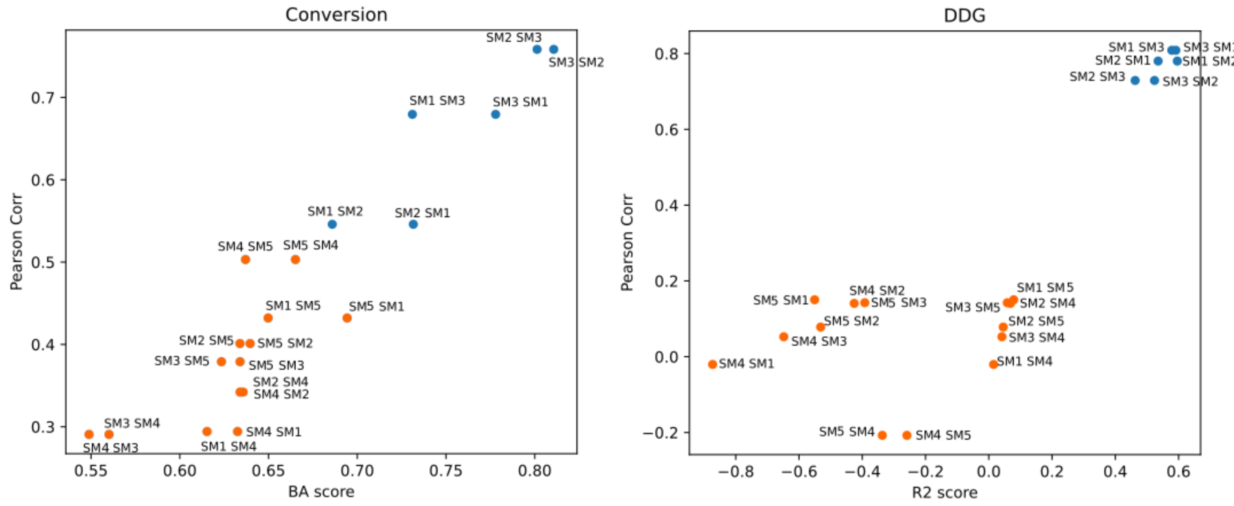

Figure S13: Balanced accuracy (BA) or R2 scores vs Pearson correlation coefficients for the extended partially out of domain approach. The first substrate in label is the target substrate while the second is the one used in the training set. Orange dots highlight pairs containing SM4 and/or SM5 in label.

S10 Naive out-of-domain approach

We analysed the generalisability of predictions of in-domain models in a naive out-of-domain fashion. Basically we compared the predictions obtained from model trained on i-th substrate with the corresponding experimental values for the j-th substrate as reported in the tables below. Clearly diagonal values report the in-domain scores as showed in the following tables, while off-diagonal values are scores obtained by the naive out-of-domain approach. We confirmed results obtained in the extended out-of-domain approach since as expected we observed higher scores for correlated substrates. Especially for reactivity prediction, BAs greater than 0.6 can be observed for naive out-of-domain predictions of the most correlated substrates: SM1,SM2 and SM3.

**Conversion**	**Balanced Accuracy**				
**dft_nbd_model**	**predictions from model trained on:**				
	**SM1**	**SM2**	**SM3**	**SM4**	**SM5**
**SM1**	0.722	0.642	0.718	0.564	0.643
**SM2**	0.650	0.652	0.673	0.570	0.554
**SM3**	0.771	0.607	0.801	0.574	0.643
**SM4**	0.516	0.484	0.490	0.779	0.622
**SM5**	0.572	0.510	0.552	0.733	0.666

**Conversion**	**Balanced Accuracy**				
**ecfp**	**predictions from model trained on:**				
	**SM1**	**SM2**	**SM3**	**SM4**	**SM5**
**SM1**	0.643	0.535	0.692	0.571	0.570
**SM2**	0.589	0.663	0.657	0.424	0.494
**SM3**	0.696	0.558	0.713	0.569	0.578
**SM4**	0.485	0.374	0.543	0.626	0.629
**SM5**	0.492	0.451	0.541	0.652	0.689

**Conversion**	**Balanced Accuracy**				
**random**	**predictions from model trained on:**				
	**SM1**	**SM2**	**SM3**	**SM4**	**SM5**
**SM1**	0.522	0.534	0.480	0.468	0.475
**SM2**	0.481	0.517	0.503	0.560	0.511
**SM3**	0.509	0.534	0.459	0.467	0.447
**SM4**	0.405	0.562	0.474	0.646	0.590
**SM5**	0.458	0.526	0.490	0.550	0.524

**DDG**	**R2 score**				
**dft_nbd_model**	**predictions from model trained on:**				
	**SM1**	**SM2**	**SM3**	**SM4**	**SM5**
**SM1**	0.092	0.128	-0.028	-0.037	-0.047
**SM2**	0.121	0.143	0.060	0.010	-0.012
**SM3**	0.095	0.198	0.078	0.027	0.021
**SM4**	-0.575	-1.380	-0.637	0.031	-0.179
**SM5**	-0.937	-2.492	-1.004	-0.244	-0.083

**DDG**	**R2 score**				
**ecfp**	**predictions from model trained on:**				
	**SM1**	**SM2**	**SM3**	**SM4**	**SM5**
**SM1**	-0.025	-0.139	-0.169	-0.073	-0.074
**SM2**	-0.002	-0.074	-0.078	0.003	-0.029
**SM3**	-0.017	-0.041	-0.036	0.013	-0.002
**SM4**	-0.053	-0.050	-0.184	-0.009	-0.049
**SM5**	-0.090	-0.153	-0.287	-0.054	-0.030

DDG	R2 score				
random	predictions from model trained on:				
	SM1	SM2	SM3	SM4	SM5
SM1	-0.032	-0.146	-0.157	-0.086	-0.095
SM2	-0.031	-0.057	-0.086	-0.023	-0.029
SM3	-0.028	-0.028	-0.066	-0.024	-0.028
4	-0.275	-0.524	-0.262	-0.040	-0.141
SM5	-0.407	-0.661	-0.443	-0.120	-0.107

S11 Monte Carlo in-domain approach

To evaluate if the best-performing models for enantioselectivity could emerge from smaller subsets of related catalyst families, a Monte-Carlo data selection approach was utilized. This method involved testing 1,000 random splits for each catalyst fraction, ranging from 90% to 10% of the entire catalyst set, in 10% decrements. Each subset was divided into an 80:20 training-test ratio, and Random Forest (RF) models were trained using DFT-based descriptors (see Figure S14). No clear pattern emerged that distinguished these high-performing subsets from the rest, such as by ligand family or class. This suggests that the high scores were likely due to chance correlations and test overfitting.

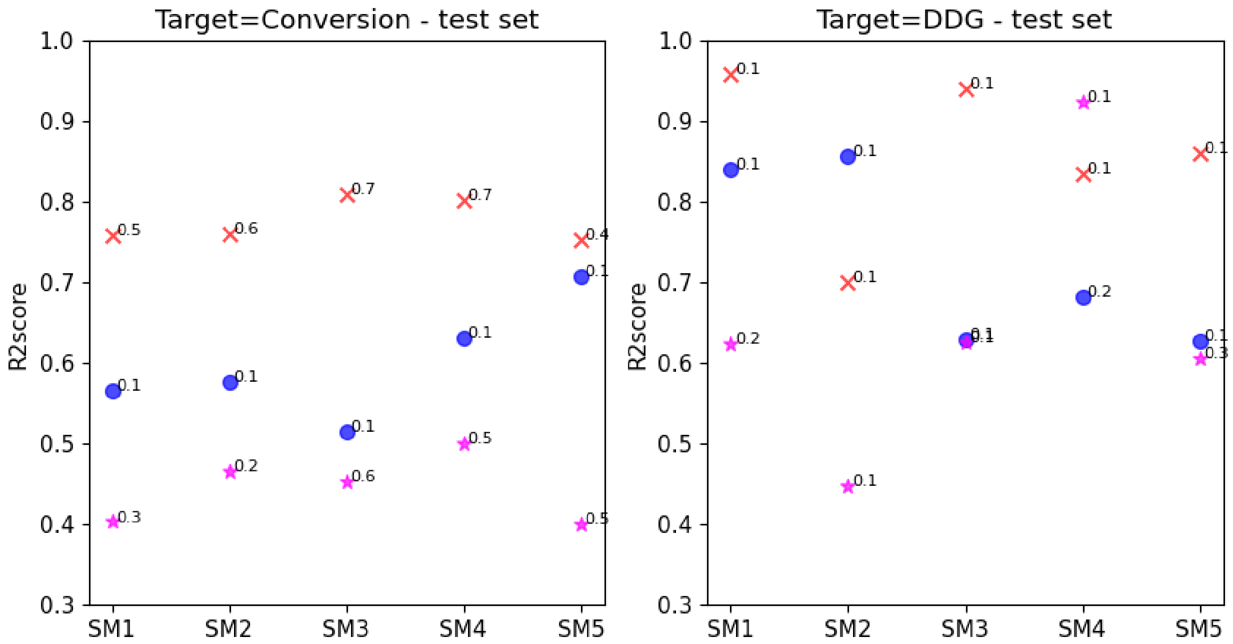

Figure S14: Monte Carlo in-domain approach, red crosses = DFT-based descriptors, blue dots= random descriptors, pink stars = ECFPs. Numbers reported refer to the fraction of ligands selected.

References

(1) Frisch, M. J. et al. Gaussian˜16 Revision C.01. 2016.

(2) Adamo, C.; Barone, V. Toward reliable density functional methods without adjustable parameters: The PBE0 model. *J. Chem. Phys.* **1999**, *110*, 6158–6170.

(3) Weigend, F.; Ahlrichs, R. Balanced basis sets of split valence, triple zeta valence and quadruple zeta valence quality for H to Rn: Design and assessment of accuracy. *Phys. Chem. Chem. Phys.* **2005**, *7*, 3297–3305.

(4) Grimme, S.; Ehrlich, S.; Goerigk, L. Effect of the damping function in dispersion corrected density functional theory. *J. Comput. Chem.* **2011**, *32*, 1456–1465.

(5) Minenkov, Y.; Sharapa, D. I.; Cavallo, L. Application of Semiempirical Methods to Transition Metal Complexes: Fast Results but Hard-to-Predict Accuracy. *J. Chem. Theory Comput.* **2018**, *14*, 3428–3439.

(6) Sinha, V.; Laan, J. J.; Pidko, E. A. Accurate and rapid prediction of pKa of transition metal complexes: semiempirical quantum chemistry with a data-augmented approach. *Phys. Chem. Chem. Phys.* **2021**, *23*, 2557–2567.

(7) Kalikadien, A. V.; Pidko, E. A.; Sinha, V. ChemSpaX: Exploration of chemical space by automated functionalization of molecular scaffold. *Digital Discovery* **2022**,

(8) Dotson, J. J.; van Dijk, L.; Timmerman, J. C.; Grosslight, S.; Walroth, R. C.; Gosselin, F.; Puntener, K.; Mack, K. A.; Sigman, M. S. Data-Driven Multi-Objective Optimization Tactics for Catalytic Asymmetric Reactions Using Bisphosphine Ligands. *J. Am. Chem. Soc.* **2022**, *145*, 110–121.

(9) Kalikadien, A. V.; Mirza, A.; Hossaini, A. N.; Sreenithya, A.; Pidko, E. A. Paving the road towards automated homogeneous catalyst design. *ChemPlusChem* **2024**, e202300702.

(10) Bannwarth, C.; Ehlert, S.; Grimme, S. GFN2-xTB—An Accurate and Broadly Parametrized Self-Consistent Tight-Binding Quantum Chemical Method with Multipole Electrostatics and Density-Dependent Dispersion Contributions. *J. Chem. Theory Comput.* **2019**, *15*, 1652–1671, doi: 10.1021/acs.jctc.8b01176.

(11) Morfeus v0.7.2: https://github.com/digital-chemistry-laboratory/morfeus.

(12) O'boyle, N. M.; Tenderholt, A. L.; Langner, K. M. cclib: A library for package-independent computational chemistry algorithms. *J. Comput. Chem.* **2008**, *29*, 839–845.
