# Supplementary material for: Probing machine learning models based on high throughput experimentation data for the discovery of asymmetric hydrogenation catalysts
Source: Chem Sci. 2024 Jul 16. Online ahead of print. doi: 10.1039/d4sc03647f (PMC11352728; doi:10.1039/d4sc03647f)
Supplement: SC-OLF-D4SC03647F-s003 [file SC-OLF-D4SC03647F-s003.zip › PCA.html]

Bokeh Plot
